# A large language model-enhanced knowledge graph framework for text-implied public health policy gap screening: digital health executability, behavioral accessibility, and service-support coverage

**DOI:** 10.3389/fpubh.2026.1937335

**Published:** 2026-09-07

**Authors:** Xinyi Wang, Jiao Lu

**Affiliations:** 1 Carey Business School, Johns Hopkins University, Baltimore, MD, United States; 2 School of Public Policy and Administration, Xi'an Jiaotong University, Xi'an, Shaanxi, China

**Keywords:** behavioral accessibility, digital health policy, evidence-linked explanation, knowledge graph, large language models, policy executability, policy-text screening, service-support coverage

## Abstract

Screening for text-implied structural gaps in policy documents is an important component of public health policy review, particularly when implementation relies on online portals, digital identity verification, remote-care platforms, or self-service processes. However, existing text classifiers, large language model prompting methods, and retrieval-based approaches often provide document-level predictions or general explanations without explicitly representing key elements, relations, and potentially missing links in policy implementation. This study proposes Policy Review Process-Knowledge Graph (PRP-KG), a large language model-enhanced knowledge graph framework for identifying structural-gap review signals across policy clauses, implementation processes, and target populations. PRP-KG segments policy documents into clauses, extracts implementation-related elements using a predefined policy-execution schema, and grounds the extracted entities and relations in source evidence spans. The validated elements are then organized into a three-layer knowledge graph. A graph-consistency feedback mechanism revises missing or schema-inconsistent triples, after which structural-gap patterns identify text-implied gaps in execution specification, accessibility safeguards, and service-support coverage. Experiments on policy-text annotation benchmarks constructed from public sources show that, under the evaluated settings, PRP-KG achieves lower prediction error and more accurate identification of high-priority text-implied review signals than most comparison methods. Controlled analyses indicate comparatively stable performance under the evaluated perturbations and provide evidence-linked outputs that can be inspected by policy reviewers.

## 1 Introduction

Digital health policies have become an important part of public health governance and healthcare service reform. Policy instruments such as telehealth, online service portals, electronic health records, patient access interfaces, digital identity authentication, and health information interoperability are widely used to improve service efficiency and expand service coverage. However, the presence of digital service provisions in a policy document does not necessarily mean that the implementation pathway is clearly specified in the text. A policy may require online access but provide limited information about responsible actors, resource conditions, service channels, offline alternatives, proxy support, or assistance for vulnerable groups. This study does not evaluate realized implementation outcomes, service utilization, or actual health equity effects. Conversely, this method focuses on issues that can be identified within the policy text and uses these signals to prioritize passages for expert document review (1–3).

In this study, “risk” refers to review signals inferred from policy texts based on pre-defined annotation standards. This task is highly challenging because the relevant signals need to be analyzed by considering the interrelationships among multiple policy elements. For instance, the requirement for digital identity verification in the policy does not necessarily imply a risk of user access or usage obstacles. It becomes a potential concern when it is linked to a target group and is not accompanied in the policy text by safeguards such as offline service windows, human assistance, telephone consultation, or proxy application channels. Therefore, a model for this task should identify policy elements, assess whether they form a complete execution pathway, and trace the resulting judgment back to the source evidence.

Traditional policy text analysis methods, including keyword matching, manually designed rules, topic models, TF-IDF representations, and shallow classifiers, can support policy topic identification and document-level categorization. These methods are transparent and inexpensive, but they typically represent policy documents using lexical or document-level features. As a result, they are not well suited to modeling relations among policy tasks, responsible actors, resources, service channels, target populations, digital barriers, and safeguards. Pre-trained and large language models improve semantic modeling for legal, regulatory, and policy-related texts, but direct classification or free-form generation often produces document-level judgments without explicitly representing the execution units underlying those judgments (4, 5). In policy review, a fluent explanation is not enough if it cannot be checked against the source text and the relation structure that supports the risk signal.

Methods for information extraction and retrieval-enhancement effectively support policy evidence positioning. Entity and relationship extraction techniques can identify key elements and their interrelationships in the policy, while retrieval-enhancement generation techniques can search for relevant policy provisions or existing organizational evidence before generating the analysis results (6, 7). However, merely locating related expressions such as “online application,” “identity verification,” or “older adults services” is still only a basic step in the analysis process. The key question is whether these fragments form an executable and accessible service pathway in the policy text, and whether a digital barrier is connected to an appropriate safeguard. Knowledge graphs and graph-based reasoning methods offer a natural way to represent typed entities, relations, paths, and missing links (8–11). Nevertheless, general graph reasoning frameworks are not usually designed around the execution logic of digital health policy documents, such as task–actor–resource–channel–target pathways or barrier–safeguard mitigation relations.

Digital health equity research further motivates this screening task. Older adults, people with low digital literacy, rural residents, people with disabilities, low-income groups, and linguistic minorities may face barriers related to device and network access, identity authentication, platform complexity, language accessibility, and health information comprehension (1–3, 12, 13). Policies may mitigate these barriers through offline service windows, human or community assistance, telephone consultation, proxy application channels, and related safeguards. Efficient policy prescreening therefore requires a structured method that identifies implementation elements, assesses whether barriers and safeguards are connected to relevant target groups, and provides evidence-linked results for expert review.

To address this need, this study proposes Policy Review Process-Knowledge Graph (PRP-KG), a large language model-enhanced Policy–Implementation–Population Knowledge Graph framework for screening text-implied review signals in digital health policies. Rather than introducing a new graph neural architecture or large language model, PRP-KG provides a schema-coupled protocol that integrates constrained extraction, evidence-span binding, graph validation, and structural-gap scoring. It extracts policy tasks, actors, resources, service channels, target populations, digital barriers, and safeguards from policy clauses. It organizes the validated elements into a three-layer graph covering policy clauses, implementation support, and target populations. Graph-consistency feedback then guides the reprocessing of incomplete or structurally incompatible relations.

PRP-KG generates three evidence-linked review signals: executability, which evaluates whether a policy task is connected to responsible actors, required resources, and an execution pathway; behavioral accessibility, which examines whether digital requirements and relevant safeguards are linked to target populations; and text-implied service-support coverage, which assesses the extent and balance of support specified for different groups. The experiments evaluate element extraction, graph construction, review-signal prediction, ablation, robustness, and explanation quality. The principal contributions are: (1) a Policy–Implementation–Population graph representation for digital health policy review; (2) risk-template-constrained extraction with source-evidence binding; (3) graph-consistency feedback for relation validation; and (4) structural-gap scoring with traceable explanation subgraphs. These signals are intended to prioritize passages for professional review and do not measure actual policy implementation, service utilization, accessibility, or health equity outcomes.

## 2 Related work

This section reviews studies on screening for text-implied structural risk in digital health policy documents. Existing research is examined from five perspectives: traditional policy text analysis, pre-trained and large language models for legal and policy text understanding, information extraction and retrieval-augmented evidence acquisition, knowledge graphs and graph-based reasoning, and digital health accessibility and service-support coverage. Particular attention is also given to medical large language models and Responsible AI concerns, including unsupported inference, hallucination, bias, transparency, accountability, and the need for human oversight in health-related text analysis. Existing studies have achieved considerable results in areas such as text representation, evidence location, relationship modeling, and policy text analysis. However, these methods still struggle to directly meet the research requirements of this study. This study focuses more on whether the implementation logic in the policy text is complete, including whether the key elements required for implementation have been clearly defined, whether a clear implementation path has been formed, whether digital barriers are accompanied by corresponding safeguard measures, and whether the final review signals can be traced back to specific policy bases.

### 2.1 Traditional policy text analysis and rule-based evaluation

Traditional policy text analysis often relies on keyword matching, manually crafted rules, topic models, TF-IDF features, and shallow classifiers. Methods such as LinearSVC, logistic regression, naive Bayes, and topic modeling are useful for identifying policy topics or for document-level categorization. They are transparent, inexpensive, and suitable as basic baselines. However, they typically represent policy documents through lexical or document-level features. This limits their ability to model relations among policy tasks, responsible actors, resources, service channels, target populations, digital barriers, and safeguards. Rule-based evaluation can check whether predefined terms appear in a policy document, but term presence alone does not indicate whether these elements form an executable, accessible, and adequately supported service pathway.

### 2.2 Pre-trained language models and large language models for legal and policy text understanding

Pre-trained language models have improved understanding of legal and policy text by providing contextual representations for classification, retrieval, and long-document processing. Encoder-based models such as BERT, LEGAL-BERT, and Longformer are therefore useful baselines for testing whether contextual document representations are sufficient for text-implied policy-text review-signal screening. Large language models further support open-ended interpretation, summarization, and reasoning. Benchmarks such as LegalBench and LawBench show that legal reasoning involves diverse capabilities and should not be reduced to simple document classification (14, 15). Prompt-based LLM methods can directly produce review signals, chain-of-thought prompting can provide rationales, and self-consistency can reduce instability in single-generation. However, free-form explanations may not provide stable evidence binding or relation-template validation. In this study, LLM baselines are not treated as weak methods. They are used to test whether a policy execution schema, evidence-span binding, and graph validation add value beyond direct generation.

Medical LLM and medical-chatbot studies further indicate that fluent responses do not necessarily ensure factual accuracy, safety, fairness, or appropriate use in health-related settings (16, 17). Responsible deployment therefore requires attention to hallucinations, unsupported inferences, algorithmic bias, transparency, accountability, privacy, and human oversight. These concerns are also relevant to digital health policy analysis, even though PRP-KG does not provide clinical advice or process patient data. In this study, evidence-span binding and relation validation improve traceability, but they do not guarantee factual correctness, causal validity, regulatory compliance, or the absence of bias. PRP-KG is therefore positioned as a human-reviewed document-screening framework rather than an autonomous policy assessment or decision-making system.

### 2.3 Information extraction and retrieval-augmented evidence acquisition

Information extraction methods identify entities, relations, and events in unstructured text. Standard extraction models, together with recent universal and LLM-assisted extraction methods, can identify policy-related elements such as tasks, actors, resources, service channels, target populations, barriers, and safeguards. Recent work on schema-flexible and instruction-guided extraction, including GCIE and ChatUIE, further shows that extraction can benefit from explicit task schemas and generation constraints (18, 19). These studies are closely related to PRP-KG because reliable policy screening depends on correctly identifying policy elements and relations.

However, extraction alone is insufficient for the task considered here. Extracting “online service portal,” “identity authentication,” “older adults,” and “community assistance” does not determine whether authentication functions as a digital barrier, whether older adults are the affected target group, or whether community assistance mitigates that barrier. These roles depend on relation validation and policy execution logic.

Retrieval-enhanced generation technology offers a new approach to obtaining policy evidence. Methods such as BM25-RAG, Dense-RAG, Evidence-aware RAG, and GraphRAG can retrieve relevant clauses from policy texts and organize the evidence to generate results. In recent years, research has gradually expanded into directions such as retrieval control, hallucination detection, long text processing, and result verification (20–23). However, retrieval is only a part of policy analysis. Finding relevant clauses does not complete the review-signal evaluation; it still requires further analysis of the evidence by considering the policy implementation process, the corresponding relationship between digital barriers and safeguard measures, and the support needs of different target groups.

### 2.4 Knowledge graphs and graph-based structured reasoning

Knowledge graphs can organize policy entities and their relationships into a unified structure, enabling scattered text information to establish clear connections. Compared with pure text representation, it is more conducive to expressing the associations between different types of entities, and also supports path queries, rule validations, and evidence-based explanations. Simultaneously, graph neural networks such as R-GCN and HGT provide mature methods for learning representations of multi-relation heterogeneous graphs and offer technical support for the further modeling of knowledge graphs. These methods are useful baselines for policy-element graphs, especially when policy information is represented as typed nodes and edges.

Recent graph-based long-context and graph-retrieval methods show that graph structures can help organize long documents and relational evidence. GraphReader constructs graphs from long texts and explores them via an agent-like process, while HippoRAG combines LLMs, knowledge graphs, and graph-based retrieval for deeper evidence integration (24, 25). Legal and policy knowledge graph studies are even closer to this work. LLM-assisted pipelines have been used to construct legislative knowledge graphs, and LegisSearch organizes legislative documents into queryable graph structures (26, 27). Other studies combine LLMs with knowledge graphs for structured reasoning, including Think-on-Graph, Reasoning on Graphs, and KG-RAG (28–30).

PRP-KG builds on this line of work but has a narrower goal. It does not propose a new graph neural architecture or a general KG reasoning algorithm. Its contribution lies in a schema-coupled screening protocol for digital health policy texts. This schema explicitly models the policy implementation process, linking key elements such as tasks, execution entities, resources, service channels, and target groups. It also describes the corresponding relationship between digital barriers and safeguard measures. The resulting graph organizes extracted policy elements and supports rule-based screening for text-implied execution gaps, unmatched behavioral accessibility, and incomplete service-support coverage. These graph-derived signals remain dependent on the source documents, extraction quality, predefined schema, and validation rules; they should not be interpreted as verified evidence of real-world implementation failure or inequity.

### 2.5 Digital health accessibility and service-support coverage

The research on digital health policies has focused on topics such as telemedicine, electronic health records, online service platforms, patient services, health information interoperability, and digital identity authentication. These digital tools, while enhancing service efficiency and coverage, may also create new barriers for certain groups, including the older adults, those with low digital literacy, rural residents, people with disabilities, low-income individuals, and language minority groups. These barriers can stem from insufficient equipment and network conditions, or relate to identity authentication requirements, complex platform operations, limited language support, or insufficient understanding of health information.

Research on digital health equity generally holds that policy design should provide digital services and consider whether different groups have the conditions and capabilities to use these services. Recent reviews highlight digital literacy support, inclusive implementation, infrastructure readiness, and service mechanisms for underserved groups (12, 13). Offline service windows, telephone consultation, community assistance, family doctor support, proxy application channels, and human guidance may reduce behavioral barriers. Work on digital health intervention co-design also stresses participant involvement, implementation context, and inclusive service delivery (31, 32).

The task studied here differs from real-world health equity evaluation. It does not measure service utilization, patient outcomes, or realized disparities after implementation. Instead, it examines whether policy texts provide sufficient execution support, accessibility safeguards, and balanced support coverage for target groups. Existing digital health equity studies define relevant concerns, but most do not provide automated, evidence-linked policy-text screening systems. PRP-KG addresses this gap by modeling Task, Actor, Resource, Channel, Target, Barrier, and Safeguard elements, validating their relations in a policy graph, and mapping missing or weak execution links to text-implied review signals. Consistent with Responsible AI principles, these signals are intended to direct human reviewers to potentially incomplete policy-text structures and to their supporting evidence spans, rather than to replace contextual policy interpretation or determine whether a policy is equitable, effective, or legally compliant.

## 3 Methodology

PRP-KG is a Policy–Implementation–Population Knowledge Graph framework for text-implied structural risk screening in digital health policy documents. It does not evaluate real-world implementation outcomes or actual service-support coverage. Instead, it identifies review signals from policy texts, including incomplete execution pathways, unmitigated digital barriers, and weak support coverage for target groups. PRP-KG contains three components: risk-template-constrained extraction, graph construction with consistency feedback, and structural-gap-driven scoring.

### 3.1 Problem formulation

Let D denote a collection of digital health policy documents. Each document $d_{i}\in D$ is segmented into clauses as Equation 1:


$$\begin{array}{l} D={\left\{d_{i}\right\}}_{i=1}^{N},\;d_{i}={\left\{c_{i,j}\right\}}_{j=1}^{M_{i}}. \end{array}$$ (1)

PRP-KG uses a policy execution schema


$$\begin{array}{l} S=\left(T_{e},T_{r},C\right), \end{array}$$

Where $T_{e}$ is the entity-type set, $T_{r}$ is the relation-type set, and Ccontains the consistency rules. The entity types include Task, Actor, Resource, Channel, Target, Barrier, and Safeguard. The relation types include assigned_to, requires, delivered_through, targets, affects, supports, and mitigates.

A policy execution unit is represented as Equation 2


$$\begin{array}{l} \begin{array}{c} z=\left(t,a,r,c,h,gh\right), \end{array} \end{array}$$ (2)

Where *t*, *a*, *r*, *ch*, *g*, and *h* denote a policy task, responsible actor, required resource, service channel, target population, and safeguard, respectively. A structurally complete unit connects a task to its actor, required resources, service channel, and target population and, when a digital barrier is identified, to an applicable safeguard.

For each document *d*_*i*_, PRP-KG produces a three-dimensional review-signal vector and a set of evidence-linked explanation paths as Equation 3 and 4:


$$\begin{array}{lll} \left(R_{i},P_{i}^{\exp}\right) & = & F\left(d_{i};S\right), \end{array}$$ (3)

$$\begin{array}{lll} R_{i} & = & \left[R_{i}^{exe},R_{i}^{acc},R_{i}^{\sup}\right], \end{array}$$ (4)

Where $R_{i}^{exe}$, $R_{i}^{acc}$, and $R_{i}^{\sup}$ denote the executability, behavioral-accessibility, and service-support-coverage review signals, respectively. $P_{i}^{\exp}$ contains source-linked graph paths and identified structural gaps supporting these signals.

### 3.2 Overall framework

**Figure 1:**
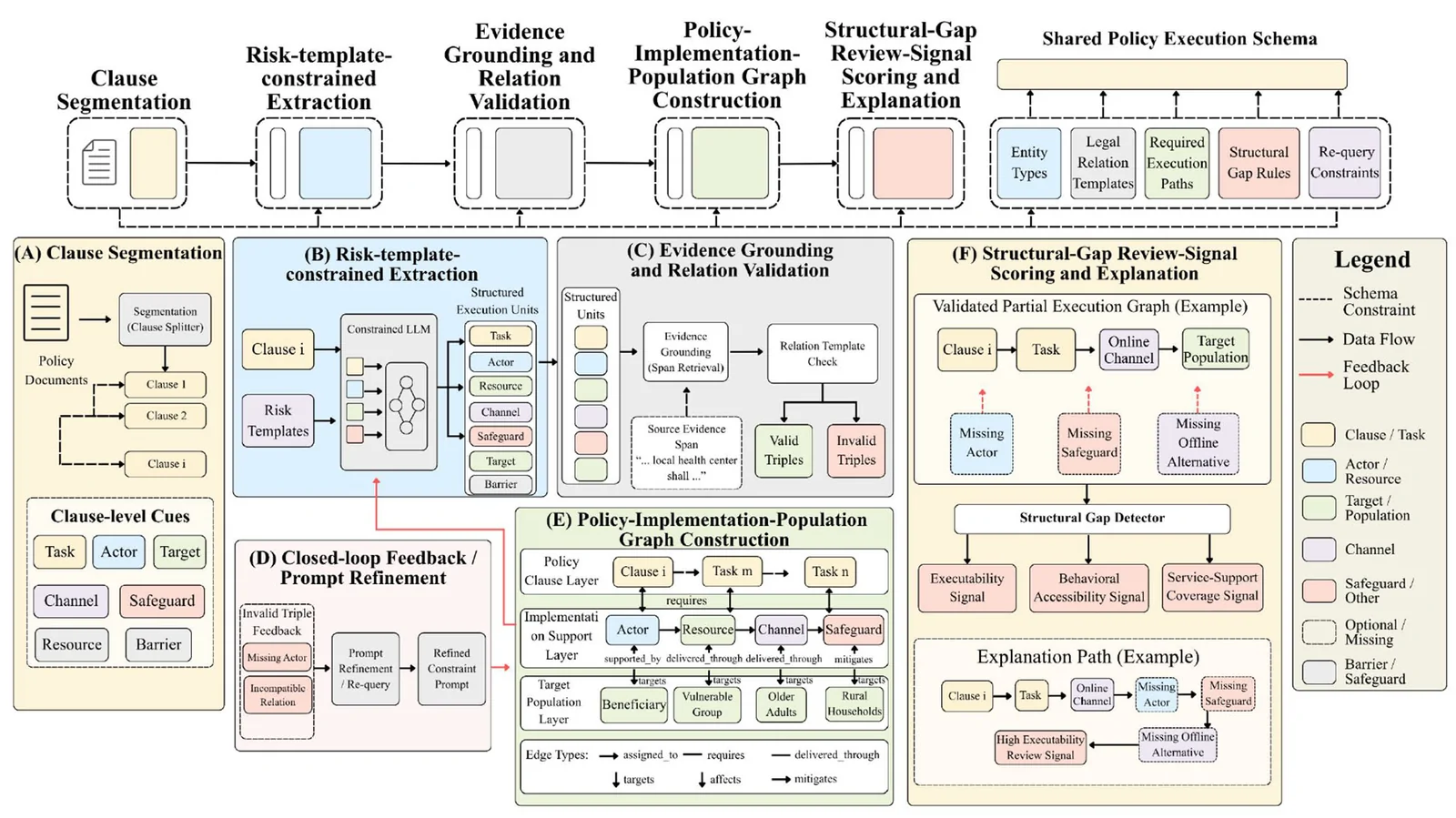
Overall framework of PRP-KG. The framework couples clause segmentation, constrained extraction, evidence grounding, graph construction, and gap-triggered structural-gap review-signal scoring via a shared policy execution schema. Invalid triples are fed back to refine extraction, forming a closed-loop validation mechanism.

As shown in Figure 1, PRP-KG comprises five schema-coupled stages: clause segmentation, constrained extraction, evidence binding and graph validation, three-layer graph construction, and structural-gap scoring. The shared policy execution schema defines the entity types, relation templates, required execution paths, and consistency rules that apply across these stages.

First, each policy document is segmented into clauses. Second, a large language model extracts candidate tasks, actors, resources, channels, target populations, barriers, and safeguards using risk-template prompts. Third, the extracted entities and relations are linked to source evidence spans and validated against the schema. Fourth, the validated elements are organized into a three-layer Policy–Implementation–Population graph. Finally, missing or incomplete execution and mitigation paths are mapped to the three review-signal dimensions, along with their supporting explanation paths.

Graph validation also provides clause-level feedback. When a candidate triple violates a type constraint or leaves a required relation unresolved, the violated constraint is incorporated into a revised prompt, and the clause is reprocessed. For example, the model may be re-queried when a task lacks a responsible actor or when a target population is connected through an incompatible relation. Graph consistency therefore serves as an iterative extraction constraint rather than solely as a post-processing filter.

### 3.3 Risk-template-constrained policy element extraction

**Figure 2:**
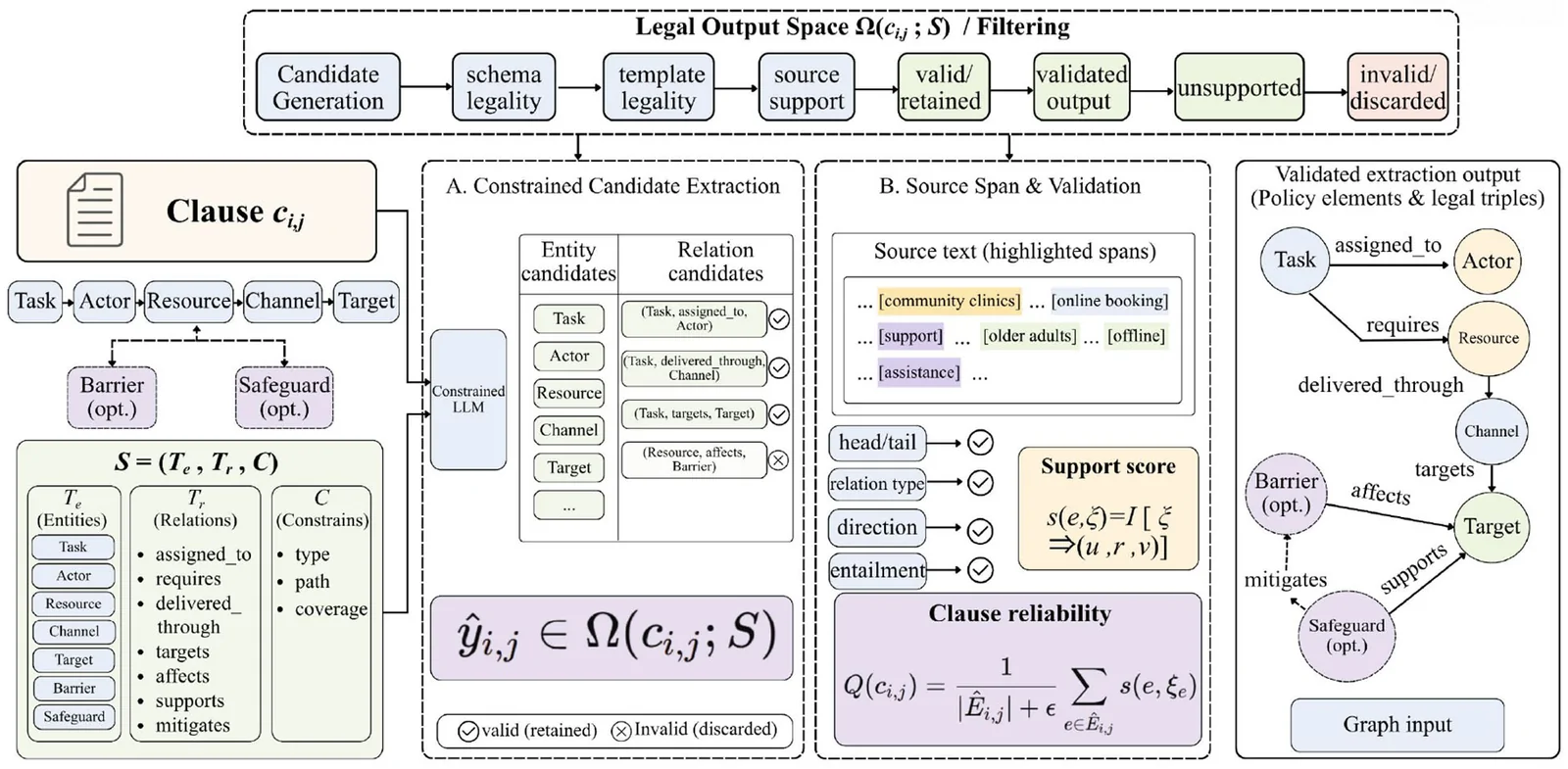
Risk-template-constrained policy element extraction.

Direct LLM extraction may yield fluent but unsupported, incomplete, or schema-incompatible policy elements. As shown in Figure 2, PRP-KG uses a predefined schema as a prompting constraint and a condition for valid output, rather than as a trainable extraction objective. The schema is defined as Equation 5


$$\begin{array}{l} S=\left(T_{e},T_{r},C\right), \end{array}$$ (5)

Where *T*_*e*_ is the entity type set, *T*_*r*_ is the relation type set, and *C* is the consistency rule set. The entity types are shown as Equation 6


$$\begin{array}{l} T_{e}=\left\{\text{Task},\text{Actor},\text{Resource},\text{Channel},\text{Target},\text{Barrier},\text{Safeguard}\right\}. \end{array}$$ (6)

The relation templates include assigned_to, requires, delivered_through, targets, affects, supports, and mitigates. These templates specify legal links among policy execution elements and constrain LLM extraction.

For each clause *c*_*i, j*_, the LLM output ŷ_*i, j*_ must satisfy the legal output condition as Equation 7


$$\begin{array}{l} {ŷ}_{i,j}\in \Omega \left(c_{i,j};S\right), \end{array}$$ (7)

Where Ω(*c*_*i, j*_; *S*) denotes the set of schema-valid outputs for the clause. This condition restricts the output to predefined entity types, relation templates, and evidence-linked triples. It is a decoding and validation constraint, not a supervised training loss.

Each extracted entity and relation must be linked to a source evidence span. At the relationship level, the evidence primarily focuses on verifying the relationship triple *e* = (*u, r, v*). The corresponding evidence fragment needs to simultaneously support the head entity, tail entity, relationship type, and its relationship direction. Mentioning only two entities without providing relationship information is not valid evidence. The support score is defined as Equation 8


$$\begin{array}{l} s\left(e,\xi \right)=𝕀\left[\xi \Rightarrow \left(u,r,v\right)\right], \end{array}$$ (8)

Where ξ is the evidence span and ξ⇒(*u, r, v*) means that the span entails the complete relation claim. If the span is neutral, only shows entity co-occurrence, contradicts the relation, or supports the opposite direction, the score is 0.

The clause-level extraction reliability is shown as Equation 9


$$\begin{array}{l} Q\left(c_{i,j}\right)=\frac{1}{\left|{Ê}_{i,j}\right|+\epsilon }\underset{e\in {Ê}_{i,j}}{\sum }s\left(e,\xi_{e}\right), \end{array}$$ (9)

Where Ê_*i, j*_ is the set of extracted relations from clause *c*_*i, j*_, ξ_*e*_ is the evidence span linked to relation *e*, and ϵ avoids division by zero. This score measures relation-level evidence sufficiency under the schema. It does not claim full semantic correctness on its own; semantic correctness is further evaluated through expert annotations in the experiments.

The proposed module constrains LLM-based policy element extraction using the schema *S* = (*T*_*e*_, *T*_*r*_, *C*), risk template *Q*, legal output space Ω(*S*), and source-span validation, producing validated policy elements and legal triples for graph construction.

### 3.4 Policy–implementation–population graph construction

The validated extraction outputs are organized into a three-layer knowledge graph as Equation 10:


$$\begin{array}{l} G_{i}=\left(V_{i}^{p},V_{i}^{s},V_{i}^{g},E_{i}\right), \end{array}$$ (10)

**Figure 3:**
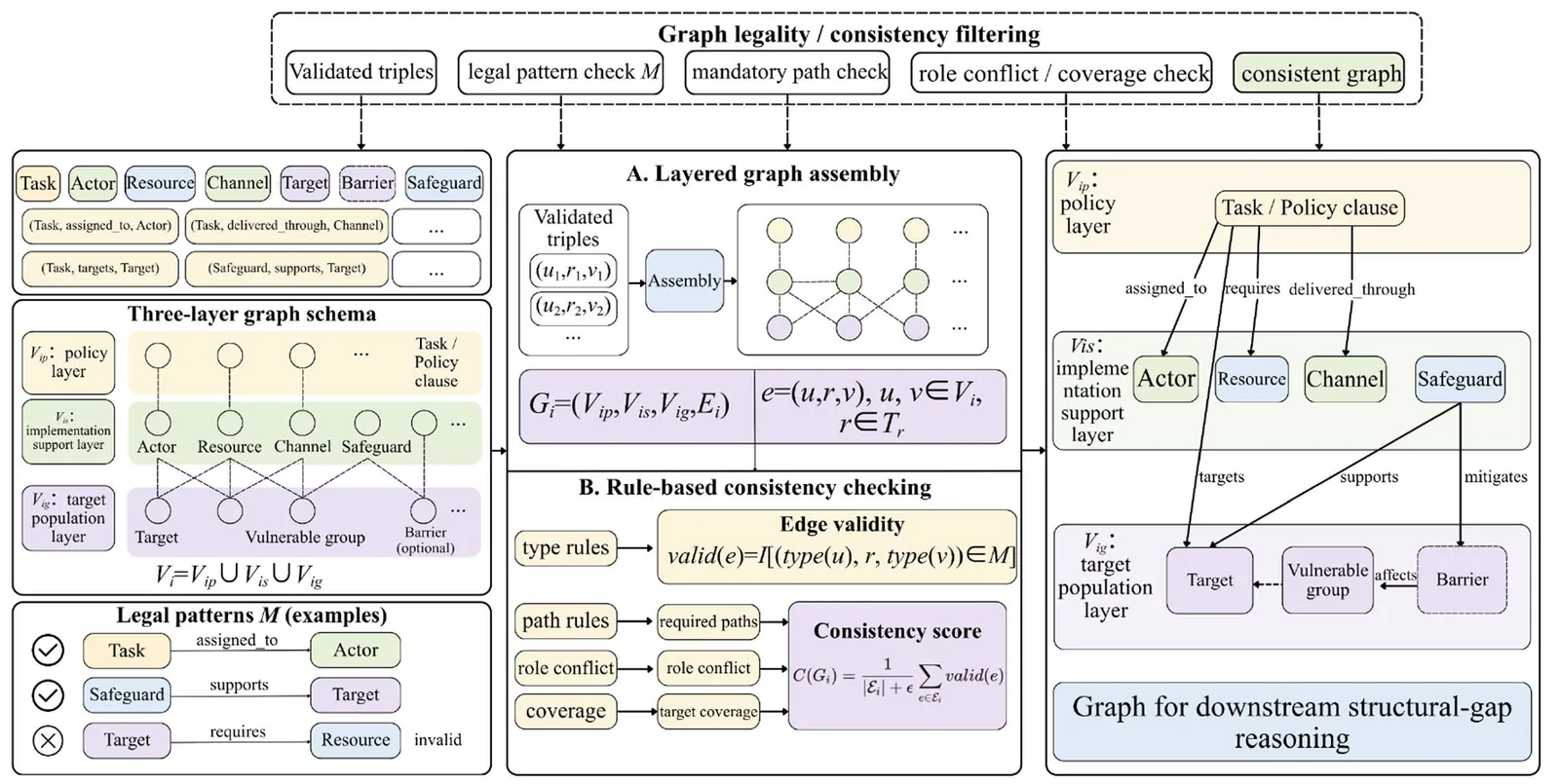
Three-layer graph construction and rule-based validation. Edge validity and structural-gap checks are evaluated separately.

Figure 3 illustrates how validated policy elements and legal triples are organized into a three-layer graph and filtered by rule-based consistency checks before downstream risk reasoning.

Where $V_{i}^{p}$ is the policy clause layer, $V_{i}^{s}$ is the implementation support layer, and $V_{i}^{g}$ is the target population layer. The implementation support layer includes actors, resources, service channels, and safeguards. The target population layer includes intended beneficiaries and vulnerable groups.

Each edge is represented as a typed triple as Equation 11:


$$\begin{array}{l} e=\left(u,r,v\right),u,v\in V_{i},\;r\in T_{r}. \end{array}$$ (11)

Let M denote the set of legal type–relation–type patterns. For example, (*Task, assigned*_*to, Actor*) and (*Safeguard, supports, Target*) are valid, while (*Target, requires, Resource*) is not valid unless the schema explicitly allows it. Edge validity is defined as Equation 12


$$\begin{array}{l} valid\left(e\right)=𝕀[\left(type\left(u\right),r,type\left(v\right)\right)\in M]. \end{array}$$ (12)

The edge-validity ratio is defined as


$$\begin{array}{l} C_{\text{edge}}\left(G_{i}\right)=\frac{1}{\left|E_{i}\right|+\epsilon }\underset{e\in E_{i}}{\sum }\text{valid}\left(e\right). \end{array}$$ (13)

This ratio measures only the proportion of extracted edges that satisfy the legal type–relation–type patterns in M. The broader validation protocol also includes mandatory-path, role-conflict, and coverage checks. These checks are evaluated separately and are not included in Equation 13. Mandatory-path and coverage failures are retained as structural-gap indicators, whereas role conflicts are recorded as separate validation flags.

The rule set C contains four types of checks: type rules, mandatory-path rules, role-conflict rules, and coverage rules. Type-rule compliance is summarized by *C*_*edge*_(*G*_*i*_) in Equation 13. The remaining three checks are applied separately to identify missing required relations, incompatible role assignments, and uncovered target-group requirements.

### 3.5 Structural-gap-driven review-signal scoring

**Figure 4:**
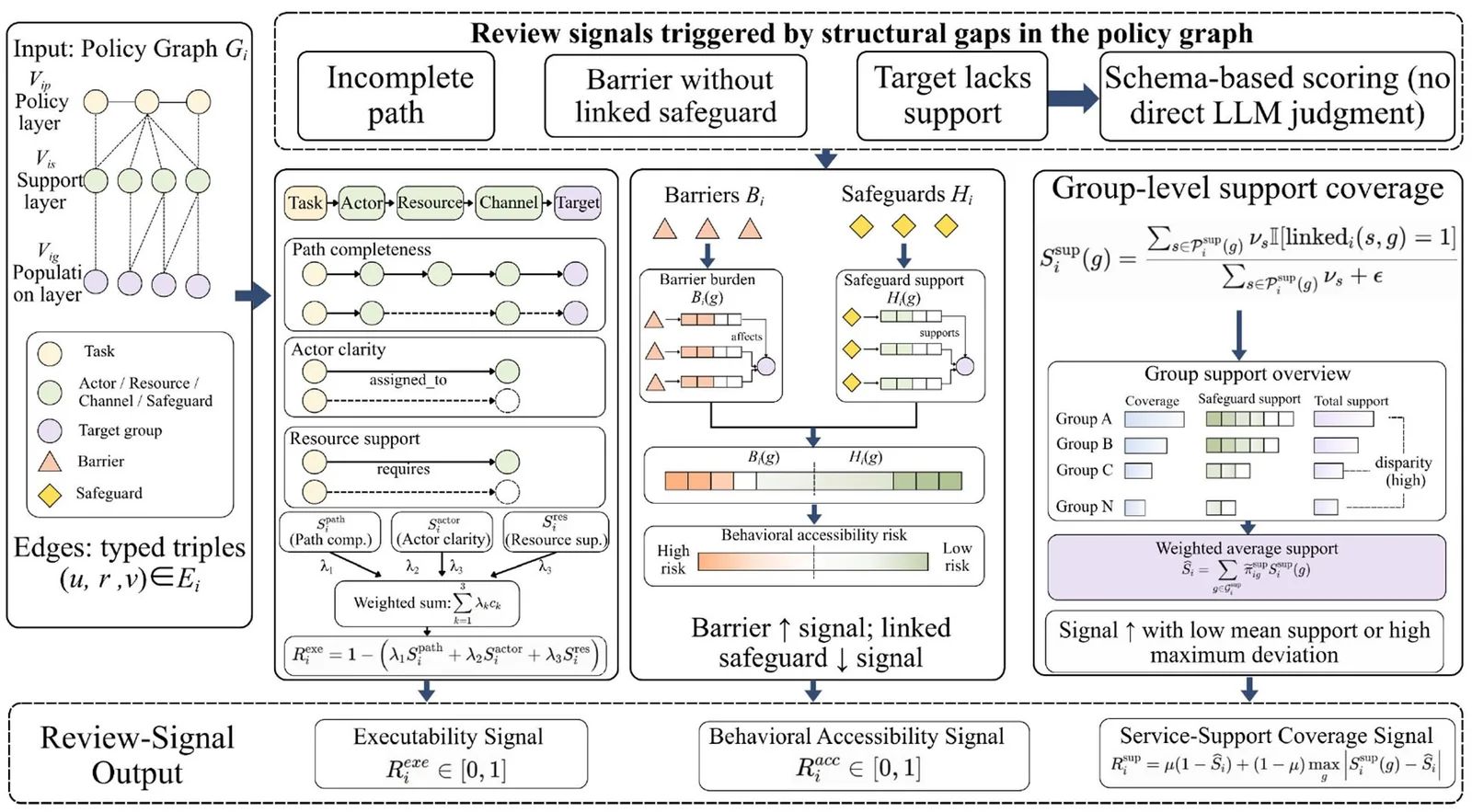
Structural-gap-driven review-signal scoring.

As shown in Figure 4, the review signals are computed from structural gaps in **G**_**i**_, rather than from direct LLM judgments. The three-layer graph provides a constrained reasoning structure in which policy clauses define tasks, the implementation layer represents actors, resources, channels, and safeguards, and the target layer represents affected groups. The resulting signals quantify text-supported structural gaps for expert-review prioritization; they do not measure realized policy implementation, accessibility, service provision, or equity outcomes. To determine whether the observed gains stem from explicit structural constraints rather than from additional graph information, the experiments compare PRP-KG with same-node and same-edge variants that omit the layer constraints.

#### 3.5.1 Policy executability signal

Let *T*_*i*_ denote the set of policy tasks extracted from clause *i*. For each task *t*∈*T*_*i*_, the policy-execution schema specifies a task-centered set of required typed edges as Equation 14:


$${ℰ}_{i}^{\text{req}}=\cup_{t\in T_{i}}\left(\begin{array}{ll} \; & \left\{\left(t,\text{assigned}\_to,a\right):a\in A_{t}^{\text{req}}\right\}\\ \cup & \left\{\left(t,\text{requires},r\right):r\in {ℛ}_{t}^{\text{req}}\right\}\\ \cup & \left\{\left(t,\text{delivered}\_through,c\right):c\in C_{t}^{\text{req}}\right\}\\ \cup & \left\{\left(t,\text{targets},g\right):g\in G_{t}^{\text{req}}\right\} \end{array}\right),$$ (14)

Where $A_{t}^{\text{req}}$, $R_{t}^{\text{req}}$, $C_{t}^{\text{req}}$, and $G_{t}^{\text{req}}$ denote the required Actor, Resource, Channel, and Target nodes for task *t*, respectively. All required relations originate directly from the Task node; no Actor–Resource, Resource–Channel, or Channel–Target edges are assumed.

Let $E_{i}$ denote the schema-valid typed edges extracted from clause *i*. The execution-structure completeness score is shown as Equation 15


$$\begin{array}{l} S_{i}^{\text{path}}=\frac{\sum_{e\in E_{i}^{\text{req}}}w_{e}𝕀\left[e\in E_{i}\right]}{\sum_{e\in E_{i}^{\text{req}}}w_{e}+\varepsilon }, \end{array}$$ (15)

Where *w*_*e*_ ≥ 0 is the prespecified importance weight for the required edge *e*, with equal weights used by default. Although the notation $S_{i}^{\text{path}}$ is retained for consistency, it measures coverage of the task-centered typed-edge set rather than completeness of a sequential chain.

Actor clarity is calculated as


$$\begin{array}{l} S_{i}^{\text{actor}}=\frac{1}{|T_{i}|+\varepsilon }\underset{t\in T_{i}}{\sum }𝕀\left[\exists a:\left(t,\text{assigned\_}to,a\right)\in E_{i}\right], \end{array}$$ (16)

and resource support is calculated as


$$\begin{array}{l} S_{i}^{\text{res}}=\frac{1}{|T_{i}|+\varepsilon }\underset{t\in T_{i}}{\sum }𝕀\left[\exists r:\left(t,\text{requires},r\right)\in E_{i}\right]. \end{array}$$ (17)

The policy executability review signal is then defined as


$$\begin{array}{c} R_{i}^{\text{exe}}=1-\left(\lambda_{1}S_{i}^{\text{path}}+\lambda_{2}S_{i}^{\text{actor}}+\lambda_{3}S_{i}^{\text{res}}\right),\;\lambda_{k}\geq 0,\\ \overset{3}{\underset{k=1}{\sum }}\lambda_{k}=1. \end{array}$$ (18)

A larger $R_{i}^{exe}$ indicates higher review priority because one or more required task-centered relations are missing, such as a responsible actor, supporting resource, delivery channel, or target.

#### 3.5.2 Behavioral accessibility

Let $B_{i}\left(g\right)$ denote the set of prespecified barrier categories applicable to the target group *g*, including online-only access requirements, device dependence, identity-authentication burden, complex platform operation, and digital-literacy requirements. The normalized barrier score is shown as Equation 19


$$\begin{array}{l} B_{i}\left(g\right)=\frac{\sum_{b\in B_{i}\left(g\right)}\omega_{b}𝕀\left[\left(b,\text{affects},g\right)\in E_{i}\right]}{\sum_{b\in B_{i}\left(g\right)}\omega_{b}+\epsilon }, \end{array}$$ (19)

Where ω_*b*_ ≥ 0 is the prespecified weight of barrier category *b*. A barrier category contributes only when it is supported by a schema-valid Barrier affects Target relation linked to group *g*. Repeated mentions of the same category are counted once.

Let $H_{i}^{acc}\left(g\right)$ denote the set of accessibility-safeguard categories applicable to group *g*, such as offline service windows, assisted-access mechanisms, telephone channels, community assistance, and proxy-application arrangements. The normalized accessibility-safeguard score is shown as Equation 20


$$\begin{array}{l} H_{i}^{acc}\left(g\right)=\frac{\begin{array}{c} \sum_{h\in H_{i}^{acc}\left(g\right)}\omega_{h}𝕀[\left(h,\text{supports},g\right)\in E_{i}\;\\ \vee \;\exists b\in B_{i}\left(g\right):\left(h,\text{mitigates},b\right)\in E_{i}] \end{array}}{\sum_{h\in H_{i}^{acc}\left(g\right)}\omega_{h}+\epsilon }. \end{array}$$ (20)

Thus, $B_{i}\left(g\right),H_{i}^{\text{acc}}\left(g\right)\in \left[0,1\right]$. The group-level access-barrier signal is defined as Equation 21


$$\begin{array}{l} r_{i}^{\text{acc}}\left(g\right)=B_{i}\left(g\right)\left[1-H_{i}^{\text{acc}}\left(g\right)\right]. \end{array}$$

Let


$$\begin{array}{l} {\tilde{\pi }}_{ig}=\frac{\pi_{g}}{\sum_{g^{\prime }\in G_{i}^{\text{acc}}}\pi_{g^{\prime }}+\epsilon ,} \end{array}$$ (21)

Where $G_{i}^{\text{acc}}$ contains the applicable target groups and π_*g*_ ≥ 0 is the prespecified group weight. The document-level signal is shown as Equation 22


$$\begin{array}{l} R_{i}^{\text{acc}}=\underset{g\in G_{i}^{\text{acc}}}{\sum }{\tilde{\pi }}_{ig}r_{i}^{acc}\left(g\right). \end{array}$$ (22)

The multiplicative formulation produces a behavioral-accessibility signal only when a text-supported barrier is linked to the target group. A safeguard reduces the signal only when a schema-valid relation connects it to the same group or an identified barrier.

#### 3.5.3 Text-implied service-support coverage review signal

Let $P_{i}^{\sup}\left(g\right)$ denote the prespecified service-support categories applicable to the target group *g*, including dedicated service channels, implementation resources, assisted-service arrangements, and other target-linked provisions defined by the fixed schema. The normalized coverage score is shown as Equation 23


$$\begin{array}{l} S_{i}^{\sup}\left(g\right)=\frac{\sum_{s\in P_{i}^{\sup}\left(g\right)}\nu_{s}𝕀\left[{\text{linked}}_{i}\left(s,g\right)=1\right]}{\sum_{s\in P_{i}^{\sup}\left(g\right)}\nu_{s}+\epsilon }, \end{array}$$ (23)

Where ν_*s*_ ≥ 0 is the prespecified weight of support category *s*. The indicator equals one only when *s*is connected to *g* through a schema-valid target–support structure. Eligible structures include


$$\begin{array}{l} \text{Task}\overset{\text{targets}}{\to }\text{Target}, \end{array}$$

together with an applicable relation such as


$$\begin{array}{l} \text{Safeguard}\overset{\text{supports}}{\to }\text{Target}, \end{array}$$

or a Task–Resource/Channel structure linked to the same target. All eligible structures are fixed before test-set evaluation.

The weighted mean support is shown as Equation 24


$$\begin{array}{l} {\hat{S}}_{i}=\sum_{g\in G_{i}^{\sup}}{\tilde{\pi }}_{ig}^{\sup}S_{i}^{\sup}\left(g\right),\;{\tilde{\pi }}_{ig}^{\sup}=\frac{\pi_{g}}{\sum_{g^{\prime }\in G_{i}^{\sup}}\pi_{g^{\prime }}+\epsilon \;}.\; \end{array}$$ (24)

The service-support-coverage review signal is shown as Equation 25


$$\begin{array}{c} R_{i}^{\sup}=\mu \left(1-{Ŝ}_{i}\right)+\left(1-\mu \right)\max_{g\in G_{i}^{\sup}}|S_{i}^{\sup}\left(g\right)\\ -{Ŝ}_{i}|,\;\mu \in \left[0,1\right]. \end{array}$$ (25)

The first term captures overall support insufficiency, whereas the second captures the largest group-level deviation from the weighted mean. Because both components lie in [0, 1], $R_{i}^{\sup}\in \left[0,1\right]$. Document–group pairs with no applicable support category are recorded as not applicable and excluded from aggregation rather than assigned a zero score.

#### 3.5.4 Ordinal review-signal mapping

PRP-KG outputs the three continuous review signals separately:


$$\begin{array}{l} R_{i}^{\text{exe}},\;R_{i}^{\text{acc}},\;R_{i}^{\sup}\in \left[0,1\right].\; \end{array}$$

No cross-dimensional weighted sum or combined review-signal score is constructed. For each review dimension *d* ∈ {*exe, acc*, sup}, four validation-selected cutpoints map the continuous signal *R*_*i, d*_ to the five ordinal categories used in five-category Macro-F1 evaluation:


$${\hat{y}}_{i,d}=\{\begin{array}{cc} 0, & R_{i,d}<c_{d,1},\\ 1, & c_{d,1}\leq R_{i,d}<c_{d,2},\\ 2, & c_{d,2}\leq R_{i,d}<c_{d,3},\\ 3, & c_{d,3}\leq R_{i,d}<c_{d,4},\\ 4, & R_{i,d}\geq c_{d,4}, \end{array}$$ (26)

Where


$$\begin{array}{l} c_{d,1}<c_{d,2}<c_{d,3}<c_{d,4}. \end{array}$$

The four cutpoints for each dimension were selected exclusively from the DHP-FedReg-HHS validation split as part of the prespecified discrete configuration search. Five-category Macro-F1 was the sole validation-selection criterion. The selected cutpoints were fixed before evaluation on the DHP-FedReg-HHS test split and the DHP-MultiEURLEX-Health and DHP-BillSum-Health cross-dataset test sets. Their numerical values and candidate sets are reported in Supplementary Appendix B11.

The resulting labels take values in {0, 1, 2, 3, 4}, with higher values indicating greater review priority under the predefined structural-gap rubric. Labels 3 and 4 form the positive class only for the complementary high-priority F1 metric and remain separate in the five-category Macro-F1 evaluation. “Not applicable” denotes eligibility status rather than a sixth ordinal category.

#### 3.5.5 Worked scoring example

Consider a policy graph with two extracted tasks, *t*_1_ and *t*_2_. For illustration, each task requires four task-centered typed relations: an assigned actor, a supporting resource, a delivery channel, and a target group. With equal required-edge weights, the required edge set is


$$\begin{array}{l} E_{i}^{\text{req}}\;=\{\left(t_{1},\text{assigned\_}to,a_{1}\right),\left(t_{1},\text{requires},r_{1}\right),\\ \;\left(t_{1},\text{delivered\_through},c_{1}\right),\left(t_{1},\text{targets},g_{1}\right),\\ \;\left(t_{2},\text{assigned\_}to,a_{2}\right),\left(t_{2},\text{requires},r_{2}\right),\\ \;\left(t_{2},\text{delivered\_through},c_{2}\right),\left(t_{2},\text{targets},g_{2}\right)\}. \end{array}$$

Suppose that all four required relations for *t*_1_ are recovered, whereas *t*_2_ has a linked resource, delivery channel, and target group but no explicitly assigned actor. The recovered required-edge set is therefore


$$\begin{array}{l} E_{i}\cap E_{i}^{req}=\{\left(t_{1},\text{assigned\_}to,a_{1}\right),\left(t_{1},\text{requires},r_{1}\right),\\ \;\left(t_{1},\text{delivered\_through},c_{1}\right),\left(t_{1},\text{targets},g_{1}\right),\\ \;\left(t_{2},\text{requires},r_{2}\right),\left(t_{2},\text{delivered\_through},c_{2}\right),\\ \;\left(t_{2},\text{targets},g_{2}\right)\}. \end{array}$$

Thus, seven of the eight equally weighted required edges are recovered. Omitting the negligible ϵterm from the numerical display because the denominator is nonzero,


$$\begin{array}{l} S_{i}^{\text{path}}=\frac{7}{8}=0.875. \end{array}$$

One of the two tasks has an explicitly assigned actor, while both tasks have a linked resource. Therefore,


$$\begin{array}{l} S_{i}^{\text{actor}}=\frac{1}{2}=0.50,\;S_{i}^{\text{res}}=\frac{2}{2}=1.00. \end{array}$$

Using the validation-selected executability-component weights


$$\begin{array}{l} \left(\lambda_{1},\lambda_{2},\lambda_{3}\right)=\left(0.50,0.25,0.25\right), \end{array}$$

the executability review signal is shown as Equation 27


$$\begin{array}{l} R_{i}^{\text{exe}}=1-\left(0.50\times 0.875+0.25\times 0.50+0.25\times 1.00\right)\\ \;=1-\left(0.4375+0.125+0.250\right)\\ \;=0.1875. \end{array}$$ (27)

The actor and resource components are reported separately even though their corresponding relations also contribute to required-edge coverage, because Equations 16–18 explicitly assign additional importance to actor clarity and resource support. These weights apply only within the executability dimension and do not combine the three review dimensions.

For behavioral accessibility, suppose that two barrier categories apply to older adults and that one evidence-linked barrier—identity-authentication burden—is connected to this group via a valid affects relation. Suppose further that none of the four applicable safeguard categories is connected to the same group or barrier. Assuming equal category weights and that older adults are the only applicable group,


$$\begin{array}{l} B_{i}\left(g\right)=\frac{1}{2}=0.50,\;H_{i}^{acc}\left(g\right)=\frac{0}{4}=0. \end{array}$$

The behavioral-accessibility review signal is therefore shown as Equation 28


$$\begin{array}{l} R_{i}^{\text{acc}}=0.50\left(1-0\right)=0.500. \end{array}$$ (28)

For service-support coverage, assume that older adults and general adult users have group-level support scores of 0.25 and 0.75, respectively, with equal normalized group weights. The weighted mean support is


$$\begin{array}{l} {Ŝ}_{i}=0.50\times 0.25+0.50\times 0.75=0.50, \end{array}$$

and the maximum group-level deviation from the weighted mean is shown as Equation 29 and


$$\begin{array}{l} \underset{g}{\max}|S_{i}^{\sup}\left(g\right)-{Ŝ}_{i}|=\max\left(|,0.25-0.50∥0.75-0.50|\right)=0.25. \end{array}$$

With μ = 0.70,


$$\begin{array}{l} R_{i}^{\sup}=0.70\left(1-0.50\right)+0.30\left(0.25\right)\\ \;=0.350+0.075\\ \;=0.425. \end{array}$$ (29)

The resulting continuous review signals are shown as Equation 30


$$\begin{array}{l} \left(R_{i}^{exe},R_{i}^{acc},R_{i}^{\sup}\right)=\left(0.1875,\;0.500,\;0.425\right). \end{array}$$ (30)

Each signal is mapped independently to an ordinal label using the dimension-specific cutpoints in Equation 26. Because the selected cutpoints are reported in Supplementary Appendix B11, only the continuous signals are shown here.

Incomplete paths, unmitigated barriers, and weak target-group support are mapped to three text-implied structural-gap review signals.

### 3.6 Weight calibration and explanation generation

The coefficients λ_*k*_ weight the executability components; ω_*b*_, ω_*h*_, and ν_*s*_ weight the predefined barrier, safeguard, and support-path categories, respectively; π_*g*_ weights target-group types; and μ balances low weighted-mean support against maximum group-level deviation. Accordingly, *B*_*i*_(*g*) is a normalized barrier score weighted by ω_*b*_, rather than a binary indicator. No separate global barrier coefficient η_*b*_ is used.

Empirically calibrated parameters are selected only on the DHP-FedReg-HHS validation split using five-category Macro-F1. Rubric-defined weights are prespecified and examined through sensitivity analysis. All weights and ordinal cutpoints are frozen before test and cross-dataset evaluation. MAE, Spearman's ρ, and quadratic weighted kappa are reported only as complementary metrics.

PRP-KG generates evidence-linked review-flag subgraphs that highlight incomplete execution relations, barrier–target links without connected safeguards, and weak or absent target-group support. Extracted components are linked to source spans, whereas missing links are inferred from the predefined schema. These outputs serve as textual screening signals and do not establish implementation, accessibility, service-provision, or equity outcomes.

### 3.7 Design properties and complexity

PRP-KG has three design properties. First, schema-constrained extraction reduces structurally invalid outputs but does not guarantee semantic correctness or complete evidence recovery. Second, graph validation detects illegal triples and independently verifies mandatory relations, role conflicts, and coverage gaps. Re-query outputs are retained only after relation-level evidence validation. Third, with fixed weights and cutpoints, executability is monotonic with respect to validated additions that complete required execution relations. For a fixed barrier set, adding an applicable safeguard link cannot increase the behavioral-accessibility signal, whereas adding a barrier link may increase it. No unconditional monotonicity claim is made for service-support coverage because a new group-specific support link may alter the maximum-deviation term.

Let *M*_*i*_ be the number of clauses, *K* the maximum candidate elements per clause, $|E_{i}|$ the number of graph edges, and $|P_{i}^{req}|$ the number of required execution structures. Excluding LLM inference, extraction validation requires *O*(*M*_*i*_*K*) time, graph checking requires $O\left(|E_{i}|\right)$, and review-signal scoring requires $O\left(|P_{i}^{req}|+|E_{i}|\right)$. LLM cost depends on input length, output length, and re-query rounds.

### 3.8 Algorithmic workflow

Algorithm 1 summarizes PRP-KG. The workflow separates LLM extraction from symbolic validation and graph scoring. If re-query fails to produce relation-level evidence, the missing item is left as a structural gap.

Algorithm 1 PRP-KG-based text-implied policy-text review-signal screening. 
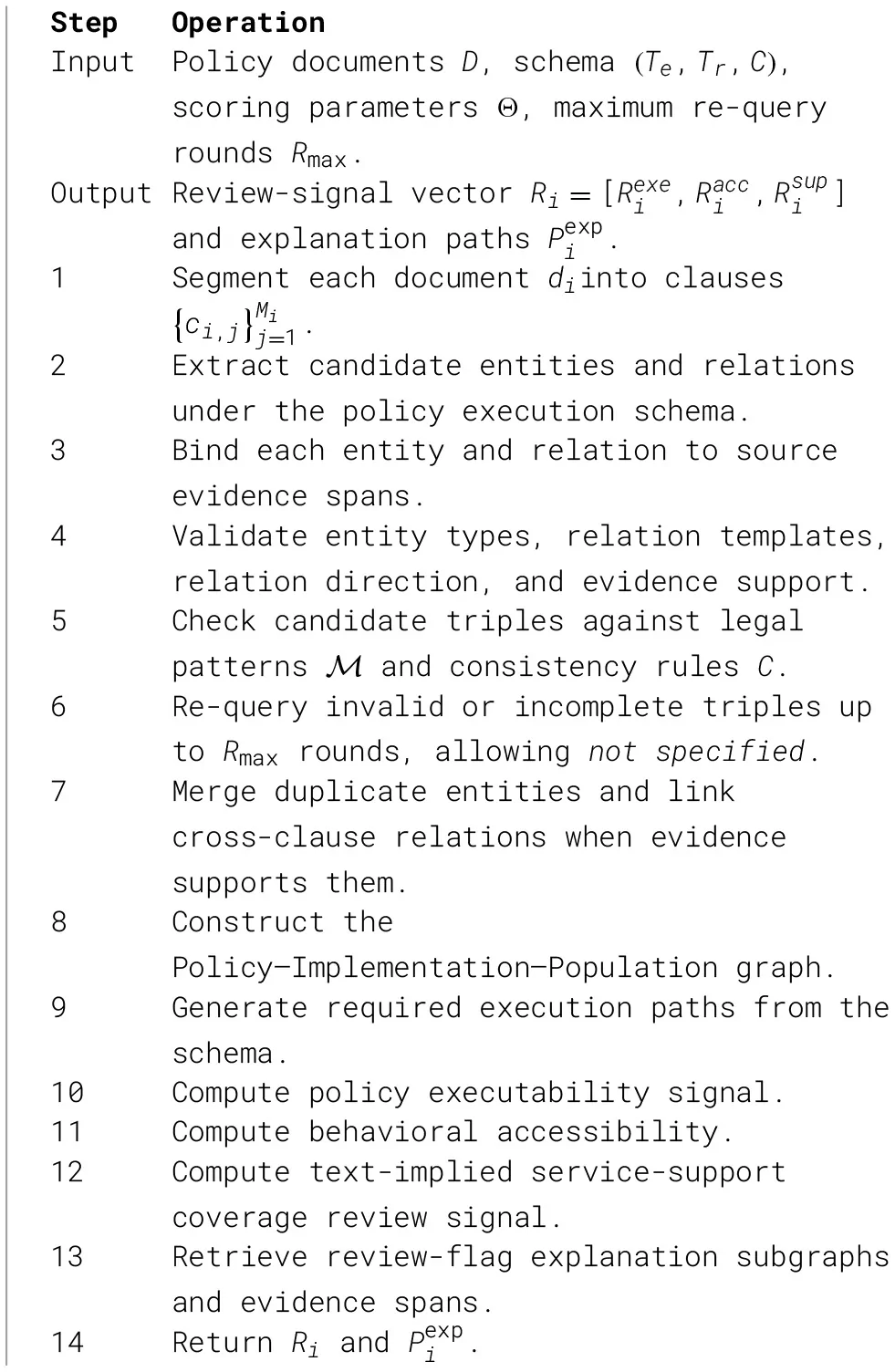

The output is not an opaque policy score. Each score is linked to graph paths, structural gaps, and source evidence spans for expert inspection.

Cost is reported separately for LLM calls and symbolic computation. For document *d*_*i*_, let *M*_*i*_ be the number of clauses, *K* the maximum number of extracted elements per clause, $|E_{i}|$ the number of graph edges, and $|P_{i}^{req}|$ the number of required paths. Schema validation costs *O*(*M*_*i*_*K*), graph checking costs $O\left(|E_{i}|\right)$, and scoring costs $O\left(|P_{i}^{req}|+|E_{i}|\right)$. With at most *R*_max_ re-query rounds, the worst-case LLM calls per document are *M*_*i*_(1+*R*_max_). Token, memory, and index construction costs are reported in the efficiency experiment.

## 4 Experiments and results

This section evaluates PRP-KG on policy element extraction, graph construction, three-dimensional text-implied review-signal prediction, ablation, robustness, and explanation quality. Results are reported as mean ± standard deviation over five seeds. Statistical significance is assessed using paired bootstrap resampling with Holm's step-down procedure. Experiments were conducted on a workstation equipped with an NVIDIA 5090D V2 GPU (NVIDIA Corporation, Santa Clara, CA, USA), 128 GB RAM (Shenzhen Jiaheshengwei Electronics Technology Co., Ltd., Shenzhen, Guangdong, China), running Ubuntu 22.04 (Canonical Group Limited, London, UK), Python 3.10 (Python Software Foundation, Beaverton, OR, USA), PyTorch 2.7.1 (PyTorch Foundation/The Linux Foundation, Wilmington, DE, USA), and CUDA 12.8 (NVIDIA Corporation, Santa Clara, CA, USA).

### 4.1 Datasets and annotation protocol

#### 4.1.1 Data sources and corpus construction

We constructed three quantitative corpora from publicly accessible legislative and policy sources. DHP-FedReg-HHS was compiled from the Federal Register and GovInfo using prespecified agency, topic, and keyword filters covering public health, digital health, Medicare, Medicaid, telehealth, patient access, interoperability, and health-service delivery. DHP-MultiEURLEX-Health and DHP-BillSum-Health were derived from health- and digital-service-related documents in MultiEURLEX and BillSum, respectively.

DHP-FedReg-HHS was used for model development and in-domain evaluation. DHP-MultiEURLEX-Health and DHP-BillSum-Health were used only for cross-dataset evaluation, without corpus-specific prompt revision, coefficient or threshold selection, or hyperparameter tuning. Because all three corpora were annotated under the same task definition, schema, and annotation guidelines, these experiments assess transfer across document sources and policy genres but do not constitute fully independent external validation.

Documents from WHO MiNDbank and LawAtlas were used solely as supplementary qualitative cases. They were excluded from model training, validation-based selection, prompt refinement, and quantitative evaluation. Source versions, collection dates, search queries, eligibility criteria, document identifiers, and corpus-reconstruction procedures are reported in Supplementary Appendix A. Table 1 summarizes the corpus statistics and evaluation roles.

**Table 1:** Dataset statistics and evaluation roles.

Dataset	Documents	Clauses	Annotated clauses	Entities in annotated subset	Relations in annotated subset	Avg. document length (tokens)	Evaluation role
DHP-FedReg-HHS	6,420	83,760	9,600	61,842	74,315	4,860	Model development and in-domain evaluation
DHP-MultiEURLEX-Health	4,280	51,920	5,200	34,671	39,804	3,970	Cross-dataset evaluation
DHP-BillSum-Health	3,160	46,280	4,800	31,458	36,277	5,640	Cross-dataset evaluation
WHO-MiNDbank-case	420	6,140	—	—	—	3,280	Supplementary qualitative cases only
LawAtlas-policy-case	310	3,880	—	—	—	2,740	Supplementary qualitative cases only

#### 4.1.2 Document filtering, deduplication, and splitting

Documents were selected based on prespecified criteria for source, agency, topic, and keyword. Near-duplicate detection followed a three-stage deduplication and cross-source document-linking procedure. Candidate document pairs were first retrieved using MinHash-estimated Jaccard similarity with a threshold of 0.85. Pairs with an estimated similarity of at least 0.90 were prioritized for manual review. Final duplicate removal and document-family assignment were determined by manual review rather than by either similarity threshold alone.

All data partitions were created at the document-family level. Documents belonging to the same duplicate, amendment, or agency–topic–regulation family were assigned to the same partition. Consequently, clauses and annotations derived from a document could not appear in different partitions. DHP-FedReg-HHS was divided into training, validation, and test partitions at a document-level ratio of 70/10/20.

For DHP-MultiEURLEX-Health and DHP-BillSum-Health, the original corpus partitions were retained where available, subject to the constraint that related document families remained within a single partition. Models were trained and selected exclusively on DHP-FedReg-HHS and then applied directly to the two cross-dataset test sets. Labels from these test sets were not used for model selection, hyperparameter tuning, coefficient or cutpoint selection, prompt refinement, or error-driven system revision.

#### 4.1.3 Annotation schema and ordinal review signals

Annotation was conducted at the clause–review-dimension level. Each eligible instance was annotated with policy-element spans, typed relations, supporting evidence spans, and an ordinal review-signal label:


$$\begin{array}{c} y_{c,d}\in \left\{0,1,2,3,4\right\}, \end{array}$$

Where higher values indicate higher review priority under the predefined rubric, rather than more severe observed policy outcomes. Eligibility was determined separately for executability, behavioral accessibility, and service-support coverage; non-applicable clauses were excluded from the corresponding dimension.

For document-level evaluation, reference labels and predictions were aggregated separately by review dimension using the prespecified maximum rule:


$$\begin{array}{c} y_{i,d}^{\text{doc}}=\underset{c\in C_{i}^{\left(d\right)}}{\max}y_{c,d},\;{ŷ}_{i,d}^{\text{doc}}=\underset{c\in C_{i}^{\left(d\right)}}{\max}{ŷ}_{c,d}. \end{array}$$

Documents without an eligible clause for dimension *d* were excluded from that dimension-specific analysis. Document-level MAE was calculated as


$$\begin{array}{c} {\text{MAE}}_{d}=\frac{1}{N_{d}}\underset{i\in D_{d}}{\sum }|y_{i,d}^{\text{doc}}-{ŷ}_{i,d}^{\text{doc}}|, \end{array}$$

where $D_{d}$ denotes the eligible DHP-FedReg-HHS test documents for dimension *d*, and $N_{d}=\;|D_{d}|$. After document-level aggregation, the verified sample sizes were


$$\begin{array}{c} N_{\text{exe}}=1,124,\;N_{\text{acc}}=1,037,\;N_{\sup}=982. \end{array}$$

These sample sizes are also reported in Supplementary Table A2 and Table 2. Document families were used only as clusters in the paired bootstrap analysis.

**Table 2:** Paired document-family cluster-bootstrap comparisons on the DHP-FedReg-HHS test split.

Baseline	Executability ΔMAE [95% CI]	Adj. *p*	Behavioral accessibility ΔMAE [95% CI]	Adj. *p*	Service-support coverage ΔMAE [95% CI]	Adj. *p*	Overall ΔMAE [95% CI]
Longformer	0.183 [0.121, 0.245]	0.004	0.244 [0.177, 0.310]	0.002	0.257 [0.186, 0.327]	0.003	0.228 [0.176, 0.280]
Dense-RAG	0.127 [0.067, 0.187]	0.009	0.186 [0.121, 0.251]	0.005	0.211 [0.142, 0.280]	0.006	0.175 [0.125, 0.225]
HGT	0.080 [0.011, 0.149]	0.046	0.137 [0.067, 0.207]	0.019	0.146 [0.071, 0.221]	0.023	0.121 [0.066, 0.176]
GraphRAG	0.100 [0.025, 0.175]	0.032	0.157 [0.088, 0.226]	0.012	0.185 [0.109, 0.261]	0.015	0.147 [0.090, 0.204]
LightPROF	0.065 [−0.014, 0.144]	0.112	0.121 [0.047, 0.195]	0.029	0.156 [0.077, 0.235]	0.031	0.114 [0.054, 0.174]
EPERM	0.076 [−0.009, 0.161]	0.096	0.108 [0.035, 0.181]	0.027	0.138 [0.061, 0.215]	0.028	0.107 [0.046, 0.168]
AtomR	0.051 [−0.026, 0.128]	0.164	0.115 [0.027, 0.203]	0.041	0.147 [0.051, 0.243]	0.043	0.104 [0.034, 0.174]

Clause-level reference labels and predictions were aggregated separately by document and review dimension using the maximum rule, yielding *N*_*exe*_= 1,124, *N*_*acc*_= 1,037, and *N*_sup_= 982. Document families were resampled with replacement in 10,000 paired cluster-bootstrap replicates. Within each replicate, Δ*MAE* = *MAE*_*baseline*_−*MAE*_*PRP*−*KG*_ was calculated for each seed and averaged across the five seeds; positive values favor PRP-KG. The reported CIs are percentile 95% bootstrap intervals. Dimension-specific *p*-values were Holm-adjusted across 21 comparisons. Overall effects and CIs are descriptive and unadjusted.

Before document-level aggregation, the DHP-FedReg-HHS test split contained 1,853, 1,680, and 1,593 eligible clause-level instances for executability, behavioral accessibility, and service-support coverage, respectively. DHP-MultiEURLEX-Health and DHP-BillSum-Health were evaluated separately and were not pooled with DHP-FedReg-HHS in the primary evaluation or in statistical resampling. Across the three test corpora, the corresponding descriptive clause-level totals were 3,768, 3,390, and 3,212. These totals reflect cross-corpus coverage only and are not the sample sizes used in Table 2.

**Figure 5:**
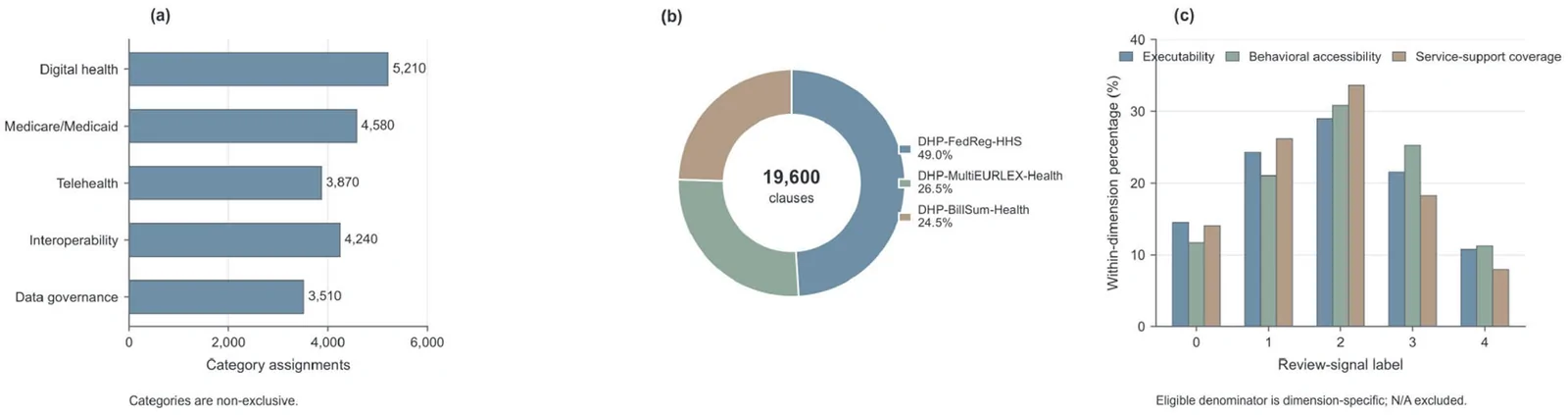
Corpus composition and clause-level review-signal distributions. **(a)** Policy-category composition. **(b)** Annotated clauses by corpus. **(c)** Clauses-level review-signal distribution. Categories in **(a)** are non-exclusive; percentages in **(c)** are calculated within each eligible dimension.

The labels represent text-implied review priorities and do not measure realized implementation failure, behavioral inaccessibility, service inequity, or population-level outcomes. Detailed clause- and document-level eligibility statistics are provided in Supplementary Table A2. The overall corpus composition, annotated-clause distribution across the three corpora, and clause-level review-signal distributions are summarized in Figure 5.

#### 4.1.4 Annotator training, agreement, and adjudication

Three annotators participated: two with postgraduate training in public health and one with experience in health-policy analysis. They first independently annotated a 180-clause pilot set—60 clauses per corpus—using a standardized guideline. Disagreements were reviewed with a senior public-health policy researcher, and the refined rubric was used for formal annotation. Pilot clauses were excluded from evaluation.

As shown in Table 3 each of the 19,600 formal clauses was initially assigned to one annotator. A stratified 10% subset—960 FedReg, 520 MultiEURLEX, and 480 BillSum clauses—was independently annotated by all three annotators. Mean pairwise F1 measured agreement across entity, relation, and evidence annotations; Krippendorff's ordinal α measured agreement on the three clause-level review signals. All statistics were calculated before adjudication and clause-to-document aggregation.

**Table 3:** Pre-adjudication agreement on the 1,960-clause overlap subset.

Annotation layer	Metric	FedReg (960)	MultiEURLEX (520)	BillSum (480)
Entity spans and types	Mean pairwise F1	0.858	0.832	0.819
Typed relations	Mean pairwise F1	0.811	0.785	0.768
Evidence spans	Mean pairwise token-overlap F1	0.836	0.805	0.789
Executability	Ordinal α	0.779	0.748	0.731
Behavioral accessibility	Ordinal α	0.752	0.721	0.704
Service-support coverage	Ordinal α	0.735	0.707	0.689

F1 is averaged across the three annotator pairs; ordinal α is calculated jointly across all three annotators. All statistics use the original clause-level annotations before adjudication and aggregation and therefore do not measure agreement on document-level scores.

Overlapping disagreements were adjudicated using the finalized rubric and source evidence. The remaining clauses retained their single-annotator labels unless selected for the quality-control procedure in Supplementary Appendix A. Annotators were blinded to model outputs and results. Finalized clause labels were then aggregated into document-level reference scores using the rule above.

### 4.2 Baselines, metrics, and implementation details

PRP-KG was compared with text classifiers, LLM prompting and RAG methods, graph and KG-LLM models, and information-extraction baselines (Table 4). Trainable methods used the DHP-FedReg-HHS training split. Hyperparameters and ordinal cutpoints were selected exclusively on its validation split using five-category Macro-F1 as the sole criterion and were frozen before test evaluation. DHP-MultiEURLEX-Health and DHP-BillSum-Health were evaluated separately without tuning or recalibration.

**Table 4:** Baseline methods and evaluated task coverage.

Group	Baseline	Ordinal scoring	Extraction	Graph reasoning	Evidence-linked explanation
Traditional text	TF-IDF + LinearSVC	✓	✗	✗	✗
Text encoder	BERT (34)	✓	✗	✗	✗
Text encoder	LEGAL-BERT (35)	✓	✗	✗	✗
Long-document encoder	Longformer (36)	✓	✗	✗	✗
LLM prompting	LLM-direct	✓	✗	✗	Limited
LLM prompting	LLM-CoT	✓	✗	✗	Limited
LLM prompting	LLM-self-consistency	✓	✗	✗	Limited
Retrieval	BM25-RAG	✓	✗	✗	✓
Retrieval	Dense-RAG	✓	✗	✗	✓
Evidence-aware retrieval	Text-RAG + evidence	✓	✗	✗	✓
Rule graph	Rule-KG Scoring	✓	✓	✓	✓
Graph neural model	R-GCN (37)	✓	✗	✓	✗
Graph neural model	HGT (38)	✓	✗	✓	✗
Graph-RAG	GraphRAG	✓	✗	✓	✓
KG-LLM reasoning	LightPROF (39)	✓	✗	✓	✓
Evidence-path reasoning	EPERM (40)	✓	✗	✓	✓
Heterogeneous reasoning	AtomR (41)	✓	✗	✓	✓
Information extraction	BERT-CRF	✗	✓	✗	✗
Information extraction	SpERT (42)	✗	✓	✗	✗
Information extraction	CasRel (43)	✗	✓	✗	✗
Information extraction	UIE (44)	✗	✓	✗	✗

“Limited” denotes free-text rationales without schema-constrained, source-span-linked components. Check marks indicate evaluated task coverage rather than identical internal representations. All LLM-based baselines used Qwen2.5-7B-Instruct (45) unless otherwise stated.

LLM-Direct, LLM-CoT, and the generation stages of the RAG and KG-LLM methods used sampled decoding with do_sample = true and temperature 0.2. Each method was evaluated in five independent runs using seeds 13, 21, 42, 87, and 100. Within each run, LLM-Self-Consistency generated five samples at temperature 0.7 and applied majority aggregation. RAG methods retrieved eight evidence segments from a retrieval index fixed before test evaluation.

For trainable methods, the five seeds controlled parameter initialization, data order, and other stochastic training operations. For prompting methods, the seeds controlled stochastic decoding. Rule-KG Scoring applied the same deterministic scoring rules in every run, but its input graph was produced by the corresponding seed-specific information-extraction run. Its reported variation therefore reflects upstream extraction and graph construction rather than randomness in the rule scorer. Results with a genuine run-dependent source are reported as mean ± SD over the five predefined runs. A method is reported as a single value only when its complete pipeline is seed-invariant and uses fixed retrieval, fixed rules, and greedy decoding with do_sample = false; in that case, temperature is not applicable. No zero-shot LLM method evaluated with temperature 0.2 is described as deterministic.

Five-category Macro-F1 at the eligible clause–review-dimension level was the primary metric. High-priority F1 treated labels 3–4 as positive. Document-level MAE, Spearman's ρ, and quadratic weighted kappa were calculated after dimension-specific maximum-rule aggregation and served only as complementary metrics. The DHP-FedReg-HHS test split contained 1,853, 1,680, and 1,593 eligible clause–review-dimension instances for executability, behavioral accessibility, and service-support coverage, respectively. The corresponding document-level sample sizes are reported in Supplementary Table A2 and Table 2.

Extraction was evaluated using entity F1 and strict relation F1. Graph consistency was defined as the proportion of predicted relations satisfying the fixed type–relation–type schema.

Explanation quality was evaluated at the same clause–review-dimension level. Benchmark-derived reference components *G*_*i*_were generated from the finalized annotations and predefined structural-gap rules rather than from independent expert judgments of explanation quality. Let *P*_*i*_ denote the predicted components, $P_{i}^{obs}\subseteq P_{i}$ the predicted components requiring evidence support, $C_{i}\subseteq P_{i}^{obs}$ the evidence-linked components, and *M*_*i*_ ⊆ *C*_*i*_ the components whose linked evidence matched the finalized annotations. The instance-level metrics were


$$\begin{array}{l} \;ES_{i}=\frac{\left|C_{i}\right|}{\left|P_{i}^{obs}\right|},\;EC_{i}=\frac{\left|M_{i}\right|}{\left|C_{i}\right|},\;RCP_{i}=\frac{\left|P_{i}\cap G_{i}\right|}{\left|P_{i}\right|},\\ RCC_{i}=\frac{\left|P_{i}\cap G_{i}\right|}{\left|G_{i}\right|}. \end{array}$$

These metrics measure evidence support, evidence correctness, reference-component precision, and reference-component completeness, respectively. Scores were macro-averaged over evaluable instances. Component-matching criteria, missing-link treatment, and zero-denominator rules are specified in Supplementary Appendix A.

### 4.3 Policy element extraction results

This experiment separately evaluated the performance of entity extraction, relationship extraction, and relationship-level evidence support. The experimental results on each major dataset are shown in Table 5. Entity F1 is computed at the span level. Relation F1 requires the correct head entity, tail entity, relation type, and direction. Strict relation F1 further requires that the supporting evidence span entail the complete relation claim. Evidence correctness and hallucination rate are therefore reported at the relation level, not at the entity level. Entity-only baselines such as BERT-CRF do not output relations, so relation-level metrics are marked as not applicable.

**Table 5:** Policy element extraction performance across datasets, mean ± SD over five seeds.

Method	FedReg entity/Rel. F1	MultiEURLEX entity/Rel. F1	BillSum entity/Rel. F1	Strict Rel. F1	Evidence correctness	Hallucination ↓
BERT-CRF	0.781 ± 0.011/—	0.752 ± 0.014/—	0.733 ± 0.016/—	—	—	—
SpERT	0.803 ± 0.010/0.662 ± 0.014	0.776 ± 0.013/0.631 ± 0.017	0.759 ± 0.015/0.612 ± 0.019	0.541 ± 0.018	0.721 ± 0.017	0.074 ± 0.008
CasRel	0.795 ± 0.012/0.681 ± 0.016	0.768 ± 0.015/0.647 ± 0.018	0.751 ± 0.017/0.628 ± 0.020	0.556 ± 0.019	0.708 ± 0.019	0.079 ± 0.009
UIE	0.818 ± 0.009/0.704 ± 0.013	0.792 ± 0.012/0.672 ± 0.016	0.774 ± 0.014/0.651 ± 0.018	0.583 ± 0.017	0.746 ± 0.016	0.066 ± 0.007
Free-form LLM extraction	0.714 ± 0.020/0.563 ± 0.026	0.687 ± 0.024/0.531 ± 0.029	0.671 ± 0.026/0.508 ± 0.031	0.431 ± 0.028	0.651 ± 0.025	0.150 ± 0.018
LLM + schema	0.781 ± 0.016/0.642 ± 0.022	0.752 ± 0.018/0.613 ± 0.025	0.741 ± 0.020/0.596 ± 0.027	0.512 ± 0.024	0.707 ± 0.021	0.108 ± 0.014
LLM + schema + relation template	0.803 ± 0.014/0.701 ± 0.019	0.779 ± 0.016/0.666 ± 0.021	0.768 ± 0.018/0.648 ± 0.024	0.574 ± 0.021	0.740 ± 0.019	0.084 ± 0.012
LLM + evidence span	0.817 ± 0.013/0.718 ± 0.018	0.793 ± 0.015/0.681 ± 0.020	0.784 ± 0.017/0.669 ± 0.022	0.603 ± 0.020	0.807 ± 0.017	0.062 ± 0.010
PRP-KG extraction	0.846 ± 0.010/0.759 ± 0.015	0.821 ± 0.013/0.724 ± 0.018	0.808 ± 0.015/0.703 ± 0.020	0.642 ± 0.018	0.851 ± 0.015	0.043 ± 0.008

PRP-KG improves relation F1, strict relation F1, and relation-level evidence correctness compared with free-form LLM extraction, schema-only prompting, and standard information extraction baselines. The improvement mainly comes from relation templates, relation-level evidence checking, and graph-based feedback with revalidation.

Table 5 reports extraction results. PRP-KG achieves higher relation F1, strict relation F1, and evidence correctness through relation templates, relation-level evidence checking, and graph feedback.

**Figure 6:**
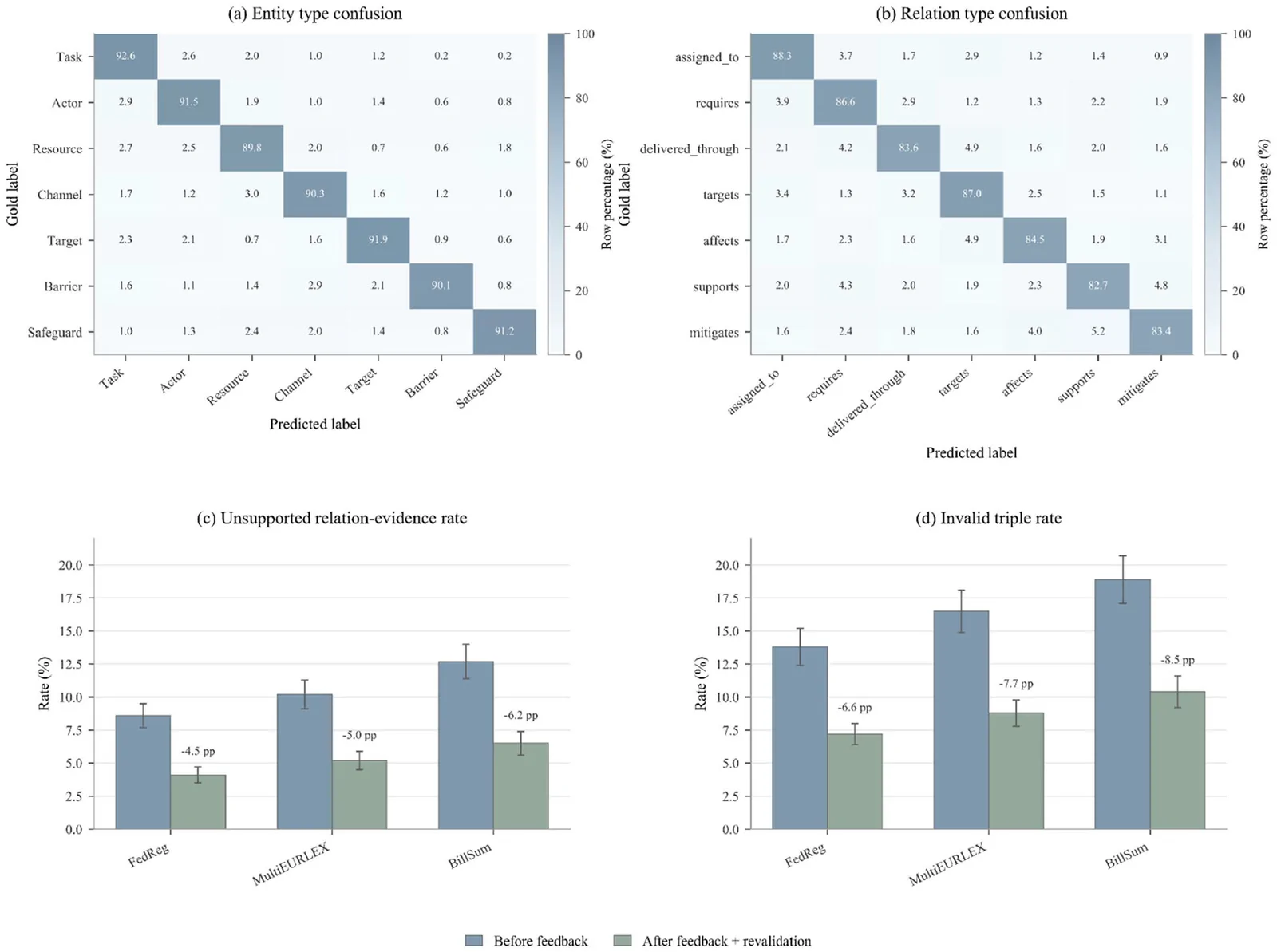
Extraction error distribution before and after graph consistency feedback. The figure contains four panels: **(a)** entity type confusion, **(b)** relation type confusion, **(c)** unsupported relation rate before and after feedback, and **(d)** invalid triple rate before and after feedback.

Figure 6 shows entity and relation confusion, unsupported relation rates, and invalid triple rates before and after feedback. Remaining errors primarily involve cross-clause safeguards or target groups.

### 4.4 Policy knowledge graph construction results

This experiment evaluates policy knowledge graph construction following relation-level extraction and evidence validation. Table 6 reports graph-level rather than extraction-level metrics. Triple validity assesses whether the predicted typed triples satisfy the evidence and schema requirements. Graph consistency measures compliance with predefined structural constraints. Reference-path correctness measures agreement between the model-derived execution paths and the evidence-linked reference paths. These metrics assess consistency with the constructed benchmark and should not be interpreted as direct evidence of real-world policy implementation.

**Table 6:** Effects of graph validation on extraction performance and edge validity.

Method	Triple validity	Edge-validity ratio	Path coverage	Reference-path correctness	Missing actor ↓	Missing resource ↓	Missing channel ↓
Rule-KG	0.720 ± 0.018	0.684 ± 0.021	0.480 ± 0.026	0.512 ± 0.024	0.242 ± 0.018	0.362 ± 0.022	0.320 ± 0.021
LLM-KG	0.784 ± 0.016	0.743 ± 0.020	0.555 ± 0.025	0.584 ± 0.023	0.192 ± 0.016	0.307 ± 0.020	0.275 ± 0.019
GraphRAG-KG	0.818 ± 0.014	0.764 ± 0.019	0.583 ± 0.024	0.617 ± 0.022	0.177 ± 0.015	0.283 ± 0.019	0.254 ± 0.018
Flat KG	0.801 ± 0.016	0.751 ± 0.019	0.571 ± 0.025	0.602 ± 0.023	0.184 ± 0.016	0.294 ± 0.020	0.267 ± 0.019
Two-layer KG	0.829 ± 0.013	0.792 ± 0.017	0.611 ± 0.022	0.648 ± 0.020	0.161 ± 0.014	0.259 ± 0.018	0.238 ± 0.017
PRP-KG	0.846 ± 0.012	0.846 ± 0.015	0.681 ± 0.020	0.704 ± 0.019	0.143 ± 0.013	0.240 ± 0.017	0.222 ± 0.016

Table 6 compares Rule-KG, LLM-KG, GraphRAG-KG, Flat KG, Two-layer KG, and PRP-KG. Flat KG and PRP-KG use the same extracted candidate nodes and typed relations, with Flat KG omitting explicit layer labels. Two-layer KG retains the same entities but merges the support and target-population layers. These structure-matched variants isolate the effect of graph organization while preserving the extracted candidate information. PRP-KG achieved the highest graph consistency and reference-path correctness, whereas GraphRAG-KG achieved the highest triple validity.

**Figure 7:**
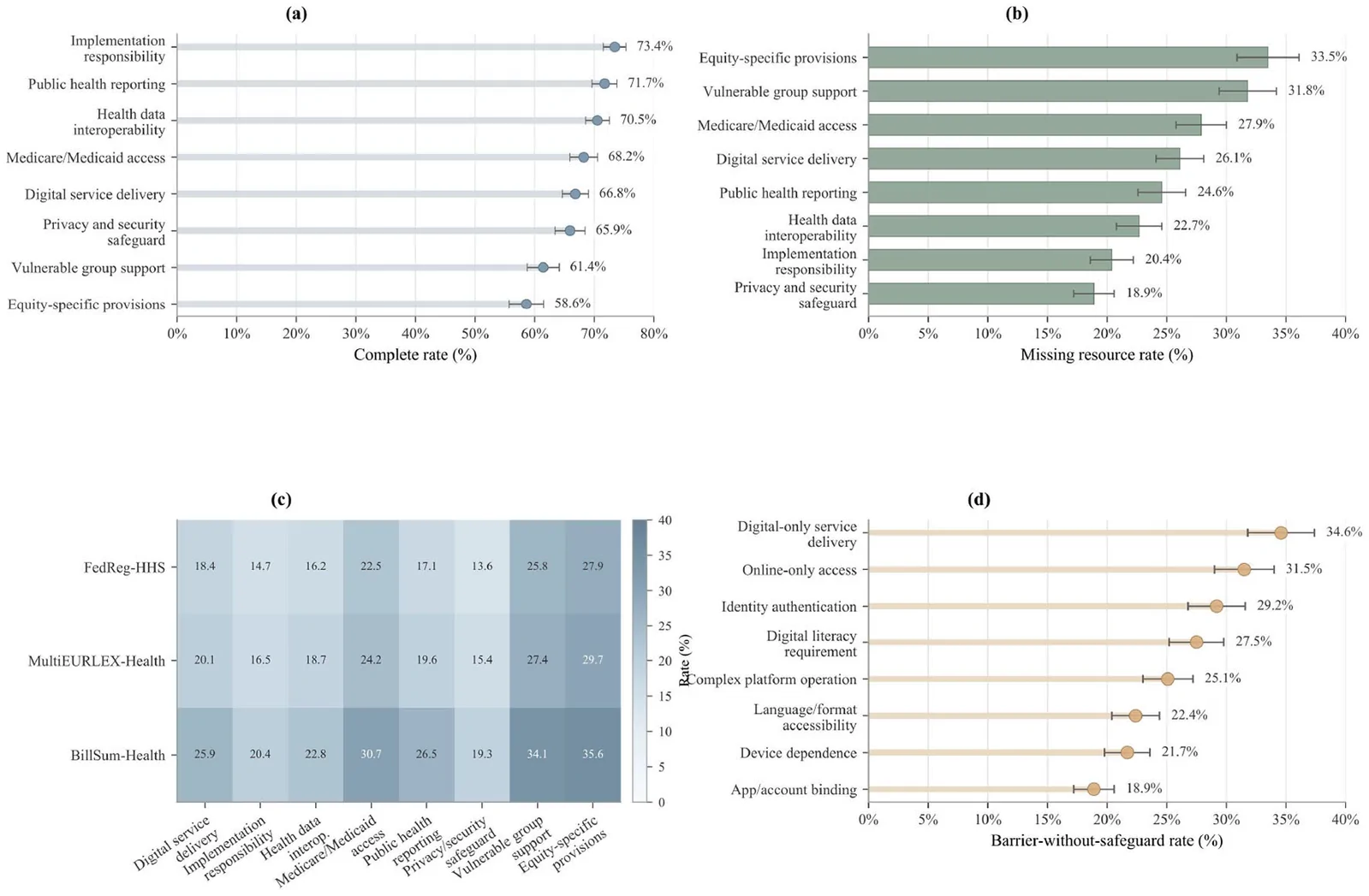
Descriptive distribution of text-implied structural gaps in the policy knowledge graph. This figure illustrates the distribution of obstacles such as incomplete execution paths, lack of resources, absence of service channels, and the absence of safeguard measures in the policy knowledge graph. **(a)** Execution-path completeness. **(b)** Missing-resource rate. **(c)** Missing-service-channel rate. **(d)** Barrier-without-safeguard rate.

Figure 7 presents descriptive distributions of text-implied structural gaps across policy categories and corpora. Among the categories shown, equity-specific provisions and vulnerable-group support had the highest rates of incomplete-path and missing-resource gaps. Missing-service-channel rates were consistently higher in DHP-BillSum-Health than in the other two corpora. Barrier-without-safeguard patterns were most frequent for digital-only service delivery, online-only access, and identity-authentication conditions. These distributions summarize evidence-linked textual patterns and do not establish actual implementation barriers or cross-jurisdictional prevalence.

### 4.5 In-domain and cross-dataset review-signal evaluation

All trainable models were trained and selected using only the DHP-FedReg-HHS training and validation splits. The selected configurations, hyperparameters, and dimension-specific ordinal cutpoints were then applied unchanged to DHP-MultiEURLEX-Health and DHP-BillSum-Health. Table 7 reports corpus-specific MAE and Macro-F1; the remaining metrics are reported only for the DHP-FedReg-HHS test split.

**Table 7:** In-domain and corpus-specific cross-dataset review-signal results, mean ± SD over five predefined runs.

Method	FedReg MAE ↓/Macro-F1 ↑	MultiEURLEX MAE ↓/Macro-F1 ↑	BillSum MAE ↓/Macro-F1 ↑	FedReg mean dimension-specific ρ ↑	FedReg QWK ↑	FedReg high-priority F1 ↑
TF-IDF + LinearSVC	0.892 ± 0.026/0.462 ± 0.019	0.931 ± 0.030/0.441 ± 0.021	0.958 ± 0.033/0.428 ± 0.023	0.402 ± 0.025	0.386 ± 0.026	0.421 ± 0.024
BERT	0.730 ± 0.023/0.574 ± 0.018	0.768 ± 0.026/0.551 ± 0.020	0.791 ± 0.028/0.539 ± 0.021	0.541 ± 0.021	0.512 ± 0.023	0.526 ± 0.022
LEGAL-BERT	0.693 ± 0.021/0.603 ± 0.017	0.724 ± 0.024/0.581 ± 0.019	0.752 ± 0.026/0.562 ± 0.020	0.579 ± 0.020	0.551 ± 0.021	0.553 ± 0.020
Longformer	0.666 ± 0.020/0.628 ± 0.016	0.693 ± 0.023/0.609 ± 0.018	0.689 ± 0.025/0.598 ± 0.020	0.607 ± 0.019	0.574 ± 0.020	0.578 ± 0.019
LLM-direct	0.733 ± 0.029/0.581 ± 0.026	0.771 ± 0.033/0.552 ± 0.028	0.802 ± 0.036/0.531 ± 0.030	0.532 ± 0.030	0.497 ± 0.032	0.518 ± 0.031
LLM-CoT	0.676 ± 0.026/0.619 ± 0.023	0.712 ± 0.030/0.591 ± 0.026	0.736 ± 0.032/0.568 ± 0.027	0.589 ± 0.027	0.558 ± 0.028	0.562 ± 0.028
LLM-self-consistency	0.656 ± 0.023/0.637 ± 0.021	0.684 ± 0.027/0.604 ± 0.024	0.711 ± 0.029/0.586 ± 0.025	0.616 ± 0.024	0.581 ± 0.025	0.584 ± 0.025
BM25-RAG	0.634 ± 0.022/0.651 ± 0.020	0.661 ± 0.025/0.619 ± 0.023	0.689 ± 0.027/0.594 ± 0.024	0.634 ± 0.023	0.602 ± 0.024	0.604 ± 0.024
Dense-RAG	0.613 ± 0.021/0.669 ± 0.019	0.647 ± 0.024/0.636 ± 0.022	0.666 ± 0.026/0.612 ± 0.023	0.651 ± 0.022	0.624 ± 0.023	0.617 ± 0.023
Text-RAG + evidence	0.598 ± 0.020/0.676 ± 0.018	0.631 ± 0.023/0.645 ± 0.021	0.652 ± 0.025/0.621 ± 0.022	0.661 ± 0.021	0.635 ± 0.022	0.626 ± 0.022
Rule-KG scoring	0.621 ± 0.023/0.641 ± 0.021	0.674 ± 0.026/0.602 ± 0.024	0.703 ± 0.029/0.581 ± 0.025	0.632 ± 0.024	0.598 ± 0.025	0.592 ± 0.025
R-GCN	0.574 ± 0.020/0.682 ± 0.018	0.618 ± 0.023/0.654 ± 0.021	0.643 ± 0.025/0.628 ± 0.022	0.672 ± 0.021	0.642 ± 0.022	0.634 ± 0.022
HGT	0.559 ± 0.019/0.696 ± 0.017	0.604 ± 0.022/0.666 ± 0.020	0.628 ± 0.024/0.641 ± 0.021	0.680 ± 0.020	0.657 ± 0.021	0.645 ± 0.021
GraphRAG	0.585 ± 0.022/0.684 ± 0.020	0.616 ± 0.025/0.657 ± 0.022	0.635 ± 0.027/0.635 ± 0.023	0.673 ± 0.023	0.646 ± 0.024	0.636 ± 0.024
LightPROF	0.552 ± 0.020/0.702 ± 0.018	0.589 ± 0.023/0.673 ± 0.020	0.609 ± 0.025/0.648 ± 0.021	0.693 ± 0.020	0.663 ± 0.021	0.643 ± 0.022
EPERM	0.545 ± 0.019/0.708 ± 0.017	0.581 ± 0.022/0.678 ± 0.020	0.603 ± 0.024/0.652 ± 0.021	0.700 ± 0.019	0.671 ± 0.020	0.651 ± 0.021
AtomR	0.542 ± 0.019/0.711 ± 0.017	0.580 ± 0.022/0.681 ± 0.019	0.599 ± 0.024/0.655 ± 0.020	0.702 ± 0.019	0.676 ± 0.020	0.657 ± 0.020
PRP-KG	0.438 ± 0.017/0.736 ± 0.016	0.510 ± 0.021/0.701 ± 0.018	0.540 ± 0.023/0.681 ± 0.020	0.734 ± 0.018	0.694 ± 0.019	0.669 ± 0.019

Macro-F1 was calculated over eligible clause-review-dimension instances. MAE, QWK, and Spearman's ρwere calculated after dimension-specific maximum-rule document aggregation. Corpus-level MAE and FedReg mean dimension-specific ρ are unweighted macro-averages across the three review dimensions. Corpus results were calculated separately and were not pooled. Labels 3–4 were treated as positive for high-priority F1. All settings and cutpoints were fixed using DHP-FedReg-HHS and applied unchanged to the other corpora.

PRP-KG achieved the lowest MAE and highest Macro-F1 on all three corpora. On DHP-FedReg-HHS, it obtained a dimension-macro document-level MAE of 0.438 and a clause-level Macro-F1 of 0.736, compared with 0.542 and 0.711 for AtomR. Its mean dimension-specific Spearman correlation was 0.734, vs. 0.702 for AtomR. PRP-KG also achieved a QWK of 0.694 and a high-priority review-signal F1 of 0.669.

Without corpus-specific retraining, tuning, or cutpoint recalibration, PRP-KG achieved Macro-F1 scores of 0.701 on DHP-MultiEURLEX-Health and 0.681 on DHP-BillSum-Health, decreases of 3.5 and 5.5 percentage points from its in-domain result. These results provide cross-dataset evidence under the shared annotation framework but do not establish generalization across jurisdictions, languages, policy domains, or healthcare systems.

Table 8 reports dimension-specific document-level results for DHP-FedReg-HHS. PRP-KG achieved the lowest MAE in all three dimensions and the highest correlation for behavioral accessibility and service-support coverage. AtomR obtained a slightly higher executability correlation than PRP-KG (0.735 vs. 0.731), showing that PRP-KG did not lead on every dimension-specific ranking metric.

**Table 8:** Dimension-specific document-level results on DHP-FedReg-HHS, mean ± SD over five seeds.

Method	Executability MAE ↓	Executability ρ ↑	Behavioral-accessibility MAE ↓	Behavioral-accessibility ρ ↑	Service-support coverage MAE ↓	Service-support coverage ρ ↑
Longformer	0.604 ± 0.020	0.631 ± 0.021	0.681 ± 0.023	0.602 ± 0.024	0.713 ± 0.025	0.587 ± 0.026
Dense-RAG	0.548 ± 0.019	0.681 ± 0.020	0.623 ± 0.022	0.647 ± 0.023	0.667 ± 0.024	0.625 ± 0.025
HGT	0.501 ± 0.018	0.714 ± 0.019	0.574 ± 0.021	0.673 ± 0.022	0.602 ± 0.023	0.652 ± 0.024
GraphRAG	0.521 ± 0.020	0.702 ± 0.022	0.594 ± 0.023	0.668 ± 0.023	0.641 ± 0.025	0.648 ± 0.025
LightPROF	0.486 ± 0.018	0.726 ± 0.019	0.558 ± 0.021	0.689 ± 0.021	0.612 ± 0.023	0.663 ± 0.024
EPERM	0.497 ± 0.019	0.719 ± 0.020	0.545 ± 0.020	0.701 ± 0.021	0.594 ± 0.022	0.681 ± 0.023
AtomR	0.472 ± 0.018	**0.735** **±** **0.018**	0.552 ± 0.020	0.697 ± 0.021	0.603 ± 0.022	0.674 ± 0.023
**PRP-KG**	**0.421** **±** **0.017**	0.731 ± 0.018	**0.437** **±** **0.018**	**0.736** **±** **0.019**	**0.456** **±** **0.020**	**0.735** **±** **0.020**

Documents without an eligible clause for a dimension were excluded. The resulting sample sizes were *N*_*exe*_= 1,124, *N*_*acc*_= 1,037, and *N*_sup_ = 982. Spearman's ρ and MAE were calculated using the same dimension-specific document sets. Bold values indicate the best result in each column.

**Figure 8:**
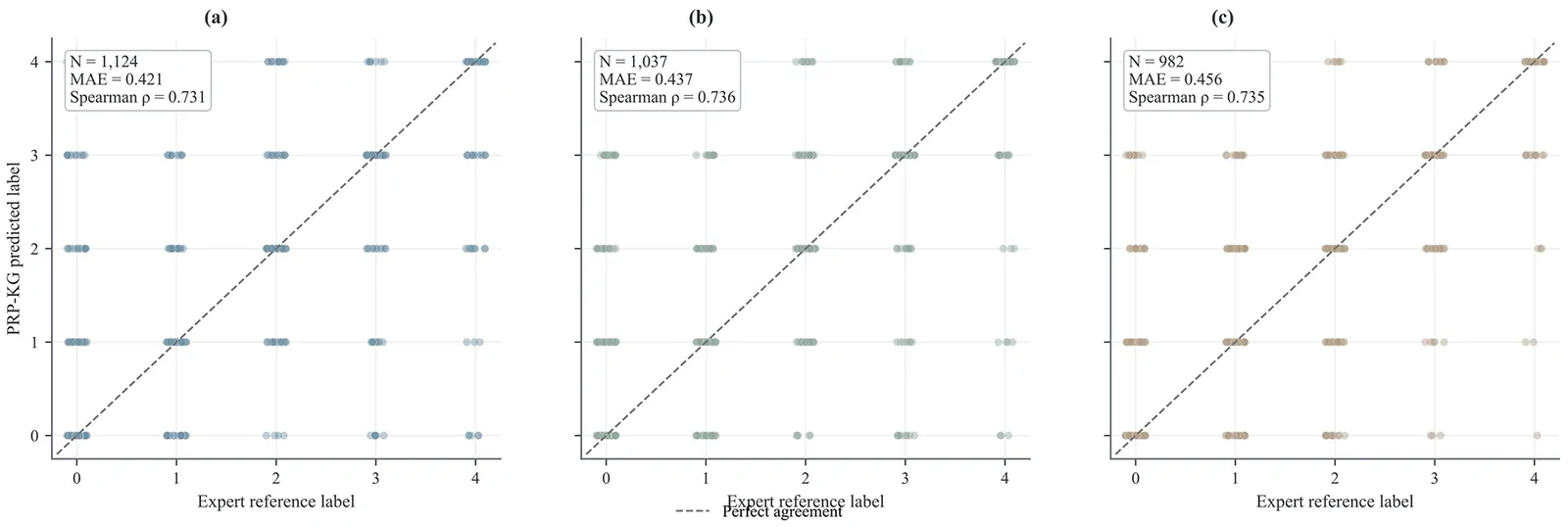
PRP-KG predictions vs. document-level reference labels for the three review dimensions. Points represent prediction–reference pairs after dimension-specific maximum-rule aggregation; the diagonal denotes perfect agreement. **(a)** Executability. **(b)** Behavioral accessibility. **(c)** Service-support coverage.

Figure 8 compares PRP-KG predictions with reference labels after dimension-specific maximum-rule aggregation. The service-support-coverage panel shows greater dispersion around the perfect-agreement line than the other two panels. These results measure agreement with text-implied review labels, not observed implementation quality, realized accessibility, or actual service provision.

### 4.6 Ablation study

Table 9 reports component-removal and controlled structural ablations on the DHP-FedReg-HHS test split. The no-layer-label and merged-layer variants use the same extracted candidate nodes and edges as full PRP-KG. They therefore isolate the effects of layer organization on layer-aware validation, structural-gap scoring, and explanation construction rather than on extraction. Relation F1 remains unchanged and is not repeated. Removing target nodes constitutes an information-removal ablation and does not independently establish the superiority of the three-layer architecture.

**Table 9:** Ablation results on the DHP-FedReg-HHS test split.

Variant	Relation F1	Graph consistency	Exec. MAE ↓	Access. MAE ↓	Support-coverage MAE ↓	Macro-F1 ↑	Reference-component precision ↑
Full PRP-KG	0.759 ± 0.015	0.846 ± 0.015	0.421 ± 0.017	0.437 ± 0.018	0.456 ± 0.020	0.736 ± 0.016	0.781 ± 0.018
w/o schema constraint	0.642 ± 0.022	0.731 ± 0.024	0.538 ± 0.024	0.584 ± 0.027	0.596 ± 0.029	0.641 ± 0.023	0.602 ± 0.025
w/o risk-template schema	0.681 ± 0.020	0.772 ± 0.022	0.507 ± 0.023	0.548 ± 0.026	0.569 ± 0.028	0.671 ± 0.022	0.657 ± 0.024
w/o evidence-based validation	0.742 ± 0.017	0.823 ± 0.018	0.451 ± 0.019	0.487 ± 0.022	0.506 ± 0.024	0.704 ± 0.019	0.619 ± 0.027
w/o feedback re-query	0.709 ± 0.019	0.781 ± 0.021	0.486 ± 0.022	0.512 ± 0.024	0.529 ± 0.026	0.686 ± 0.021	0.671 ± 0.023
Filter-only validation	0.724 ± 0.018	0.809 ± 0.020	0.463 ± 0.020	0.494 ± 0.023	0.517 ± 0.025	0.698 ± 0.020	0.694 ± 0.022
Same extracted nodes/edges, no layer labels	—	0.751 ± 0.023	0.479 ± 0.021	0.521 ± 0.025	0.544 ± 0.027	0.682 ± 0.022	0.702 ± 0.022
Same extracted nodes/edges, merged support-target layers	—	0.792 ± 0.020	0.452 ± 0.020	0.489 ± 0.022	0.512 ± 0.024	0.707 ± 0.019	0.731 ± 0.021
w/o target nodes	0.741 ± 0.017	0.812 ± 0.019	0.438 ± 0.018	0.563 ± 0.026	0.607 ± 0.029	0.664 ± 0.023	0.691 ± 0.023
w/o mitigation edge modeling	0.746 ± 0.017	0.818 ± 0.018	0.435 ± 0.018	0.542 ± 0.025	0.529 ± 0.025	0.681 ± 0.022	0.708 ± 0.022
w/o disparity term	0.751 ± 0.016	0.833 ± 0.017	0.429 ± 0.017	0.456 ± 0.020	0.573 ± 0.028	0.699 ± 0.021	0.739 ± 0.020

Relation F1 and graph consistency are relation-level metrics; Macro-F1 is calculated over eligible clause–review-dimension instances; MAE is calculated after maximum-rule document aggregation; and reference-component precision is calculated over explanation-evaluable instances. The no-layer-label and merged-layer variants retain the full model's extracted nodes and relations; their graph-consistency values reflect layer-aware validation rules rather than changes in extraction. Seed-dependent results are reported as mean ± SD over five runs.

Schema removal produced the largest reductions in relation F1 and graph consistency. Removing evidence-based validation most strongly reduced reference-component precision, whereas feedback re-query performed better than filter-only validation. Removing target nodes, mitigation-edge modeling, or the disparity term primarily affected behavioral-accessibility and service-support-coverage results. These findings support the contribution of the tested components within the constructed benchmark, but do not independently validate the rubric or demonstrate real-world policy effects.

For the same-extracted-node/edge variants, Relation F1 is not re-reported because the extraction outputs are fixed. These variants test how layer organization affects graph validation, scoring, and explanation quality. The “w/o target nodes” variant is an information-removal test.

Overall, schema constraints contribute most to relation validity and graph consistency. Evidence validation improves reference-component precision, and feedback re-query outperforms filter-only validation. Target nodes, mitigation edges, and the disparity term are especially important for behavioral accessibility and support-coverage prediction. These results indicate that the combined design approach has certain advantages, but the findings should still be interpreted as verification results for the system's overall performance.

### 4.7 Controlled robustness and cross-dataset evaluation

Table 10 reports controlled perturbation tests on DHP-FedReg-HHS and corpus-specific cross-dataset evaluations conducted without additional tuning or ordinal-cutpoint recalibration. OCR-style noise, meaning-preserving paraphrasing, and clause truncation were treated as controlled stress tests rather than as simulations of naturally occurring policy-document corruption. PRP-KG achieved the highest high-priority review-signal F1 in all reported settings, although performance declined as OCR corruption increased and under clause truncation. The largest cross-dataset decrease occurred on DHP-BillSum-Health, indicating sensitivity to longer legislative documents with dispersed obligations. These results demonstrate relative stability under the specified experimental conditions, not robustness in operational policy environments.

**Table 10:** Controlled robustness and corpus-specific cross-dataset results.

Setting	LLM-CoT high-priority F1	Dense-RAG high-priority F1	GraphRAG high-priority F1	PRP-KG high-priority F1	PRP-KG document-level MAE	PRP-KG evidence correctness
Clean	0.586 ± 0.028	0.636 ± 0.023	0.653 ± 0.024	0.704 ± 0.019	0.438 ± 0.017	0.851 ± 0.015
OCR noise 5%	0.551 ± 0.030	0.608 ± 0.025	0.621 ± 0.026	0.681 ± 0.021	0.486 ± 0.020	0.827 ± 0.017
OCR noise 10%	0.523 ± 0.032	0.584 ± 0.027	0.597 ± 0.028	0.656 ± 0.023	0.521 ± 0.022	0.796 ± 0.020
OCR noise 15%	0.491 ± 0.034	0.552 ± 0.030	0.568 ± 0.031	0.621 ± 0.026	0.568 ± 0.025	0.751 ± 0.024
Paraphrased clauses	0.547 ± 0.031	0.617 ± 0.026	0.628 ± 0.027	0.674 ± 0.022	0.501 ± 0.021	0.813 ± 0.018
Clause truncation 20%	0.519 ± 0.033	0.581 ± 0.028	0.596 ± 0.029	0.649 ± 0.025	0.542 ± 0.024	0.779 ± 0.022
Cross-agency	0.534 ± 0.031	0.594 ± 0.027	0.611 ± 0.028	0.667 ± 0.023	0.513 ± 0.022	0.801 ± 0.019
Cross-policy type	0.503 ± 0.034	0.572 ± 0.029	0.584 ± 0.030	0.632 ± 0.026	0.568 ± 0.026	0.762 ± 0.024
FedReg → MultiEURLEX	0.511 ± 0.033	0.584 ± 0.028	0.602 ± 0.029	0.651 ± 0.025	0.536 ± 0.024	0.784 ± 0.023
FedReg → BillSum	0.492 ± 0.035	0.561 ± 0.030	0.579 ± 0.031	0.623 ± 0.028	0.579 ± 0.027	0.741 ± 0.026

Mean ± SD over five predefined runs.

High-priority F1 was calculated over eligible clause–review-dimension instances, document-level MAE after maximum-rule aggregation, and evidence correctness over explanation-evaluable instances. Models, hyperparameters, and ordinal cutpoints were fixed using DHP-FedReg-HHS. Cross-dataset corpora were evaluated separately without retuning or recalibration. Perturbation results were calculated on the same fixed robustness subset, with Clean denoting its unperturbed version. Seed-dependent values are reported as mean ± SD over five runs.

OCR-style noise was generated through controlled character deletions, insertions, and substitutions at the specified corruption rates. Paraphrases were produced using a rewriting model different from the PRP-KG backbone; 20% were manually checked, and only clauses that preserved the obligation, target group, relation direction, and evidential meaning were retained. Evidence spans were remapped to the rewritten text. For the truncation condition, 20% of clause content was removed according to the fixed procedure in Supplementary Appendix B, while document titles and metadata were retained. Because truncation may remove label-relevant evidence, this condition measures performance under incomplete input rather than meaning-preserving robustness. These tests do not reproduce cross-references, definition dependencies, revisions, attachments, or implementation-manual context encountered in operational policy review.

### 4.8 Parameter sensitivity analysis

Table 11 reports one-factor sensitivity analyses conducted exclusively on the DHP-FedReg-HHS validation split. The relation-consistency threshold, maximum number of feedback re-query rounds, and retrieval depth were varied separately, while all other settings were fixed. Five-category validation Macro-F1 over eligible clause–review-dimension instances was the sole selection criterion. Relation F1, graph consistency, five-category document-label MAE, and runtime are reported only as complementary diagnostic measures.

**Table 11:** One-factor parameter sensitivity on the DHP-FedReg-HHS validation split.

Parameter	Value	Validation Macro-F1 ↑	Relation F1 ↑	Graph consistency ↑	Five-category document-label MAE ↓	Runtime/document ↓
Consistency threshold τ_*c*_	0.60	0.712 ± 0.009	0.736 ± 0.018	0.808 ± 0.020	0.472 ± 0.021	31.7 ± 2.1 s
	0.70	0.749 ± 0.007	0.752 ± 0.016	0.839 ± 0.017	0.444 ± 0.018	34.2 ± 2.4 s
	0.75	0.761 ± 0.006	0.759 ± 0.015	0.846 ± 0.015	0.438 ± 0.017	35.6 ± 2.5 s
	0.80	0.754 ± 0.007	0.754 ± 0.016	0.852 ± 0.014	0.447 ± 0.018	38.8 ± 2.7 s
	0.85	0.742 ± 0.009	0.742 ± 0.018	0.858 ± 0.013	0.466 ± 0.020	41.5 ± 3.0 s
Maximum re-query rounds *R*	0	0.688 ± 0.011	0.713 ± 0.020	0.781 ± 0.022	0.503 ± 0.023	19.1 ± 1.8 s
	1	0.738 ± 0.008	0.741 ± 0.017	0.821 ± 0.019	0.461 ± 0.020	26.3 ± 2.2 s
	2	0.761 ± 0.006	0.759 ± 0.015	0.846 ± 0.015	0.438 ± 0.017	35.6 ± 2.5 s
	3	0.760 ± 0.006	0.763 ± 0.015	0.853 ± 0.014	0.436 ± 0.017	47.9 ± 3.6 s
Retrieved evidence top-*K*	3	0.724 ± 0.009	0.731 ± 0.018	0.823 ± 0.018	0.462 ± 0.020	29.7 ± 2.0 s
	5	0.747 ± 0.007	0.748 ± 0.017	0.837 ± 0.017	0.446 ± 0.018	32.4 ± 2.3 s
	8	0.761 ± 0.006	0.759 ± 0.015	0.846 ± 0.015	0.438 ± 0.017	35.6 ± 2.5 s
	12	0.760 ± 0.006	0.761 ± 0.015	0.849 ± 0.015	0.439 ± 0.018	42.8 ± 3.1 s

Validation Macro-F1 was calculated over eligible clause–review-dimension instances. Relation F1 and graph consistency are relation-level diagnostic metrics. Five-category document-label MAE is the unweighted mean of the three dimension-specific document-level MAEs after maximum-rule aggregation. It is expressed in ordinal-category steps on the 0–4 scale. Runtime was measured under the implementation environment reported in Section 4.2. Values are mean ± SD over five runs.

The selected relation-consistency threshold was τ_*c*_ = 0.75, which achieved the highest validation Macro-F1 of 0.761 ± 0.006 among the evaluated threshold settings. Higher thresholds increased graph consistency but reduced validation Macro-F1 because additional filtering removed potentially valid relations. Two re-query rounds were selected because *R* = 3 produced a slightly lower validation Macro-F1 (0.760 ± 0.006) while increasing runtime from 35.6 ± 2.5 to 47.9 ± 3.6 s per document. Retrieval depth *K* = 8 was selected because increasing the retrieval depth to *K* = 12 likewise reduced validation Macro-F1 slightly and increased computational cost. All settings were fixed before the in-domain test and cross-dataset evaluation.

**Figure 9:**
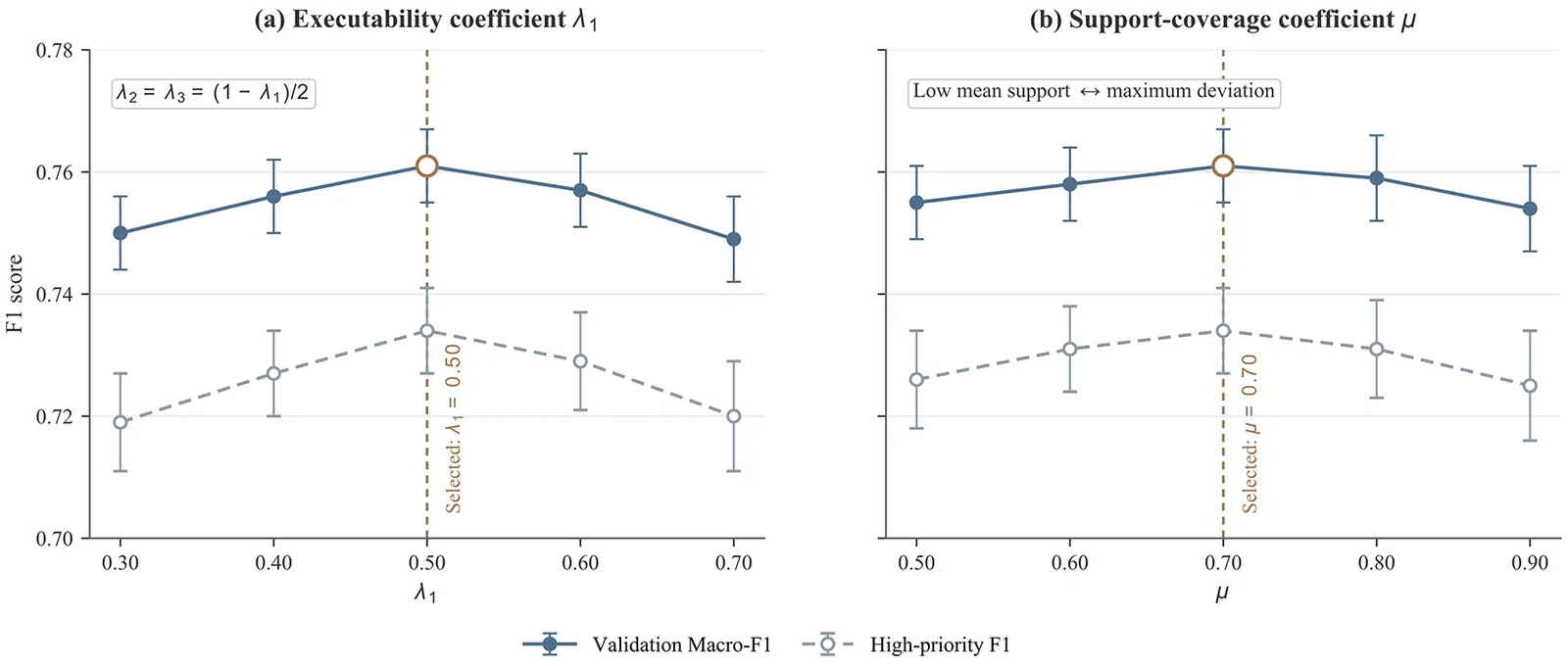
One-factor sensitivity of validation Macro-F1 and high-priority F1 to **(a)** the execution-chain weight λ_1_ and **(b)** the service-support coverage weight μon the DHP-FedReg-HHS validation split. Dashed vertical lines mark the values selected by validation Macro-F1; error bars show SD over five runs.

Figure 9 reports the one-factor sensitivity of five-category validation Macro-F1 and the complementary high-priority F1 to local changes in two representative structural-gap scoring weights. In Figure 9a, the execution-chain weight λ_1_ was varied while maintaining λ_2_ = λ_3_ = (1 − λ_1_)/2. Both metrics reached their highest mean values at the selected setting λ_1_ = 0.50, with gradual changes around this point. In Figure 9b, μ controlled the balance between the mean support deficit and maximum between-group disparity. The selected value μ = 0.70 likewise achieved the highest validation Macro-F1, while nearby settings produced only modest changes. High-priority F1 is shown only as a complementary diagnostic metric and was not used for parameter selection. These results indicate local stability within the DHP-FedReg-HHS validation split and do not establish universally optimal scoring weights.

### 4.9 Operational efficiency

Table 12 reports document-level computational cost under the same hardware environment and fixed document sample. All methods used the same document inputs and batch size, while method-specific retrieval, prompting, and graph-processing components were retained. PRP-KG required more processing stages and more LLM calls than direct prompting because it included evidence retrieval, schema-constrained extraction, graph validation, feedback re-query when triggered, and explanation-path construction. Under the evaluated implementation, its mean runtime was lower than Dense-RAG and GraphRAG but higher than LLM-Direct. These measurements reflect implementation-specific computational cost and do not assess expert-review time, workflow usability, or operational effectiveness.

**Table 12:** Document-level computational cost under the evaluated implementation.

Method	Runtime/document ↓	LLM calls/document ↓	Total LLM tokens/document ↓
LLM-direct	21.4 ± 3.2 s	1.0	6.1k
LLM-CoT	38.7 ± 5.1 s	1.0	8.4k
Dense-RAG	52.3 ± 6.4 s	1.0	10.7k
GraphRAG	74.6 ± 8.9 s	1.0	12.9k
PRP-KG	35.6 ± 2.5 s	2.3	9.8k

Runtime is wall-clock time from document input to final review-signal and explanation output, including method-specific retrieval and graph-processing stages. Measurements used the same hardware, fixed document sample, batch size, model checkpoint, and generation settings where applicable. Runtime is reported as mean ± SD over repeated document-level measurements. LLM calls and total token counts are averages per document; total tokens include both prompt/input and generated/output tokens. The average of 2.3 LLM calls for PRP-KG reflects conditional feedback re-query. Index construction time is excluded. These measurements characterize computational cost only.

Under the default configuration, PRP-KG reached a peak GPU-memory allocation of 19.6 ± 1.4GB during schema-constrained extraction and 8.4 ± 0.7GB during graph scoring. These stage-specific peak values were measured separately and should not be added. The DHP-FedReg-HHS retrieval index required 3.8 GB of storage, excluding the source-document collection and index-construction workspace. Mean end-to-end throughput was 101 ± 8 documents per GPU hour under the same batch and inference configuration. The throughput is consistent with the reported mean runtime of 35.6 s per document. All measurements depend on document length, batching, software implementation, retrieval infrastructure, and hardware, and therefore should not be interpreted as deployment benchmarks.

### 4.10 Explanation quality and case-level analysis

Explanation quality was evaluated against evidence-linked reference components derived from the finalized entity, relation, and evidence-span annotations using the fixed schema and structural-gap rules. Table 13 reports four automatically computed metrics at the clause-level review-flag instance. PRP-KG achieves the highest mean scores across all four metrics, although its reference-component completeness of 0.724 indicates that some required components remain unrecovered.

**Table 13:** Automated explanation-quality evaluation, mean ± SD over five predefined runs.

Method	Evidence support ↑	Evidence correctness ↑	Reference-component precision ↑	Reference-component completeness ↑
LLM-CoT	0.583 ± 0.031	0.521 ± 0.034	0.512 ± 0.035	0.471 ± 0.038
Text-RAG + evidence	0.701 ± 0.025	0.642 ± 0.028	0.624 ± 0.030	0.553 ± 0.033
GraphRAG	0.742 ± 0.023	0.681 ± 0.026	0.671 ± 0.027	0.608 ± 0.031
EPERM	0.786 ± 0.021	0.719 ± 0.024	0.718 ± 0.025	0.641 ± 0.029
AtomR	0.764 ± 0.022	0.704 ± 0.025	0.701 ± 0.026	0.633 ± 0.030
PRP-KG	0.872 ± 0.015	0.851 ± 0.015	0.781 ± 0.018	0.724 ± 0.022

Metrics are macro-averaged over clause-level review-flag instances. Reference components were deterministically derived from the finalized entity, typed-relation, and evidence-span annotations using the predefined schema and structural-gap rules. No independently annotated explanation paths or subjective human ratings were used.

**Figure 10:**
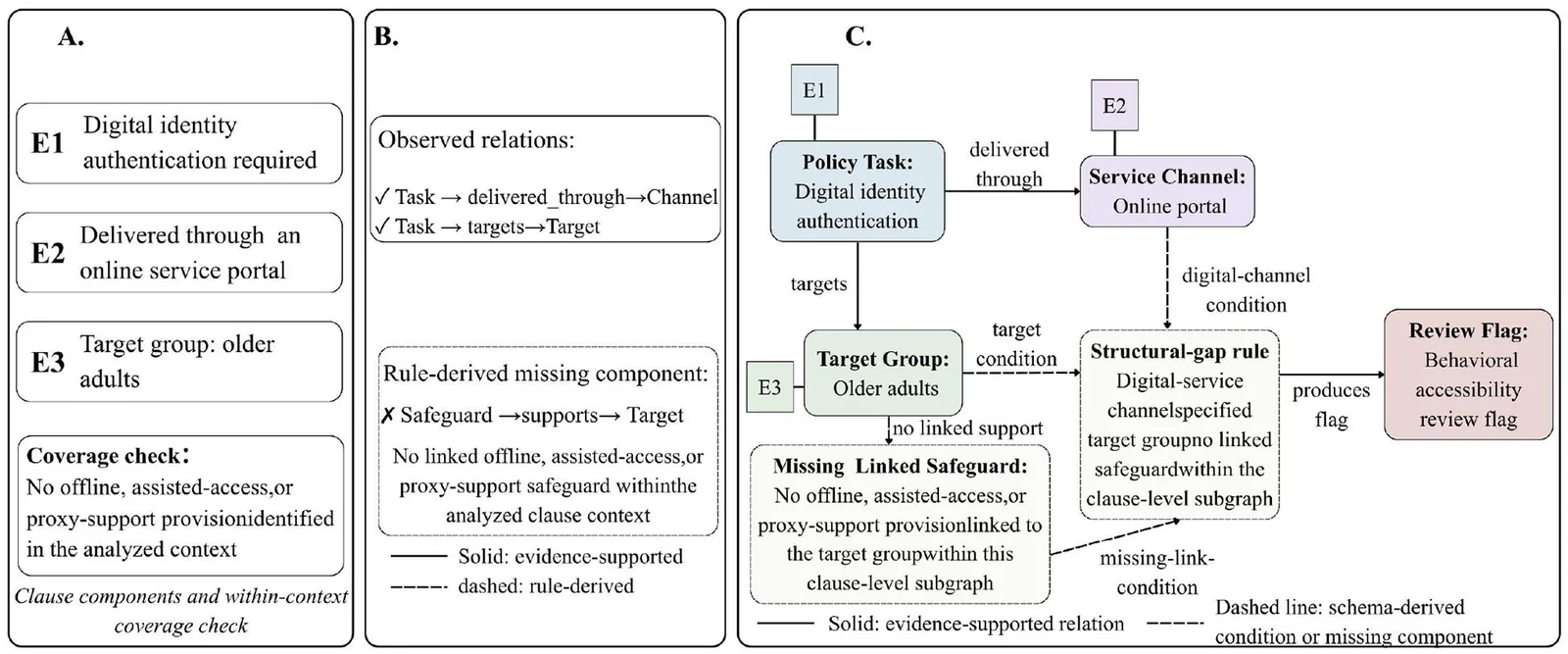
Evidence-linked clause-level explanation subgraph for a behavioral-accessibility review flag. Solid links represent source-supported relations, whereas dashed links represent rule-derived conditions or missing safeguard links. **(A)** Evidence-linked clause components. **(B)** Schema-constrained path. **(C)** Case-level explanation subgraph + review flag.

Figure 10 presents a descriptive clause-level case from a publicly accessible policy document. The analyzed provision links a digital identity-authentication task to an online service portal and identifies older adults as the target group. Within the extracted clause-level subgraph, PRP-KG produces a behavioral-accessibility review flag because no offline alternative, assisted-access mechanism, or proxy-support safeguard is linked to that target group.

The figure distinguishes source-supported entities and relations from rule-derived missing-link conditions. It illustrates how observed evidence components and a predefined structural-gap rule jointly produce an evidence-linked explanation. The case does not establish that relevant safeguards are absent elsewhere in the document or broader policy environment, and it does not evaluate real-world implementation, accessibility, or equity outcomes.

### 4.11 Statistical significance

Statistical comparisons were conducted on the DHP-FedReg-HHS test split. For each seed, clause-level reference labels and predictions were aggregated separately by document and review dimension using the prespecified maximum rule. Documents without an eligible clause for a given dimension were excluded from the corresponding analysis. After document-level aggregation, the sample sizes were *N*_exe_= 1,124, *N*_acc_= 1,037, and *N*_sup_= 982, consistent with Supplementary Table A2.

For baseline *b* and review dimension *d*, the paired effect was defined as


$$\begin{array}{c} \Delta {\text{MAE}}_{b,d}={\text{MAE}}_{b,d}-{\text{MAE}}_{\text{PRP}-\text{KG},d}, \end{array}$$

where positive values favor PRP-KG. Uncertainty was estimated using *B* = 10, 000 paired document-family cluster-bootstrap replicates. Within each replicate, the same sampled document families were applied to both methods. Dimension-specific paired MAE differences were calculated separately for the five seeds and then averaged across seeds to obtain the replicate-level effect.

Percentile 95% confidence intervals (CIs) were derived from the bootstrap distributions. For replicate effects Δ_1_, …, Δ_*B*_, two-sided bootstrap *p*-values were calculated as


$$\begin{array}{l} p=\min\{1,\;2\;\min[\frac{1+\sum_{r=1}^{B}𝕀\left(\Delta_{r}\leq 0\right)}{B+1},\\ \;\frac{1+\sum_{r=1}^{B}𝕀\left(\Delta_{r}\geq 0\right)}{B+1}]\}, \end{array}$$

where the +1 correction prevents zero *p*-values. The resulting *p*-values were adjusted jointly across the 21 prespecified dimension-specific comparisons using the Holm procedure. Overall effects were calculated by equally averaging the three dimension-specific effects within each replicate. Their CIs were descriptive and were not included in the multiplicity adjustment.

As shown in Table 2, PRP-KG had significantly lower document-level MAE than Longformer, Dense-RAG, HGT, and GraphRAG in all three dimensions after Holm adjustment. Compared with LightPROF, EPERM, and AtomR, the differences were significant for behavioral accessibility and service-support coverage but not for executability.

Evidence of lower MAE was more consistent across the strongest baselines for behavioral accessibility and service-support coverage than for executability. These comparisons assess agreement with maximum-rule-aggregated, text-implied review labels on the constructed benchmark; they do not establish improvements in reviewer performance, realized policy implementation, behavioral accessibility, or service provision.

## 5 Discussion

This study presents PRP-KG, a large language model-enhanced knowledge graph framework for identifying text-implied structural gaps in digital health policy documents. PRP-KG extracts policy tasks, actors, resources, service channels, target groups, barriers, and safeguards; organizes them using a shared Policy–Implementation–Population schema; and derives review signals from missing or incomplete graph relations. By binding extracted components and resulting subgraphs to identifiable evidence spans, the framework supports structured and inspectable document screening.

The results suggest that combining schema-constrained extraction, graph validation, and structural-gap scoring improves agreement with the constructed benchmark. Schema constraints reduce structurally invalid outputs, while graph validation checks extracted entities and relations against predefined type, relation, and execution rules. Feedback-based re-extraction recovered some missing or mismatched components but cannot eliminate hallucinated entities, unsupported relations, retrieval omissions, or error propagation. When the source text is ambiguous or incomplete, repeated extraction may preserve or amplify an initial error (46–48).

The graph representation is particularly useful for identifying omissions expressed through incomplete policy-execution relations. For example, a clause may require digital identity authentication through an online channel and identify older adults as a target group without linking that group to an offline alternative, assisted-access mechanism, or proxy-support safeguard. PRP-KG represents this condition as a missing link and directs reviewer attention to the corresponding evidence. However, the identified safeguard may be specified elsewhere in the document, in supporting regulations, or in implementation materials beyond the analyzed context (49).

PRP-KG achieved stronger average benchmark performance than most evaluated baselines, although the magnitude and statistical consistency of the differences varied by review dimension. Paired document-family cluster-bootstrap comparisons showed more consistent reductions in document-level MAE for behavioral accessibility and service-support coverage than for executability. Relative to LightPROF, EPERM, and AtomR, the executability differences did not reach the prespecified significance threshold after Holm adjustment. This may reflect the dependence of executability on dispersed provisions, definitions, cross-references, administrative capacity, and other conditions that are not fully captured by clause-level relations. PRP-KG should therefore be interpreted as a task-specific screening framework rather than a general replacement for policy-reasoning methods.

Performance differences may partly reflect alignment among the extraction schema, structural-gap rules, and annotation rubric. Because these components share the same policy-execution concepts, PRP-KG may detect schema-aligned gaps more readily than concerns expressed indirectly or outside the predefined categories. The benchmark consequently measures agreement with a partly schema-aligned annotation framework rather than conformity with an absolute policy standard. This potential schema–rubric circularity should be considered when comparing PRP-KG with less task-specific baselines.

The controlled robustness and cross-dataset results provide bounded evidence. PRP-KG showed relatively smaller degradation than several baselines under the specified OCR-style corruption, meaning-preserving paraphrasing, and incomplete-input truncation conditions. These perturbations do not reproduce the full complexity of policy documents containing dispersed definitions, cross-references, amendments, attachments, or implementation manuals. Moreover, DHP-MultiEURLEX-Health and DHP-BillSum-Health shared the same task definition and annotation framework, although no retuning or ordinal-cutpoint recalibration was performed. The results therefore demonstrate transfer within a shared evaluation setting rather than independent external validation across jurisdictions, languages, legal systems, or health-service contexts.

The explanation evaluation was also tied to the benchmark design. Reference components were deterministically derived from the finalized entity, typed-relation, and evidence-span annotations using the predefined schema and structural-gap rules. The reported metrics therefore assess evidence support, evidence correctness, and recovery of benchmark-derived components rather than agreement with independently annotated explanation paths or subjective expert ratings. Figure 10 illustrates an inspectable, source-linked rationale, but such a rationale does not independently verify policy meaning or demonstrate improved expert judgment.

Several limitations define the scope of the findings. PRP-KG identifies text-implied structural gaps rather than actual implementation, service use, accessibility, health outcomes, or equity. Its extraction, retrieval, and feedback stages remain vulnerable to hallucination, omission, and error propagation. The current schema does not cover fiscal sustainability, privacy, cybersecurity, interoperability, political feasibility, or interagency coordination. Uneven representation of jurisdictions and population groups may also lead to variable model behavior (33). Accordingly, every flagged condition must remain open to inspection, correction, or rejection by qualified professionals. Prospective deployment would also require documented data and model versions, retained evidence trails, uncertainty and abstention mechanisms, access controls, bias audits, applicable privacy and regulatory review, and procedures for contesting or correcting outputs.

Future research should evaluate PRP-KG using independently constructed and locally annotated corpora and, in prospective studies, determine whether its review signals improve reviewer consistency, efficiency, or error detection. Combining policy text with independently collected implementation evidence—such as service-use data, complaint records, digital-access indicators, regional resource distributions, and population-level outcomes—could support validation beyond text-implied gaps. Further study should address cross-document retrieval, uncertainty-aware extraction, explicit abstention, broader policy schemas, bias auditing, and reviewer interfaces supporting correction, rejection, escalation, and audit-log retention.

## 6 Conclusion

This study presented PRP-KG, a large language model-enhanced knowledge graph framework for screening for text-implied gaps in execution-specification, accessibility-safeguard, and service-support-coverage in digital health policy documents. The framework replaces direct document-level judgments with structured analysis of policy-execution units through schema-constrained extraction, evidence-span binding, Policy–Implementation–Population graph construction, graph-consistency feedback, and structural-gap review-signal scoring.

On the constructed benchmark, PRP-KG achieved better average performance than most evaluated baselines, particularly for behavioral-accessibility and service-support-coverage signals. Improvements in executability screening were smaller, and comparisons with LightPROF, EPERM, and AtomR did not reach the prespecified significance threshold after Holm adjustment. Explanation evaluation also showed stronger evidence linkage, although component recovery remained incomplete.

Overall, PRP-KG offers an evidence-based approach to prioritizing passages that may contain incomplete execution specifications, unmitigated digital-access barriers, or unlinked service-support provisions. Its outputs require professional contextual verification and should not be interpreted as measurements of realized policy implementation, accessibility, service provision, or equity outcomes.

## Data Availability

The original contributions presented in the study are included in the article/Supplementary material, further inquiries can be directed to the corresponding author.
