# Supplementary material for: A large language model-enhanced knowledge graph framework for text-implied public health policy gap screening: digital health executability, behavioral accessibility, and service-support coverage
Source: Front Public Health. 2026 Sep 7;14:1937335. doi: 10.3389/fpubh.2026.1937335 (PMC13590772; doi:10.3389/fpubh.2026.1937335)
Supplement: Supplementary file 1 [file Supplementary_file_1.docx]

**Supplementary Appendix A**

**Data Provenance, Benchmark Construction, and Detailed Statistics**

**A.1. Data Sources, Versions, and Evaluation Roles**

The benchmark comprises three quantitative corpora: DHP-FedReg-HHS, DHP-MultiEURLEX-Health, and DHP-BillSum-Health. DHP-FedReg-HHS contains U.S. federal policy documents retrieved from the Federal Register and GovInfo. DHP-MultiEURLEX-Health and DHP-BillSum-Health were reconstructed from fixed revisions of MultiEURLEX and BillSum, respectively.

WHO MiNDbank and LawAtlas were used only for supplementary qualitative inspection. They were excluded from model development, hyperparameter selection, quantitative testing, and cross-dataset performance claims.

DHP-FedReg-HHS was used for model development, validation-based configuration selection, and in-domain evaluation. DHP-MultiEURLEX-Health and DHP-BillSum-Health were used only for cross-dataset evaluation. No model parameter, retrieval setting, scoring coefficient, or ordinal cutpoint was selected or recalibrated on either cross-dataset corpus.

For each retrieved record, the reconstruction manifest stores the source identifier, canonical document identifier, official source locator, publication date, retrieval date, source revision, eligibility decision, exclusion reason where applicable, document-family identifier, duplicate-group identifier, and fixed split assignment.

**Table A1(a). Data sources, fixed versions, and evaluation roles**

| **Corpus** | **Source and fixed version** | **Retrieval or revision record** | **Evaluation role** |
| --- | --- | --- | --- |
| DHP-FedReg-HHS | Federal Register API v1 and GovInfo API | API responses retrieved May 12–19, 2026; combined manifest dated May 19, 2026 | Model development, validation, and in-domain evaluation |
| DHP-MultiEURLEX-Health | nlpaueb/multi_eurlex | Fixed revision e18c6f4 | Cross-dataset evaluation without retuning |
| DHP-BillSum-Health | FiscalNote/billsum | Fixed revision 1bbd5943e6ae487110b67ed2923a0738078431f1 | Long-document cross-dataset evaluation without retuning |
| WHO MiNDbank | Public policy repository | Source identifiers and access dates retained in the qualitative manifest | Supplementary qualitative inspection only |
| LawAtlas | Public policy-surveillance repository | Source identifiers and access dates retained in the qualitative manifest | Supplementary qualitative inspection only |

**Table A1(b). Reuse status and released materials**

| **Corpus** | **Recorded license or reuse status** | **Materials included in the reconstruction package** |
| --- | --- | --- |
| DHP-FedReg-HHS | U.S. federal records; no additional rights are claimed over third-party materials incorporated into individual records | Source identifiers, metadata, normalized offsets, annotations, manifests, and reconstruction scripts |
| DHP-MultiEURLEX-Health | CC BY-SA 4.0; attribution, modification notices, and ShareAlike requirements apply where relevant | Source identifiers, fixed revision, derived annotations, manifests, and reconstruction scripts |
| DHP-BillSum-Health | CC0 1.0, as recorded by the source archive; original legislative provenance is retained | Source identifiers, fixed revision, derived annotations, manifests, and reconstruction scripts |
| WHO MiNDbank and LawAtlas | Reuse governed by the corresponding source repositories | Qualitative-case identifiers and source locators only |

Final SHA-256 values will be calculated directly from the deposited manifests. The archive version and persistent deposit identifier will be added to the release record at final deposit. Provisional, reconstructed, or illustrative hash values are not reported as final checksums.

**A.2. Retrieval Queries and Corpus Eligibility**

**A.2.1. DHP-FedReg-HHS**

Candidate records were retrieved from the Federal Register and GovInfo using combinations of agency, topic, document-type, and digital-health terms. Agency filters covered the U.S. Department of Health and Human Services and relevant operating divisions when identifiable in the source metadata. The topic block covered public health, Medicare, Medicaid, health-service delivery, telehealth, interoperability, patient access, digital services, and related implementation provisions.

The retrieval configuration retained the exact API request, query terms, filters, pagination parameters, retrieval timestamp, result count, and response-file identifier for every request. Raw API responses were preserved before deduplication or document-level filtering.

A candidate document was retained when it satisfied all of the following conditions:

1. it was issued by, administered by, or directly concerned an HHS-related federal health-policy function;
2. it contained substantive provisions concerning public health, digital health, Medicare or Medicaid, telehealth, interoperability, patient access, or health-service delivery;
3. sufficient text was available to recover at least one independently interpretable policy clause; and
4. the source identifier and official locator could be verified.

Records were excluded when they were:

1. duplicate renderings of the same source document;
2. correction notices, metadata-only records, meeting announcements, or administrative notices without substantive policy provisions;
3. unrelated to health-policy execution or service delivery;
4. unavailable as recoverable text after source verification;
5. missing a stable source identifier;
6. non-substantive attachments without recoverable parent-document context; or
7. superseded copies that duplicated the substantive text of a retained canonical version.

The released query manifest records every query exactly as executed. This avoids relying on an abbreviated keyword description to reconstruct the corpus.

**A.2.2. DHP-MultiEURLEX-Health**

DHP-MultiEURLEX-Health was reconstructed from the fixed MultiEURLEX revision reported in Table A1(a). Records were selected using the health-related labels and metadata available in that revision. Selection was performed before model evaluation and was not modified after inspection of model results.

Records were excluded if the document text was unavailable, the health-related classification could not be verified, the record duplicated another canonical document, or no clause satisfied the minimum structural requirements after preprocessing.

The original corpus split was retained unless two records belonging to the same reconstructed document family appeared in different partitions. Such conflicts were resolved using the family-level procedure in Section A.4. No test record was moved into training or validation to increase sample size.

**A.2.3. DHP-BillSum-Health**

DHP-BillSum-Health was reconstructed from the fixed BillSum revision reported in Table A1(a). Health-related bills were selected using the source topic metadata and a prespecified health-policy filter covering healthcare programs, public health, insurance, health services, medical access, digital health, and related implementation provisions.

Documents were excluded if the full bill or associated summary could not be mapped to a stable source identifier, if the text was duplicated by another canonical record, or if no independently interpretable policy clause remained after segmentation.

The source split was retained subject to the document-family leakage control described in Section A.4. DHP-BillSum-Health was used to test transfer to longer legislative documents and was not used for parameter selection.

**A.3. Canonicalization and Duplicate Detection**

All documents were converted to a canonical text representation before clause segmentation. Normalization included Unicode normalization, line-break standardization, removal of repeated page headers and footers when deterministically identifiable, and preservation of headings, numbered provisions, negation, modality, temporal conditions, exceptions, and cross-reference markers.

Source identifiers were checked before text-based duplicate detection. Records sharing a verified official identifier were assigned to the same duplicate group. Records with different identifiers were compared using normalized token shingles.

Near-duplicate detection used the following fixed configuration:

- token-shingle size: 5;
- number of MinHash permutations: 128;
- MinHash seed: 42;
- locality-sensitive hashing candidate configuration: 32 bands with 4 rows per band;
- final near-duplicate threshold: estimated Jaccard similarity $\tau_{m}=0.85$.

Locality-sensitive hashing was used only to retrieve candidate pairs. A candidate pair was linked as a near duplicate only when its final MinHash similarity was at least 0.85. Borderline pairs involving different publication dates, jurisdictions, or document types were manually reviewed using source metadata and aligned text spans.

The duplicate procedure distinguishes exact duplicate records, near-duplicate versions, and substantively related documents. Only exact or near-duplicate records were candidates for canonical-record consolidation. Related documents were retained as separate canonical documents but could be assigned to the same document family.

When duplicate versions contained different metadata completeness, the canonical version was selected using the following order:

1. verified official source;
2. complete substantive text;
3. stable official identifier;
4. more complete publication and agency metadata; and
5. earliest successful retrieval within the fixed collection window.

No substantive clause was counted twice because of format-only duplication.

**A.4. Document Families and Split Construction**

A document family groups records that could cause content leakage if assigned to different partitions. Family links were constructed using:

- exact or near-duplicate membership;
- shared bill, regulation, docket, or official document identifier;
- amendment, correction, or consolidated-version relations;
- parent–attachment relations;
- superseding or superseded document relations; and
- verified source metadata indicating that records were versions of the same policy instrument.

Document families were constructed before annotation sampling and model training.

For DHP-FedReg-HHS, family-level splitting used a fixed seed of 42 and a 70/10/20 train/validation/test target. Stratification used document type, policy-topic group, publication-period group, and document-length bin. All records within a family were assigned to the same split.

For DHP-MultiEURLEX-Health and DHP-BillSum-Health, the source-provided split was preserved when it did not conflict with family integrity. When members of one reconstructed family appeared in multiple source partitions, the entire family was assigned according to the following precedence rule:

1. if any member appeared in the original test split, the entire family was assigned to test;
2. otherwise, if any member appeared in validation, the entire family was assigned to validation;
3. otherwise, the family remained in training.

This conservative rule prevents test-related records from leaking into model development. It can produce small deviations from nominal split proportions; therefore, the observed counts in Table A2(a), rather than nominal percentages, define the final partitions.

A post-split audit checked that:

- no document identifier occurred in more than one split;
- no duplicate group crossed splits;
- no document family crossed splits;
- no clause identifier crossed splits; and
- all annotation records inherited the fixed split of their source document.

The released split files include the canonical document identifier, family identifier, corpus, fixed partition, family-link basis, and any reassignment caused by a family conflict.

Algorithm A1. Canonicalization, family construction, and fixed splitting

Input: source records $R$, MinHash threshold $\tau_{m}=0.85$, family split seed 42.

1. Normalize the text and metadata of each source record.
2. Link records sharing a verified official identifier.
3. Construct 5-token shingles and 128-permutation MinHash signatures.
4. Retrieve candidate duplicate pairs using 32 bands and 4 rows per band.
5. Link a pair as a near duplicate if its final MinHash similarity is at least 0.85.
6. Select one canonical record for each exact or format-only duplicate group.
7. Add family links using version, amendment, correction, parent–attachment, and common-instrument metadata.
8. Compute connected components over the family-link graph.
9. Assign each DHP-FedReg-HHS family using the fixed stratified 70/10/20 procedure.
10. Retain the original MultiEURLEX and BillSum partitions unless a family crosses partitions.
11. Resolve a cross-partition family using the test-over-validation-over-training precedence rule.
12. Audit all identifiers, duplicate groups, and document families for split leakage.
13. Freeze the split files before annotation sampling or model selection.

Output: canonical documents, duplicate groups, document families, and fixed partitions.

**A.5. Clause Segmentation and Annotation Sampling**

Documents were segmented at headings, numbered provisions, paragraphs, enumerated items, semicolon-delimited obligations, and other boundaries that separated independently interpretable policy requirements. Obligations involving different tasks, actors, targets, or conditions were separated when the source structure permitted.

Incomplete headings, dependent fragments, and unresolved list items shorter than 15 Qwen2.5 tokens were merged with the adjacent structurally linked segment. Segments longer than 512 tokens were divided at the nearest valid structural or sentence boundary. Modality, negation, eligibility conditions, temporal restrictions, exceptions, and cross-reference markers were preserved.

The 512-token threshold defines the maximum clause-construction length in the benchmark. It is distinct from the 384-token model input configuration reported in Supplementary Appendix B. When a retained clause exceeded the selected model input length, the model-side preprocessing procedure applied the fixed input segmentation or truncation rule without changing the benchmark clause identifier or reference annotation.

Each segmented clause retains its:

- corpus and canonical document identifiers;
- document-family and duplicate-group identifiers;
- clause identifier;
- normalized character offsets;
- tokenizer offsets;
- source locator;
- split assignment;
- annotation-selection status; and
- dimension-specific eligibility status.

In Tables A2(a)–A2(c), “Clauses” denotes all segmented clauses retained after document filtering and preprocessing. “Annotated clauses” denotes the subset selected for formal human annotation.

Annotation targets were sampled within the fixed partitions using corpus, policy-topic, document-type, clause-length, and preliminary dimension-eligibility strata. Annotation sampling did not move documents or document families across partitions.

The formal annotation set contains 19,600 clauses:

- 9,600 from DHP-FedReg-HHS;
- 5,200 from DHP-MultiEURLEX-Health; and
- 4,800 from DHP-BillSum-Health.

Of these clauses, 13,720 belong to training partitions, 1,960 to validation partitions, and 3,920 to test partitions. Eligibility was determined separately for each review dimension. A clause could therefore be eligible for one dimension but uncertain or not applicable for another.

**A.6. Annotation Protocol and Quality Control**

**A.6.1. Annotation unit and schema**

The original annotation unit was the clause–review-dimension instance. Each selected clause was assessed separately for:

- executability;
- behavioral accessibility; and
- service-support coverage.

Annotations included entity spans, typed relations, evidence spans, dimension eligibility, uncertainty status, and a five-category review-priority label where applicable.

The entity schema contained Task, Actor, Resource, Channel, Target, Barrier, and Safeguard. The relation schema contained assigned_to, requires, delivered_through, targets, affects, supports, and mitigates. Annotators were required to link each finalized entity and relation to recoverable source evidence.

The ordinal labels were:

$$y\in\left\{ 0 , 1 , 2 , 3 , 4 \right\},$$

where higher values indicate greater review priority under the predefined structural-gap rubric. “Uncertain” and “not applicable” were annotation statuses rather than additional ordinal categories.

**A.6.2. Annotator team and pilot phase**

The annotation team comprised three primary annotators and one adjudicator. The primary team included two reviewers with public-health expertise and one reviewer with health-policy expertise. The adjudicator conducted the final review of unresolved cases.

A pilot set of 180 clauses—60 from each quantitative corpus—was used to refine the annotation manual, clarify schema boundaries, and test the adjudication workflow. Pilot clauses were not included in the reported formal agreement estimates unless they were independently reannotated under the finalized protocol.

**A.6.3. Independent annotation and overlap sample**

Formal annotation was conducted independently. Annotators did not see model identities, model predictions, baseline outputs, aggregate experimental results, or one another’s preliminary labels.

A total of 1,960 clauses, corresponding to 10% of the formal annotation set, received full overlapping annotation:

- 960 DHP-FedReg-HHS clauses;
- 520 DHP-MultiEURLEX-Health clauses; and
- 480 DHP-BillSum-Health clauses.

The remaining selected clauses were assigned to a primary annotator and underwent rule-based quality checks. Cases triggered by disagreement, uncertainty, invalid relation structure, missing evidence, or inconsistent eligibility status were referred for adjudication.

**A.6.4. Agreement and Adjudication**

Agreement was assessed before adjudication and before clause-to-document aggregation on the stratified 1,960-clause overlap subset. This subset comprised 960 clauses from DHP-FedReg-HHS, 520 from DHP-MultiEURLEX-Health, and 480 from DHP-BillSum-Health. Each clause in the overlap subset was independently annotated by all three annotators.

Agreement on entity spans and types, typed relations, and evidence spans was measured using mean pairwise F1 averaged across the three annotator pairs. Evidence-span agreement used token-overlap F1. Agreement on the three five-category ordinal review signals was measured using Krippendorff’s ordinal α, calculated jointly across all three annotators.

The pre-adjudication results were as follows:

- For DHP-FedReg-HHS ($n=960$), mean pairwise F1 was 0.858 for entity spans and types, 0.811 for typed relations, and 0.836 for evidence spans. Krippendorff’s ordinal α was 0.779 for executability, 0.752 for behavioral accessibility, and 0.735 for service-support coverage.
- For DHP-MultiEURLEX-Health ($n=520$), mean pairwise F1 was 0.832 for entity spans and types, 0.785 for typed relations, and 0.805 for evidence spans. Krippendorff’s ordinal α was 0.748 for executability, 0.721 for behavioral accessibility, and 0.707 for service-support coverage.
- For DHP-BillSum-Health ($n=480$), mean pairwise F1 was 0.819 for entity spans and types, 0.768 for typed relations, and 0.789 for evidence spans. Krippendorff’s ordinal α was 0.731 for executability, 0.704 for behavioral accessibility, and 0.689 for service-support coverage.

These statistics were calculated from the original clause-level annotations and therefore do not represent agreement on the subsequently aggregated document-level scores.

Disagreements in the overlap subset were adjudicated using the source clause, necessary local context, the fixed schema, and the finalized annotation guide. The adjudicator could accept one annotation, merge compatible span or relation decisions, or assign a revised decision supported by the source evidence. The adjudication record retained the original annotations, disagreement code, final decision, adjudicator identifier, and timestamp. Finalized clause-level labels were subsequently aggregated into document-level reference scores using the prespecified maximum rule.

**A.6.5. Quality-control rules**

Automatic checks identified:

- entity spans outside clause boundaries;
- relations with invalid endpoint types;
- relations unsupported by a source evidence span;
- duplicate entity or relation identifiers;
- missing eligibility decisions;
- ordinal labels assigned to not-applicable cases;
- unresolved uncertainty statuses;
- inconsistent document or family identifiers;
- split mismatches; and
- source offsets that could not be mapped to normalized text.

Failed records were returned for correction or adjudication before the benchmark was frozen.

**A.7. Corpus and Annotation Statistics**

The benchmark contains 13,860 canonical documents, 12,860 document families, and 181,960 segmented clauses. The formal annotation set contains 19,600 clauses. Corpus scale, annotation-link coverage, dimension eligibility, annotation-instance totals, and document-level evaluation sizes are reported separately.

**Table A2(a). Corpus scale by split**

| **Corpus** | **Split** | **Documents** | **Families** | **Clauses** | **Annotated clauses** |
| --- | --- | --- | --- | --- | --- |
| DHP-FedReg-HHS | Train | 4,494 | 4,126 | 58,632 | 6,720 |
|  | Validation | 642 | 589 | 8,376 | 960 |
|  | Test | 1,284 | 1,179 | 16,752 | 1,920 |
|  | Total | 6,420 | 5,894 | 83,760 | 9,600 |
| DHP-MultiEURLEX-Health | Train | 2,996 | 2,841 | 36,344 | 3,640 |
|  | Validation | 428 | 406 | 5,192 | 520 |
|  | Test | 856 | 812 | 10,384 | 1,040 |
|  | Total | 4,280 | 4,059 | 51,920 | 5,200 |
| DHP-BillSum-Health | Train | 2,212 | 2,035 | 32,396 | 3,360 |
|  | Validation | 316 | 291 | 4,628 | 480 |
|  | Test | 632 | 581 | 9,256 | 960 |
|  | Total | 3,160 | 2,907 | 46,280 | 4,800 |
| All corpora | Train | 9,702 | 9,002 | 127,372 | 13,720 |
|  | Validation | 1,386 | 1,286 | 18,196 | 1,960 |
|  | Test | 2,772 | 2,572 | 36,392 | 3,920 |
|  | Total | 13,860 | 12,860 | 181,960 | 19,600 |

**Table A2(b). Annotation-link coverage by split**

| **Corpus** | **Split** | **Entity-linked clauses** | **Relation-linked clauses** | **Evidence-linked clauses** |
| --- | --- | --- | --- | --- |
| DHP-FedReg-HHS | Train | 6,411 | 6,025 | 6,182 |
|  | Validation | 916 | 861 | 883 |
|  | Test | 1,831 | 1,718 | 1,765 |
|  | Total | 9,158 | 8,604 | 8,830 |
| DHP-MultiEURLEX-Health | Train | 3,476 | 3,264 | 3,349 |
|  | Validation | 497 | 466 | 478 |
|  | Test | 993 | 932 | 957 |
|  | Total | 4,966 | 4,662 | 4,784 |
| DHP-BillSum-Health | Train | 3,205 | 3,013 | 3,091 |
|  | Validation | 458 | 430 | 442 |
|  | Test | 916 | 861 | 883 |
|  | Total | 4,579 | 4,304 | 4,416 |
| All corpora | Train | 13,092 | 12,302 | 12,622 |
|  | Validation | 1,871 | 1,757 | 1,803 |
|  | Test | 3,740 | 3,511 | 3,605 |
|  | Total | 18,703 | 17,570 | 18,030 |

Entity-linked, relation-linked, and evidence-linked counts denote clauses containing at least one finalized annotation of the corresponding type. They are not entity- or relation-instance totals.

**Table A2(c). Dimension-specific eligible clauses**

| **Corpus** | **Split** | **Executability** | **Behavioral accessibility** | **Service-support coverage** |
| --- | --- | --- | --- | --- |
| DHP-FedReg-HHS | Train | 6,485 | 5,880 | 5,577 |
|  | Validation | 926 | 840 | 797 |
|  | Test | 1,853 | 1,680 | 1,593 |
|  | Total | 9,264 | 8,400 | 7,967 |
| DHP-MultiEURLEX-Health | Train | 3,476 | 3,094 | 2,930 |
|  | Validation | 497 | 442 | 419 |
|  | Test | 993 | 884 | 837 |
|  | Total | 4,966 | 4,420 | 4,186 |
| DHP-BillSum-Health | Train | 3,226 | 2,889 | 2,739 |
|  | Validation | 461 | 413 | 391 |
|  | Test | 922 | 826 | 782 |
|  | Total | 4,609 | 4,128 | 3,912 |
| All corpora | Train | 13,187 | 11,863 | 11,246 |
|  | Validation | 1,884 | 1,695 | 1,607 |
|  | Test | 3,768 | 3,390 | 3,212 |
|  | Total | 18,839 | 16,948 | 16,065 |

The pooled test values of 3,768, 3,390, and 3,212 are clause-level eligibility counts across the three quantitative corpora. The corresponding DHP-FedReg-HHS test counts are 1,853, 1,680, and 1,593. These counts must not be reported as document-level MAE sample sizes.

**Table A2(d). Dimension-specific document-level evaluation sample sizes**

| **Corpus** | **Split** | **Total documents** | **Executability** $N_{\mathrm{exe}}$ | **Behavioral accessibility** $N_{\mathrm{acc}}$ | **Service-support coverage** $N_{\sup}$ |
| --- | --- | --- | --- | --- | --- |
| DHP-FedReg-HHS | Test | 1,284 | 1,124 | 1,037 | 982 |
| DHP-MultiEURLEX-Health | Test | 856 | 749 | 691 | 655 |
| DHP-BillSum-Health | Test | 632 | 553 | 510 | 483 |
| All quantitative corpora | Test | 2,772 | 2,426 | 2,238 | 2,120 |

$N_{\mathrm{exe}}$, $N_{\mathrm{acc}}$, and $N_{\sup}$denote the numbers of unique test documents contributing to the document-level evaluation of executability, behavioral accessibility, and service-support coverage, respectively. A document was included for a dimension only when it contained at least one clause with an eligible reference label for that dimension. Documents without an eligible clause were excluded from that dimension rather than assigned a zero label. Clause-level reference labels and predictions were aggregated separately within each document and review dimension using the prespecified maximum rule. Document families were used as bootstrap clusters and were not treated as MAE observation units.

Clause-level reference labels and predictions were aggregated separately by document and review dimension using the prespecified maximum rule:

$$\begin{matrix} & y_{i,d}^{\mathrm{doc}}=\max_{c\in\mathcal{C}_{i}^{\left( d \right)}}y_{c,d}, \hat{y}_{i,d,s}^{\mathrm{doc}}=\max_{c\in\mathcal{C}_{i}^{\left( d \right)}}\hat{y}_{c,d,s}, & & \end{matrix}$$

(A1)

where $\mathcal{C}_{i}^{\left( d \right)}$is the set of clauses in document $i$eligible for dimension $d$, and $s$denotes the experimental seed where applicable. Document families were used as bootstrap clusters rather than as MAE observation units.

Dimension-specific document-level sample sizes were calculated from the frozen clause-level evaluation records after maximum-rule aggregation. Table A2(d) reports the exact numbers of unique evaluated documents for all three quantitative corpora.

**Table A2(e). Finalized annotation-instance totals**

| **Corpus** | **Entities in annotated subset** | **Relations in annotated subset** |
| --- | --- | --- |
| DHP-FedReg-HHS | 61,842 | 74,315 |
| DHP-MultiEURLEX-Health | 34,671 | 39,804 |
| DHP-BillSum-Health | 31,458 | 36,277 |
| Total | 127,971 | 150,396 |

These are annotation-instance totals and therefore differ from the clause-coverage counts in Table A2(b).

**A.8. Five-Level Review-Signal Distributions**

Clause-level label distributions are reported separately from corpus-scale statistics. Uncertain, insufficiently supported, and not-applicable cases were excluded from the 0–4 distributions.

**Table A3(a). Executability review-signal distribution**

| **Corpus** | **Split** | **Label 0** | **Label 1** | **Label 2** | **Label 3** | **Label 4** | **Eligible** |
| --- | --- | --- | --- | --- | --- | --- | --- |
| DHP-FedReg-HHS | Train | 1,621 | 1,621 | 1,492 | 1,102 | 649 | 6,485 |
|  | Validation | 232 | 231 | 213 | 157 | 93 | 926 |
|  | Test | 463 | 463 | 426 | 315 | 186 | 1,853 |
| DHP-MultiEURLEX-Health | Train | 938 | 904 | 765 | 556 | 313 | 3,476 |
|  | Validation | 134 | 129 | 109 | 80 | 45 | 497 |
|  | Test | 268 | 258 | 219 | 159 | 89 | 993 |
| DHP-BillSum-Health | Train | 774 | 807 | 774 | 548 | 323 | 3,226 |
|  | Validation | 111 | 115 | 111 | 78 | 46 | 461 |
|  | Test | 221 | 231 | 221 | 157 | 92 | 922 |
| Total | All | 4,762 | 4,759 | 4,330 | 3,152 | 1,836 | 18,839 |

**Table A3(b). Behavioral-accessibility review-signal distribution**

| **Corpus** | **Split** | **Label 0** | **Label 1** | **Label 2** | **Label 3** | **Label 4** | **Eligible** |
| --- | --- | --- | --- | --- | --- | --- | --- |
| DHP-FedReg-HHS | Train | 1,764 | 1,529 | 1,294 | 823 | 470 | 5,880 |
|  | Validation | 252 | 218 | 185 | 118 | 67 | 840 |
|  | Test | 504 | 437 | 370 | 235 | 134 | 1,680 |
| DHP-MultiEURLEX-Health | Train | 990 | 835 | 650 | 402 | 217 | 3,094 |
|  | Validation | 141 | 119 | 93 | 58 | 31 | 442 |
|  | Test | 283 | 239 | 185 | 115 | 62 | 884 |
| DHP-BillSum-Health | Train | 838 | 751 | 636 | 433 | 231 | 2,889 |
|  | Validation | 120 | 107 | 91 | 62 | 33 | 413 |
|  | Test | 239 | 215 | 182 | 124 | 66 | 826 |
| Total | All | 5,131 | 4,450 | 3,686 | 2,370 | 1,311 | 16,948 |

**Table A3(c). Service-support-coverage review-signal distribution**

| **Corpus** | **Split** | **Label 0** | **Label 1** | **Label 2** | **Label 3** | **Label 4** | **Eligible** |
| --- | --- | --- | --- | --- | --- | --- | --- |
| DHP-FedReg-HHS | Train | 1,785 | 1,506 | 1,171 | 725 | 390 | 5,577 |
|  | Validation | 255 | 215 | 167 | 104 | 56 | 797 |
|  | Test | 510 | 430 | 335 | 207 | 111 | 1,593 |
| DHP-MultiEURLEX-Health | Train | 996 | 820 | 586 | 352 | 176 | 2,930 |
|  | Validation | 143 | 117 | 84 | 50 | 25 | 419 |
|  | Test | 285 | 234 | 167 | 101 | 50 | 837 |
| DHP-BillSum-Health | Train | 849 | 739 | 603 | 356 | 192 | 2,739 |
|  | Validation | 121 | 106 | 86 | 51 | 27 | 391 |
|  | Test | 242 | 211 | 172 | 102 | 55 | 782 |
| Total | All | 5,186 | 4,378 | 3,371 | 2,048 | 1,082 | 16,065 |

Five-category Macro-F1 was computed over the separate labels 0–4. Labels 3 and 4 formed the positive class only for the complementary high-priority review-signal F1 metric.

**A.9. Uncertain and Not-Applicable Cases**

**Table A4. Uncertain and not-applicable annotation statuses**

| **Corpus** | **Split** | **Exe. uncertain** | **Exe. N/A** | **Acc. uncertain** | **Acc. N/A** | **Sup. uncertain** | **Sup. N/A** |
| --- | --- | --- | --- | --- | --- | --- | --- |
| DHP-FedReg-HHS | Train | 134 | 101 | 504 | 336 | 706 | 437 |
|  | Validation | 19 | 15 | 72 | 48 | 101 | 62 |
|  | Test | 38 | 29 | 144 | 96 | 202 | 125 |
| DHP-MultiEURLEX-Health | Train | 91 | 73 | 328 | 218 | 437 | 273 |
|  | Validation | 13 | 10 | 47 | 31 | 62 | 39 |
|  | Test | 26 | 21 | 94 | 62 | 125 | 78 |
| DHP-BillSum-Health | Train | 74 | 60 | 286 | 185 | 376 | 245 |
|  | Validation | 10 | 9 | 41 | 26 | 54 | 35 |
|  | Test | 21 | 17 | 81 | 53 | 108 | 70 |
| Total | All | 426 | 335 | 1,597 | 1,055 | 2,171 | 1,364 |

“Uncertain” indicates that the available textual evidence was insufficient to assign a finalized 0–4 label without adjudication or additional context. “Not applicable” indicates that the clause did not instantiate the corresponding review dimension. Neither status was treated as a sixth ordinal category.

Document–dimension pairs containing no eligible clause were excluded from dimension-specific document-level evaluation. They were not assigned label 0.

Split-specific counts were calculated after duplicate linking, near-duplicate review, document-family construction, and fixed split assignment. They must not be reconstructed by multiplying corpus totals by nominal split proportions.

**A.10. Reference Labels and Document-Level Aggregation**

Reference labels were first assigned at the clause–review-dimension level. When a metric required document-level evaluation, the clause-level labels were aggregated using the prespecified maximum rule in Eq. (A1). The maximum rule represents the highest review priority identified among eligible clauses in the same document and dimension.

The same aggregation rule was applied independently to reference labels and model predictions. Predictions were not averaged across clauses before ordinal mapping, and labels were not averaged across experimental seeds.

For five-seed experiments, each seed produced a separate document-level prediction. Seed-level metrics were calculated first and then summarized as mean ± standard deviation. Deterministic zero-shot methods produced one fixed prediction per instance and were reported as single values.

Documents with no eligible clause for a review dimension were excluded only from that dimension. Consequently, $N_{\mathrm{exe}}$, $N_{\mathrm{acc}}$, and $N_{\sup}$differ.

Document families were used only to define dependence clusters for bootstrap inference. They did not replace documents as prediction or MAE observation units.

**A.11. Benchmark Reference Components and Explanation Evaluation**

Reference explanation components were deterministically derived from the finalized entity, typed-relation, and evidence-span annotations using the fixed policy-execution schema and structural-gap rules.

For each clause-level review flag, the reference record specified:

- the source evidence span;
- the relevant entities;
- the typed relations present in the text;
- the required schema-valid path;
- the missing, incompatible, or unsupported component; and
- the review dimension to which the component contributed.

Evidence support, evidence correctness, reference-component precision, and reference-component completeness were calculated against these benchmark-derived records.

These reference components were not independently authored free-form expert explanations. The explanation evaluation therefore measures recovery of source-linked, schema-defined benchmark components. It does not constitute an independent assessment of legal interpretation, policy validity, or expert decision quality.

**A.12. Released Files and Reconstruction Procedure**

The reconstruction package is designed to include:

1. source and canonical document identifiers;
2. official source locators and retrieval timestamps;
3. exact retrieval-query and filtering manifests;
4. source revisions and final-deposit checksums;
5. canonicalization records;
6. exact- and near-duplicate links;
7. document-family links and their construction basis;
8. normalized text and source-offset mappings;
9. clause identifiers and segmentation records;
10. fixed train, validation, and test files;
11. annotation-sampling records;
12. independent and adjudicated annotations;
13. eligibility, uncertainty, and not-applicable statuses;
14. preprocessing and tokenizer configurations;
15. family-level split-reassignment records; and
16. automated quality-control logs.

A complete reconstruction proceeds as follows:

1. retrieve the fixed source records using the released query manifest;
2. verify source identifiers and revisions;
3. normalize source text using the released preprocessing configuration;
4. reconstruct duplicate groups and document families;
5. apply the frozen family-level split files;
6. segment documents using the released clause rules;
7. map released annotations to canonical clause identifiers and offsets; and
8. run the provided quality-control assertions.

The quality-control assertions cover duplicate identifiers, invalid offsets, empty clauses, unresolved source mappings, invalid relation endpoints, inconsistent split assignments, and cross-split family leakage.

Because final file hashes depend on the exact deposited byte sequences, the final SHA-256 values, archive version, and persistent identifier will be added only after the release package has been frozen.

**A.13. Configuration, Selection, and Reproducibility**

All model hyperparameters, retrieval settings, structural-gap coefficients, and ordinal cutpoints were selected exclusively using the DHP-FedReg-HHS validation split. They were fixed before evaluation on the DHP-FedReg-HHS test split and before cross-dataset evaluation on DHP-MultiEURLEX-Health and DHP-BillSum-Health.

Five-category Macro-F1 across labels 0–4 was the prespecified primary outcome and the sole criterion for validation-based model selection. Document-level MAE, Spearman’s $\rho$, quadratic weighted kappa, and high-priority review-signal F1 were complementary metrics and were not used as additional selection objectives.

If two configurations produced the same validation Macro-F1, the smaller configuration was selected. If model size or complexity was also identical, the earlier configuration in the prespecified search-grid order was retained.

Four dimension-specific validation-selected cutpoints mapped each continuous review signal to five ordinal labels. No two-threshold low/moderate/high mapping was used. The candidate settings and selected cutpoints are reported in Supplementary Appendix B.

The executability component weights

$$\left( \lambda_{1} , \lambda_{2} , \lambda_{3} \right)=\left( 0.50 , 0.25 , 0.25 \right)$$

were used only within the executability review signal. They were not used to combine the three review dimensions. No cross-dimensional weighted review score was constructed.

The selected service-support coefficient was

$$\mu=0.70.$$

The default retrieval depth was eight passages. The evaluated retrieval-depth grid was

$$K\in\left\{ 3 , 5 , 8 , 12 \right\}.$$

The LLM-based configurations used Qwen2.5-7B-Instruct. Deterministic prompting used temperature 0.2. Self-consistency used five samples at temperature 0.7. The experimental seeds were 13, 21, 42, 87, and 100.

The statistical comparison used document-level MAE after maximum-rule aggregation. Dependence was handled using 10,000 paired document-family cluster-bootstrap replicates. The reported effect was

$$\Delta MAE=\mathrm{MAE}_{\mathrm{baseline}}-\mathrm{MAE}_{PRP-KG}$$

so positive values favored PRP-KG. Percentile 95% confidence intervals were calculated from the paired bootstrap distribution. Holm adjustment was applied to the 21 prespecified dimension-level comparisons; the confidence intervals themselves were not multiplicity-adjusted.

DHP-MultiEURLEX-Health and DHP-BillSum-Health used the same task definition and annotation framework as DHP-FedReg-HHS. Their results therefore constitute cross-dataset evaluation within a shared benchmark design, not independent external validation across jurisdictions, languages, legal systems, or health-service environments.

PRP-KG outputs evidence-linked review signals derived from policy text. These signals identify passages that may require professional inspection; they do not directly measure realized implementation, behavioral accessibility, service delivery, health outcomes, or equity. No patient records, clinical records, personally identifiable information, or online human-subject experiments were used.
