# Supplementary material for: A large language model-enhanced knowledge graph framework for text-implied public health policy gap screening: digital health executability, behavioral accessibility, and service-support coverage
Source: Front Public Health. 2026 Sep 7;14:1937335. doi: 10.3389/fpubh.2026.1937335 (PMC13590772; doi:10.3389/fpubh.2026.1937335)
Supplement: Supplementary file 2 [file Supplementary_file_2.docx]

**Supplementary Appendix B**

**Hyperparameter Selection, Review-Signal Scoring, and Statistical Reproducibility**

**B.1 Validation and Repeated-Run Protocol**

Five-category Macro-F1 at the eligible clause–review-dimension level was the sole criterion for selecting model configurations and dimension-specific ordinal cutpoints. For each stochastic candidate configuration, selection was based on the mean validation Macro-F1 across the five predefined runs. One common set of four ordered cutpoints per dimension was estimated exclusively from the DHP-FedReg-HHS validation results and was applied unchanged across the five runs. MAE, Spearman’s $\rho$, QWK, and high-priority F1 were complementary metrics and were not used for configuration or cutpoint selection. Ties were resolved in favor of the smaller configuration; when configuration size was not applicable, the prespecified grid order was used. The selected configurations and cutpoints were fixed before test evaluation and reused without recalibration in the cross-dataset evaluations.

Experiments used five predefined seeds: 13, 21, 42, 87, and 100. For trainable models, the seeds controlled parameter initialization, training-data order, and stochastic optimization. LLM-Direct, LLM-CoT, the generative stages of the RAG and KG–LLM methods, and the constrained extraction stage of PRP-KG used do_sample=true with temperature 0.2. Their seeds therefore controlled sampled decoding. Within each run, LLM-Self-Consistency generated five samples per input using do_sample=true and temperature 0.7 and aggregated the resulting ordinal predictions by majority vote.¹

Rule-KG Scoring was deterministic conditional on its input graph. Each run nevertheless used the graph produced by the corresponding seed-specific information-extraction pipeline. Its reported variation therefore originated from upstream extraction and graph construction rather than from the fixed scoring rules. The relation schema, rule weights, scoring functions, and ordinal cutpoints remained unchanged across runs.

Results from pipelines containing stochastic training, sampled decoding, or seed-specific upstream extraction are reported as mean ± SD over the five predefined runs. A single value is reported only when the complete pipeline is seed-invariant. Greedy decoding, where applicable, is specified as do_sample=false, for which temperature is not applicable. Temperature 0.2 is therefore described as low-temperature sampled decoding rather than deterministic decoding.

**Table B1. Baseline implementations, input representations, and validation-search budgets.**

| **Group** | **Method** | **Implementation/source** | **Text or evidence input** | **Graph input** | **Maximum validation configurations*** | **Source of repeated-run variation** |
| --- | --- | --- | --- | --- | --- | --- |
| Traditional text | TF-IDF + LinearSVC | Scikit-learn TF-IDF and LinearSVC | Clause text; unigram and bigram TF-IDF | None | 12 | Seed-controlled solver fitting |
| Text encoder | BERT | Task-adapted BERT-base implementation following [38] | Clause text | None | 18 | Initialization, data order, and optimization |
| Text encoder | LEGAL-BERT | Task-adapted implementation following [39] | Clause text | None | 18 | Initialization, data order, and optimization |
| Long-document encoder | Longformer | Task-adapted Longformer-base implementation following [40] | Document-level clause sequence | None | 18 | Initialization, data order, and optimization |
| LLM prompting | LLM-Direct | Qwen2.5-7B-Instruct [49]; fixed direct prompt | Clause text and task definition | None | 1 | Sampled decoding |
| LLM prompting | LLM-CoT | Qwen2.5-7B-Instruct [49]; fixed structured-reasoning prompt | Clause text and task definition | None | 1 | Sampled decoding |
| LLM prompting | LLM-Self-Consistency | Qwen2.5-7B-Instruct [49]; five samples and majority aggregation | Clause text and task definition | None | 1 | Five sampled outputs within each seeded run |
| Retrieval | BM25-RAG | BM25 retrieval with Qwen2.5-7B-Instruct [49] | Query clause and eight retrieved segments | Ranked text only | 9 | Sampled generation; retrieval index fixed |
| Retrieval | Dense-RAG | Dense retrieval with Qwen2.5-7B-Instruct [49] | Query clause and eight dense neighbors | Ranked text only | 12 | Sampled generation; retrieval index fixed |
| Evidence-aware retrieval | Text-RAG + Evidence | Dense retrieval with evidence-span-constrained Qwen2.5 prompting [49] | Clause, eight retrieved segments, and evidence spans | None | 12 | Sampled generation; retrieval index fixed |
| Rule graph | Rule-KG Scoring | Task-specific fixed rule scorer | Clause text and rule-matched elements | Seed-specific rule-derived typed graph | 1 | Upstream extraction and graph construction; scorer fixed |
| Graph neural model | R-GCN | Task-adapted reimplementation following [41] | Predicted node features | Shared typed comparison graph | 18 | Initialization, data order, and optimization |
| Graph neural model | HGT | Task-adapted reimplementation following [42] | Predicted node features | Shared typed comparison graph | 18 | Initialization, data order, and optimization |
| Graph-RAG | GraphRAG | Task-adapted graph-retrieval implementation† | Query clause and graph-linked evidence | Baseline-constructed graph | 12 | Graph construction and sampled generation |
| KG–LLM reasoning | LightPROF | Task-adapted reimplementation following [43] | Query clause and retrieved graph context | Baseline-specific predicted graph | 12 | Upstream graph construction and sampled generation |
| Evidence-path reasoning | EPERM | Task-adapted reimplementation following [44] | Query clause, evidence paths, and source spans | Baseline-specific path graph | 12 | Upstream extraction, graph construction, and stochastic inference |
| Heterogeneous reasoning | AtomR | Task-adapted reimplementation following [45] | Query clause and heterogeneous evidence | Baseline-specific heterogeneous graph | 12 | Upstream extraction, graph construction, and stochastic inference |
| Information extraction | BERT-CRF | Task-adapted BERT [38] with a CRF head | Clause tokens | None during extraction | 18 | Initialization, data order, and optimization |
| Information extraction | SpERT | Task-adapted reimplementation following [46] | Clause tokens and candidate spans | None during extraction | 18 | Initialization, data order, and optimization |
| Information extraction | CasRel | Task-adapted reimplementation following [47] | Clause tokens | None during extraction | 18 | Initialization, data order, and optimization |
| Information extraction | UIE | Task-adapted implementation following [48] with fixed schema prompts | Clause text and fixed schema | None during extraction | 18 | Fine-tuning initialization, data order, and optimization‡ |
| Proposed | PRP-KG | Qwen2.5-7B-Instruct [49] with constrained extraction, schema validation, and structural-gap scoring | Clause text, retrieved evidence, and source offsets | Three-layer policy graph | Actual search count§ | Sampled constrained extraction; validation and scoring fixed |

The reported number is the maximum number of complete, prespecified configurations actually evaluated on the DHP-FedReg-HHS validation split; it is not the Cartesian product of all candidate values. It excludes the five repeated runs and dimension-specific ordinal-cutpoint fitting. For stochastic candidate configurations, selection was based on mean five-run validation Macro-F1. The decoding settings of temperature 0.2 and the self-consistency setting of temperature 0.7 with five samples were prespecified and therefore counted as one configuration. Completely seed-invariant pipelines were reported as single values. All selected settings were frozen before test evaluation and reused without cross-dataset retuning.

**B.2. One-Factor Validation Sensitivity**

The relation-consistency threshold $\tau_{c}$, maximum number of feedback re-query rounds $R$, and retrieval depth $K$were varied separately while all other settings remained fixed. Table B2 reproduces Table 10 of the main manuscript so that the declared selection criterion can be audited directly.

**Table B2. One-factor parameter sensitivity on the DHP-FedReg-HHS validation split, mean ± SD over five runs.**

| **Parameter** | **Value** | **Validation Macro-F1 ↑** | **Relation F1 ↑** | **Graph consistency ↑** | **Five-category document-label MAE ↓** | **Runtime/document ↓** |
| --- | --- | --- | --- | --- | --- | --- |
| Consistency threshold $\tau_{c}$ | 0.60 | 0.712 ± 0.009 | 0.736 ± 0.018 | 0.808 ± 0.020 | 0.472 ± 0.021 | 31.7 ± 2.1 s |
|  | 0.70 | 0.749 ± 0.007 | 0.752 ± 0.016 | 0.839 ± 0.017 | 0.444 ± 0.018 | 34.2 ± 2.4 s |
|  | **0.75** | **0.761 ± 0.006** | 0.759 ± 0.015 | 0.846 ± 0.015 | 0.438 ± 0.017 | 35.6 ± 2.5 s |
|  | 0.80 | 0.754 ± 0.007 | 0.754 ± 0.016 | 0.852 ± 0.014 | 0.447 ± 0.018 | 38.8 ± 2.7 s |
|  | 0.85 | 0.742 ± 0.009 | 0.742 ± 0.018 | 0.858 ± 0.013 | 0.466 ± 0.020 | 41.5 ± 3.0 s |
| Maximum re-query rounds $R$ | 0 | 0.688 ± 0.011 | 0.713 ± 0.020 | 0.781 ± 0.022 | 0.503 ± 0.023 | 19.1 ± 1.8 s |
|  | 1 | 0.738 ± 0.008 | 0.741 ± 0.017 | 0.821 ± 0.019 | 0.461 ± 0.020 | 26.3 ± 2.2 s |
|  | **2** | **0.761 ± 0.006** | 0.759 ± 0.015 | 0.846 ± 0.015 | 0.438 ± 0.017 | 35.6 ± 2.5 s |
|  | 3 | 0.760 ± 0.006 | 0.763 ± 0.015 | 0.853 ± 0.014 | 0.436 ± 0.017 | 47.9 ± 3.6 s |
| Retrieved evidence top-$K$ | 3 | 0.724 ± 0.009 | 0.731 ± 0.018 | 0.823 ± 0.018 | 0.462 ± 0.020 | 29.7 ± 2.0 s |
|  | 5 | 0.747 ± 0.007 | 0.748 ± 0.017 | 0.837 ± 0.017 | 0.446 ± 0.018 | 32.4 ± 2.3 s |
|  | **8** | **0.761 ± 0.006** | 0.759 ± 0.015 | 0.846 ± 0.015 | 0.438 ± 0.017 | 35.6 ± 2.5 s |
|  | 12 | 0.760 ± 0.006 | 0.761 ± 0.015 | 0.849 ± 0.015 | 0.439 ± 0.018 | 42.8 ± 3.1 s |

Bold values identify the selected parameter settings. The threshold $\tau_{c}=0.75$achieved the highest validation Macro-F1 among the evaluated threshold settings. Although higher thresholds increased graph consistency, they did not improve the sole selection metric. Similarly, $R=3$and $K=12$each produced a slightly lower validation Macro-F1 than the selected configuration while increasing runtime. Relation F1, graph consistency, five-category document-label MAE, and runtime were used only as diagnostic measures.

**B.3. Type-Specific Weights and Uniform-Weight Comparison**

The review-signal formulation uses four type-specific weight vectors:

- $\omega_{b}$: relative weight of barrier category $b$;
- $\omega_{h}$: relative weight of accessibility-safeguard category $h$;
- $\nu_{s}$: relative weight of service-support category $s$; and
- $\pi_{g}$: relative review-priority weight of target-group category $g$.

Relative weights were restricted to $\left\{ 1.00 , 1.25 , 1.50 \right\}$, representing standard, elevated, and high rubric-defined review relevance. They are category-level scoring constants rather than instance-level confidence scores or estimates of prevalence, causal vulnerability, realized severity, clinical need, or population impact. Each applicable category is counted once per document–group pair; repeated mentions are retained only as evidence links.

The rubric-derived configuration was compared with a uniform-weight alternative while predictions, graphs, evidence links, scalar coefficients, applicable category sets, and aggregation rules were held fixed.

**Table B3. Weighting-scheme comparison on the DHP-FedReg-HHS validation split, mean ± SD over five runs.**

| **Weighting scheme** | **Validation Macro-F1 ↑** |
| --- | --- |
| Uniform | 0.748 ± 0.006 |
| Rubric-derived | 0.761 ± 0.006 |

The rubric-derived configuration was retained because it achieved the higher validation Macro-F1. The previous continuous-score “Overall MAE” column was removed because its reference values, formula, and statistical unit were not independently specified and could be confused with the five-category document-label MAE in Table B2.

**Table B4. Barrier-category weights.**

| **ID** | **Barrier category** | $\boldsymbol{\omega}_{\boldsymbol{b}}$ |
| --- | --- | --- |
| B1 | Online-only access requirement | 1.50 |
| B2 | Device or stable-connectivity dependence | 1.25 |
| B3 | Digital identity-authentication burden | 1.25 |
| B4 | Complex platform operation | 1.00 |
| B5 | Digital-literacy requirement | 1.25 |

**Table B5. Accessibility-safeguard weights.**

| **ID** | **Accessibility-safeguard category** | $\omega_{h}$ |
| --- | --- | --- |
| H1 | Offline service window | 1.50 |
| H2 | Telephone service channel | 1.25 |
| H3 | Community or on-site assistance | 1.25 |
| H4 | Proxy-application arrangement | 1.25 |
| H5 | Family-doctor or designated-person support | 1.00 |

**Table B6. Service-support-category weights.**

| **ID** | **Service-support category** | $\nu_{s}$ |
| --- | --- | --- |
| S1 | Dedicated service channel | 1.25 |
| S2 | Explicit implementation resource | 1.25 |
| S3 | Assisted service arrangement | 1.50 |
| S4 | Target-linked responsible actor | 1.00 |
| S5 | Follow-up, referral, or continuity provision | 1.00 |

**Table B7. Target-group review-priority weights.**

| **ID** | **Target-group category** | $\pi_{g}$ |
| --- | --- | --- |
| G1 | General population or unspecified beneficiaries | 1.00 |
| G2 | Older adults | 1.50 |
| G3 | People with disabilities | 1.50 |
| G4 | Low-income or publicly insured populations | 1.25 |
| G5 | Rural or connectivity-constrained populations | 1.25 |
| G6 | People with limited digital literacy | 1.50 |
| G7 | Caregivers or proxy users | 1.00 |

**B.4. Component-Specific Review-Signal Coefficients**

PRP-KG outputs executability, behavioral-accessibility, and service-support-coverage review signals separately. No cross-dimensional combined score is calculated.

For executability,

$$\begin{matrix} & R_{i}^{exe}=1-\left( \lambda_{1}S_{i}^{path}+\lambda_{2}S_{i}^{actor}+\lambda_{3}S_{i}^{res} \right), & & \end{matrix}$$

(B1)

where $S_{i}^{path}$, $S_{i}^{actor}$, and $S_{i}^{res}$denote normalized execution-path completeness, actor clarity, and resource support, respectively. The weights satisfy

$$\begin{matrix} & \lambda_{1}+\lambda_{2}+\lambda_{3}=1, \lambda_{j}\geq0, & & \end{matrix}$$

(B2)

and the search varied $\lambda_{1}$while setting

$$\begin{matrix} & \lambda_{2}=\lambda_{3}=\frac{1-\lambda_{1}}{2}. & & \end{matrix}$$

(B3)

The selected setting was

$$\left( \lambda_{1} , \lambda_{2} , \lambda_{3} \right)=\left( 0.50 , 0.25 , 0.25 \right).$$

(B4)

Within service-support coverage, $\mu$balances overall support insufficiency against maximum between-group deviation. The selected value was $\mu=0.70$.

**Table B8. Component-specific scoring coefficients.**

| **Coefficient** | **Review dimension** | **Candidate values** | **Selected value** |
| --- | --- | --- | --- |
| $\lambda_{1}$ | Executability | 0.30, 0.40, 0.50, 0.60, 0.70 | 0.50 |
| $\lambda_{2}$ | Executability | Derived using Eq. (B5) | 0.25 |
| $\lambda_{3}$ | Executability | Derived using Eq. (B5) | 0.25 |
| $\mu$ | Service-support coverage | 0.50, 0.60, 0.70, 0.80, 0.90 | 0.70 |

These coefficients operate only within their respective review dimensions. Higher scores indicate greater expert-review priority under the predefined schema rather than realized policy outcomes.

**B.5. Behavioral-Accessibility Components**

For document $i$and target group $g$, let $\mathcal{B}_{i}\left( g \right)$denote the applicable barrier categories. The normalized barrier score is

$$\begin{matrix} & B_{i}\left( g \right)=\frac{\sum_{b\in\mathcal{B}_{i}\left( g \right)} \omega_{b}\mathbb{I}\left[ \left( b , \text{affects} , g \right)\in\mathcal{E}_{i} \right]}{\sum_{b\in\mathcal{B}_{i}\left( g \right)} \omega_{b}+\epsilon}. & & \end{matrix}$$

(B5)

A barrier contributes only when a schema-valid, evidence-grounded Barrier $\underset{\to}{\text{affects}}$Target relation links it to the evaluated group. A digital channel, authentication requirement, or target-group label alone does not establish a behavioral barrier.

Let $\mathcal{H}_{i}^{acc}\left( g \right)$denote the applicable accessibility-safeguard categories. The normalized safeguard score is

$$\begin{matrix} & H_{i}^{acc}\left( g \right)=\frac{\sum_{h\in\mathcal{H}_{i}^{acc}\left( g \right)} \omega_{h}\mathbb{I}\left[ \left( h , \text{supports} , g \right)\in\mathcal{E}_{i}\text{ }\vee\text{ }\exists b\in\mathcal{B}_{i}\left( g \right):\left( h , \text{mitigates} , b \right)\in\mathcal{E}_{i} \right]}{\sum_{h\in\mathcal{H}_{i}^{acc}\left( g \right)} \omega_{h}+\epsilon}. & & \end{matrix}$$

(B6)

The group-level behavioral access-barrier signal is

$$\begin{matrix} & r_{i}^{acc}\left( g \right)=B_{i}\left( g \right)\left[ 1-H_{i}^{acc}\left( g \right) \right]. & & \end{matrix}$$

(B7)

This multiplicative form ensures that no access-barrier signal is generated when no text-supported barrier is linked to the target group. Repeated mentions of one category are counted once.

**B.6. Service-Support-Coverage Components**

Accessibility safeguards and general service-support provisions are represented by separate category sets. Let $\mathcal{P}_{i}^{sup}\left( g \right)$denote the applicable service-support categories for target group $g$. The normalized support score is

$$\begin{matrix} & S_{i}^{sup}\left( g \right)=\frac{\sum_{s\in\mathcal{P}_{i}^{sup}\left( g \right)} \nu_{s}\mathbb{I}\left[ \mathrm{linked}_{i}\left( s , g \right)=1 \right]}{\sum_{s\in\mathcal{P}_{i}^{sup}\left( g \right)} \nu_{s}+\epsilon}. & & \end{matrix}$$

(B8)

The indicator $\mathrm{linked}_{i}\left( s , g \right)$equals one only when category $s$is connected to group $g$through a prespecified schema-valid and evidence-grounded target-support structure. Eligible structures include Safeguard $\underset{\to}{\text{supports}}$Target or a Task-centered structure linking an applicable Resource or Channel to the same Task–Target unit through requires, delivered_through, and targets. A path is not counted merely because it contains a target-group node.

**B.7. Group-Weight Normalization and Document-Level Signals**

For behavioral accessibility,

$$\begin{matrix} & \tilde{\pi}_{ig}^{acc}=\frac{\pi_{g}}{\sum_{g^{'}\in\mathcal{G}_{i}^{acc}} \pi_{g^{'}}+\epsilon}, & & \end{matrix}$$

(B9)

and

$$\begin{matrix} & R_{i}^{acc}=\sum_{g\in\mathcal{G}_{i}^{acc}} \tilde{\pi}_{ig}^{acc}B_{i}\left( g \right)\left[ 1-H_{i}^{acc}\left( g \right) \right]. & & \end{matrix}$$

(B10)

For service-support coverage,

$$\begin{matrix} & \tilde{\pi}_{ig}^{sup}=\frac{\pi_{g}}{\sum_{g^{'}\in\mathcal{G}_{i}^{sup}} \pi_{g^{'}}+\epsilon}, & & \end{matrix}$$

(B11)

$$\begin{matrix} & \hat{S}_{i}=\sum_{g\in\mathcal{G}_{i}^{sup}} \tilde{\pi}_{ig}^{sup}S_{i}^{sup}\left( g \right), & & \end{matrix}$$

(B12)

and

$$\begin{matrix} & R_{i}^{sup}=\mu\left( 1-\hat{S}_{i} \right)+\left( 1-\mu\right)\max_{g\in\mathcal{G}_{i}^{sup}}\left| S_{i}^{sup}\left( g \right)-\hat{S}_{i} \right|. & & \end{matrix}$$

(B13)

The first term represents insufficient overall support coverage in the analyzed text. The second represents the largest deviation from the normalized weighted mean across applicable target groups. The symbol $R_{i}^{sup}$is used consistently; $R_{i}^{eq}$is not used.

Because all component scores and normalized weights lie in $\left[ 0 , 1 \right]$, $R_{i}^{acc}$and $R_{i}^{sup}$are bounded in $\left[ 0 , 1 \right]$. Neither signal directly measures realized accessibility, actual service provision, distributive equity, or population outcomes.

**B.8. Applicability and Missing-Value Handling**

Applicability was determined using a fixed category-to-dimension mapping established before test evaluation. Categories not applicable to a document–group pair were excluded from both the numerator and denominator:

$$\begin{matrix} & \mathcal{B}_{i}\left( g \right)=\emptyset\Rightarrow B_{i}\left( g \right)=\mathrm{NA}, & & \end{matrix}$$

(B14)

$$\begin{matrix} & \mathcal{H}_{i}^{acc}\left( g \right)=\emptyset\Rightarrow H_{i}^{acc}\left( g \right)=\mathrm{NA}, & & \end{matrix}$$

(B15)

$$\begin{matrix} & \mathcal{P}_{i}^{sup}\left( g \right)=\emptyset\Rightarrow S_{i}^{sup}\left( g \right)=\mathrm{NA}. & & \end{matrix}$$

(B16)

Not-applicable pairs were excluded from the corresponding aggregation rather than assigned zero. When an accessibility-safeguard category set was applicable but no valid safeguard relation was recovered, $H_{i}^{acc}\left( g \right)=0$. The numerical constant was fixed at $\epsilon={10}^{-8}$for stability; it does not replace the applicability rule.

**B.9. Scalar-Coefficient Sensitivity**

One scalar coefficient was varied at a time while predicted graphs, evidence links, type-specific weights, and validation records remained fixed. Validation Macro-F1 was the sole selection criterion; the previous continuous-score MAE values are not reported.

**Table B9. Sensitivity to** $\lambda_{1}$**, mean ± SD over five runs.**

| $\boldsymbol{\lambda}_{\boldsymbol{1}}$ | **Derived** $\boldsymbol{\lambda}_{\boldsymbol{2}}\boldsymbol{=}\boldsymbol{\lambda}_{\boldsymbol{3}}$ | **Validation Macro-F1 ↑** | **High-priority review-signal F1 ↑** |
| --- | --- | --- | --- |
| 0.30 | 0.35 | 0.750 ± 0.006 | 0.719 ± 0.008 |
| 0.40 | 0.30 | 0.756 ± 0.006 | 0.727 ± 0.007 |
| 0.50 | 0.25 | 0.761 ± 0.006 | 0.734 ± 0.007 |
| 0.60 | 0.20 | 0.757 ± 0.006 | 0.729 ± 0.008 |
| 0.70 | 0.15 | 0.749 ± 0.007 | 0.720 ± 0.009 |

**Table B10. Sensitivity to** $\mu$**, mean ± SD over five runs.**

| $\boldsymbol{\mu}$ | **Validation Macro-F1 ↑** | **High-priority review-signal F1 ↑** |
| --- | --- | --- |
| 0.50 | 0.755 ± 0.006 | 0.726 ± 0.008 |
| 0.60 | 0.758 ± 0.006 | 0.731 ± 0.007 |
| 0.70 | 0.761 ± 0.006 | 0.734 ± 0.007 |
| 0.80 | 0.759 ± 0.007 | 0.731 ± 0.008 |
| 0.90 | 0.754 ± 0.007 | 0.725 ± 0.009 |

Figure 9 in the main manuscript visualizes the one-factor sensitivity results reported in Tables B9 and B10. Panel (a) varies $\lambda_{1}$, with $\lambda_{2}=\lambda_{3}=\left( 1-\lambda_{1} \right)/2$, whereas Panel (b) varies $\mu$. All other scalar coefficients, type-specific category weights, predicted graphs, evidence links, and validation records remain fixed. Validation Macro-F1 is the sole selection criterion, and high-priority review-signal F1 is shown only as a complementary diagnostic metric.

**B.10. Model, Retrieval, and Graph Hyperparameters**

**Table B11. Model, retrieval, graph-construction, and optimization settings.**

| **Component** | **Hyperparameter** | **Candidate or prespecified values** | **Selected value** |
| --- | --- | --- | --- |
| Clause processing | Maximum clause-segment length | 256, 384, 512 tokens | 384 |
| Clause processing | Segment overlap | 32, 64, 96 tokens | 64 |
| Dense retrieval | Retrieved evidence segments $K$ | 3, 5, 8, 12 | 8 |
| Dense retrieval | Maximum total evidence length | 1024, 1536, 2048 tokens | 1536 |
| LLM prompting | Low-temperature sampled-decoding temperature | 0.2 (prespecified) | 0.2 |
| Self-consistency | Sampling temperature | 0.7 (prespecified) | 0.7 |
| Self-consistency | Samples per input | 5 (prespecified) | 5 |
| Graph validation | Relation-consistency threshold $\tau_{c}$ | 0.60, 0.70, 0.75, 0.80, 0.85 | 0.75 |
| Extraction | Minimum candidate-relation confidence | 0.50, 0.60, 0.70, 0.80 | 0.70 |
| Evidence grounding | Minimum span-overlap ratio | 0.40, 0.50, 0.60 | 0.50 |
| Evidence grounding | Maximum evidence-span length | 64, 96, 128 tokens | 96 |
| Feedback refinement | Maximum re-query rounds $R$ | 0, 1, 2, 3 | 2 |
| Explanation | Maximum explanation-path length | 3, 4, 5 hops | 4 |
| Neural optimization | Learning rate | $1\times{10}^{-5},2\times{10}^{-5},5\times{10}^{-5}$ | $2\times{10}^{-5}$ |
| Neural optimization | Batch size | 8, 16, 32 | 16 |
| Neural optimization | Weight decay | 0.00, 0.01, 0.05 | 0.01 |
| Neural optimization | Epochs | 3, 5, 8 | 5 |
| Neural optimization | Warm-up ratio | 0.05, 0.10, 0.15 | 0.10 |
| Neural optimization | Gradient-clipping norm | 0.5, 1.0, 2.0 | 1.0 |

The 384-token setting denotes the maximum length of each model-side clause segment, rather than the total LLM context length. It is distinct from the 512-token maximum used during benchmark clause construction in Supplementary Appendix A. Longer benchmark clauses were divided using the fixed 64-token-overlap rule without changing their benchmark identifiers or annotations. For retrieval-augmented configurations, retrieved evidence was separately limited to a total of 1,536 tokens.

All LLM-based configurations used Qwen2.5-7B-Instruct. LLM-Direct, LLM-CoT, and the generative RAG and KG–LLM configurations used low-temperature sampled decoding with do_sample=true and temperature 0.2. Self-consistency used temperature 0.7 with five samples per input. Accordingly, temperature 0.2 is not described as deterministic decoding. Five-run results used seeds 13, 21, 42, 87, and 100.

Searchable hyperparameters were selected only on the DHP-FedReg-HHS validation split using five-category Macro-F1. The decoding and self-consistency settings marked as prespecified were fixed before evaluation and were not selected from the test data. All reported settings were frozen for the DHP-FedReg-HHS test split and reused without cross-dataset retuning. Method-specific exceptions to the default neural-optimization settings are reported in Supplementary Table B1.

**B.11. Ordinal Cutpoint Selection**

For each review dimension, four cutpoints mapped the continuous signal to labels 0–4:

$$\begin{matrix} & \hat{y}_{d}\left( R \right)=\left\{ \begin{matrix} 0, & R<c_{d,1}, \\ 1, & c_{d,1}\leq R<c_{d,2}, \\ 2, & c_{d,2}\leq R<c_{d,3}, \\ 3, & c_{d,3}\leq R<c_{d,4}, \\ 4, & R\geq c_{d,4}. \end{matrix} \right. & & \end{matrix}$$

(B17)

Ordered quadruples were searched separately for each dimension on a 0.01 grid over $\left[ 0.10 , 0.90 \right]$, subject to

$$c_{d,1}<c_{d,2}<c_{d,3}<c_{d,4}.$$

**Table B12. Validation-selected ordinal cutpoints.**

| **Review dimension** | $\left( c_{1} , c_{2} , c_{3} , c_{4} \right)$ | **Selection criterion** |
| --- | --- | --- |
| Executability | (0.18, 0.34, 0.50, 0.66) | Five-category validation Macro-F1 |
| Behavioral accessibility | (0.16, 0.31, 0.47, 0.63) | Five-category validation Macro-F1 |
| Service-support coverage | (0.20, 0.36, 0.52, 0.68) | Five-category validation Macro-F1 |

The cutpoints were frozen before test evaluation and reused unchanged for DHP-MultiEURLEX-Health and DHP-BillSum-Health. Labels 3–4 formed the positive class only for the supplementary high-priority review-signal F1 metric.

**B.12. Robustness Perturbations**

Perturbations were generated once on the fixed DHP-FedReg-HHS robustness subset before model evaluation and were shared across all methods. No perturbed data were used for training, model selection, ordinal-cutpoint calibration, or retrieval-index construction.

**Table B13. Robustness perturbations.**

| **Perturbation** | **Settings** | **Fixed controls** |
| --- | --- | --- |
| OCR-style noise | Character deletion, insertion, or substitution at rates of 5%, 10%, and 15%; manifest seed 20250317 | Document and clause identifiers, reference labels, titles, and metadata retained |
| Meaning-preserving paraphrasing | mistralai/Mistral-7B-Instruct-v0.3; do_sample=false; temperature and top-$p$ not applicable; max_new_tokens=256 | Obligations, actors, target groups, conditions, modality, negation, quantities, dates, and relation directions preserved |
| Clause truncation | One contiguous span containing 20% of body-text tokens removed; manifest seed 20250317 | Document and clause identifiers and reference labels retained |

The paraphrasing model was distinct from the Qwen2.5-7B-Instruct backbone used by PRP-KG and was used only for perturbation generation. A generated paraphrase was rejected if it altered a named entity, numerical value or threshold, date, modality, negation, conditional scope, actor–action assignment, target group, or intended policy requirement. Failed outputs were regenerated once using the same fixed decoding configuration; clauses that still failed the preservation checks were excluded, and the retained proportion was recorded.

The perturbation manifest records the source-instance identifier, perturbation operation, manifest seed where applicable, input and output hashes, affected character or token offsets, evidence-span mappings, acceptance status, and exclusion reason. All methods were evaluated on identical accepted perturbation instances using frozen hyperparameters, retrieval indices, scoring rules, and ordinal cutpoints. Results across the five predefined model runs therefore reflect model-side stochasticity or seed-specific upstream extraction, not variation in the perturbation instances.

**B.13. Bootstrap Reproducibility**

**Table B14. Statistical settings.**

| **Field** | **Value** |
| --- | --- |
| Bootstrap replicates | 10,000 |
| MAE observation unit | Document |
| Dependence cluster | Document family |
| Bootstrap seed | 20250317 |
| Seed aggregation | Mean across five seeds within each replicate |
| Multiplicity adjustment | Holm across 21 dimension-level comparisons |
| Confidence interval | Unadjusted percentile 95% CI |

Clause-level reference labels and predictions were first aggregated separately by document and review dimension using the prespecified maximum rule. Documents without an eligible clause for a given dimension were excluded from that dimension rather than assigned label 0. Accordingly, documents—not document families—were the MAE observation units, whereas document families defined the dependence clusters used during resampling.

Document families were sampled with replacement. Within each replicate, the same sampled families were used for PRP-KG and the corresponding comparator. For comparator $b$and review dimension $d$, the effect was defined as

$$\begin{matrix} & \Delta\mathrm{MAE}_{b,d}=\mathrm{MAE}_{b,d}-\mathrm{MAE}_{\mathrm{PRP}\text{-}\mathrm{KG},d}. & & \end{matrix}$$

(B19)

Positive values therefore favor PRP-KG. Table 13 of the main manuscript reports unadjusted percentile 95% confidence intervals and finite-replicate bootstrap $p$-values. Holm adjustment was applied jointly to the 21 prespecified dimension-level comparisons. Overall effects were calculated as equal-weighted descriptive means across the three dimensions and were excluded from the Holm correction family.

The dimension-specific DHP-FedReg-HHS document-level sample sizes were

$$N_{\mathrm{exe}}=1,124, N_{\mathrm{acc}}=1,037, N_{\sup}=982.$$

**B.14. Alternative Structural-Gap Definitions**

Three prespecified alternatives were evaluated using the fixed predicted graph, evidence links, data partitions, and selected model configuration. They modified only the mapping from graph structure to review signals and were not used for model selection.

**Table B15. Alternative structural-gap definitions.**

| **Definition** | **Executability** | **Behavioral accessibility** | **Service-support coverage** | **Primary contrast** |
| --- | --- | --- | --- | --- |
| Strict binary gaps | Flag when any required Actor, Resource, Channel, or Target component is absent from a complete task path | Flag only when a valid Barrier–Target relation is present and no linked safeguard supports the target or mitigates the barrier | Flag an applicable group when no target-linked support category is present | Replaces graded components with present/absent decisions |
| Unweighted local deficits | Mean missing rate across applicable Task–Actor, Task–Resource, Task–Channel, and Task–Target relations | Proportion of matched barriers without an applicable safeguard | One minus the unweighted applicable support-category coverage, macro-averaged over groups | Removes rubric weights and complete-path weighting |
| Evidence-conservative gaps | Count a component only when its typed relation is schema-valid and evidence-grounded | Require accepted evidence for both the barrier or safeguard relation and the target link; unresolved pairs are omitted | Require every edge in the target-linked support structure to be evidence-grounded | Tightens evidence acceptance and separates unresolved cases from absence |

These alternatives are diagnostic definitions rather than separately tuned models. Not-applicable categories remain excluded from denominators and are never converted into structural zeros.

**B.15. Explanation-Quality Metric Definitions**

Explanation-quality metrics were computed automatically against evidence-linked reference components derived from finalized entity, relation, and evidence-span annotations. They are not ratings from an independent human-expert study.

**Table B16. Explanation-quality metrics.**

| **Metric** | **Statistical unit** | **Definition** |
| --- | --- | --- |
| Evidence support | Clause-level review-flag instance | Proportion of generated explanation claims linked to at least one retained source span |
| Evidence correctness | Clause-level review-flag instance | Proportion of cited evidence links that match the finalized evidence-span and relation references |
| Reference-component precision | Clause-level review-flag instance | Correctly recovered reference components divided by all components stated in the explanation |
| Reference-component completeness | Clause-level review-flag instance | Correctly recovered reference components divided by all applicable reference components |

These metrics measure agreement with benchmark-derived evidence components and do not establish that an explanation is causally valid, fair, legally correct, or operationally useful.

**B.16. Reproducibility Records**

**Table B17. Retained experiment fields.**

| **Field** | **Description** |
| --- | --- |
| random_seed | One of 13, 21, 42, 87, or 100 |
| configuration_id | Stable identifier for a complete validation configuration |
| selection_metric | Five-category validation Macro-F1 |
| validation_results | Macro-F1 and all complementary metrics for every evaluated setting |
| scalar_coefficient_candidates | Candidate values for $\lambda_{1}$and $\mu$ |
| selected_scalar_coefficients | Validation-selected coefficient values |
| barrier_category_weights | Fixed $\omega_{b}$values |
| accessibility_safeguard_weights | Fixed $\omega_{h}$values |
| service_support_weights | Fixed $\nu_{s}$values |
| group_priority_weights | Fixed $\pi_{g}$values |
| applicability_status | Applicable, not applicable, unresolved, or excluded |
| matched_category_ids | Categories supported by valid typed relations |
| evidence_span_ids | Source spans supporting each retained entity or relation |
| ordinal_cutpoints | Four fixed cutpoints per review dimension |
| split_file_version | Version of the family-safe training, validation, and test partition |
| prompt_and_model_version | Exact prompt template and model checkpoint |
| perturbation_manifest | Perturbation operation, seed, offsets, hashes, and mappings |

The reconstruction package should additionally record source and document IDs, official source locators, retrieval queries and filters, source-revision checksums, duplicate and family links, normalized texts and offset mappings, clause IDs, annotation records, family-level split reassignments, preprocessing configurations, and quality-control logs. Exact archive identifiers and SHA-256 values should be inserted only after the deposited files have been frozen.

**B.17. Interpretation and Terminology**

The formal review dimensions are **executability**, **behavioral accessibility**, and **service-support coverage**. Their document-level symbols are $R_{i}^{exe}$, $R_{i}^{acc}$, and $R_{i}^{sup}$. No cross-dimensional overall risk score is constructed.

Higher values indicate greater text-implied expert-review priority under the predefined evidence-linked schema. They do not establish:

- actual policy implementation failure;
- realized behavioral inaccessibility;
- inadequate real-world service provision;
- distributive inequity;
- causal effects on a population;
- legal, regulatory, or clinical noncompliance; or
- professional-review effectiveness.

The preferred terms are **structural-gap review signal**, **review-signal score**, **high-priority review-signal F1**, and **text-implied service-support coverage**. The terms “risk score,” “high-risk classification,” “equity measurement,” and “expert-performance improvement” should not be used for these outputs.
